# Alliance A022104/NRG-GI010: The Janus Rectal Cancer Trial: a randomized phase II/III trial testing the efficacy of triplet versus doublet chemotherapy regarding clinical complete response and disease-free survival in patients with locally advanced rectal cancer

**DOI:** 10.1186/s12885-024-12529-7

**Published:** 2024-07-26

**Authors:** Janet A. Alvarez, Qian Shi, Arvind Dasari, Julio Garcia-Aguilar, Hanna Sanoff, Thomas J. George, Theodore Hong, Greg Yothers, Philip Philip, Garth Nelson, Tareq Al Baghdadi, Olatunji B. Alese, Wini Zambare, Dana Omer, Floris S. Verheij, Aron Bercz, Min Jung Kim, James Buckley, Hannah Williams, Manju George, Reese Garcia, Phuong Gallagher, Eileen M. O’Reilly, Jeffrey A. Meyerhardt, Jamie Crawley, Ardaman Shergill, Natally Horvat, Paul B. Romesser, William Hall, J. Joshua Smith

**Affiliations:** 1 Memorial Sloan Kettering Cancer Center, 1275 York Avenue, SR-201, New York, NY 10065 USA; 2 Mayo Clinic, Rochester, MN USA; 3 University of Texas MD Anderson Cancer Center, Houston, TX USA; 4 UNC Lineberger Comprehensive Cancer Center, Chapel Hill, NC USA; 5 University of Florida Health Cancer Center, University of Florida, Gainesville, FL USA; 6 Massachusetts General Hospital, Boston, MA USA; 7 University of Pittsburgh, Pittsburgh, PA USA; 8 Henry Ford Cancer Institute, Detroit, MI USA; 9 Trinity Health IHA Medical Group, Ann Arbor, MI USA; 10 Winship Cancer Institute of Emory University, Atlanta, GA USA; 11 COLONTOWN/Paltown Development Foundation, Crownsville, MD USA; 12 Fight Colorectal Cancer, Springfield, MO USA; 13 Dana-Farber/Partners CancerCare, Boston, MA USA; 14 University of Chicago, Chicago, IL USA; 15 Medical College of Wisconsin, Milwaukee, WI USA

**Keywords:** Clinical complete response, Locally advanced rectal cancer, Organ preservation, Total neoadjuvant therapy, Watch and wait/active surveillance

## Abstract

**Background:**

Recent data have demonstrated that in locally advanced rectal cancer (LARC), a total neoadjuvant therapy (TNT) approach improves compliance with chemotherapy and increases rates of tumor response compared to neoadjuvant chemoradiation (CRT) alone. They further indicate that the optimal sequencing of TNT involves consolidation (rather than induction) chemotherapy to optimize complete response rates. Data, largely from retrospective studies, have also shown that patients with clinical complete response (cCR) after TNT may be managed safely with the watch and wait approach (WW) instead of preemptive total mesorectal resection (TME). However, the optimal consolidation chemotherapy regimen to achieve cCR has not been established, and a randomized clinical trial has not robustly evaluated cCR as a primary endpoint. Collaborating with a multidisciplinary oncology team and patient groups, we designed this NCI-sponsored study of chemotherapy intensification to address these issues and to drive up cCR rates, to provide opportunity for organ preservation, improve quality of life for patients and improve survival outcomes.

**Methods:**

In this NCI-sponsored multi-group randomized, seamless phase II/III trial (1:1), up to 760 patients with LARC, T4N0, any T with node positive disease (any T, N +) or T3N0 requiring abdominoperineal resection or coloanal anastomosis and distal margin within 12 cm of anal verge will be enrolled. Stratification factors include tumor stage (T4 vs T1-3), nodal stage (N + vs N0) and distance from anal verge (0–4; 4–8; 8–12 cm). Patients will be randomized to receive neoadjuvant long-course chemoradiation (LCRT) followed by consolidation doublet (mFOLFOX6 or CAPOX) or triplet chemotherapy (mFOLFIRINOX) for 3–4 months. LCRT in both arms involves 4500 cGy in 25 fractions over 5 weeks + 900 cGy boost in 5 fractions with a fluoropyrimidine (capecitabine preferred). Patients will undergo assessment 8–12 (± 4) weeks post-TNT completion. The primary endpoint for the phase II portion will compare cCR between treatment arms. A total number of 312 evaluable patients (156 per arm) will provide statistical power of 90.5% to detect a 17% increase in cCR rate, at a one-sided alpha = 0.048. The primary endpoint for the phase III portion will compare disease-free survival (DFS) between treatment arms. A total of 285 DFS events will provide 85% power to detect an effect size of hazard ratio 0.70 at a one-sided alpha of 0.025, requiring enrollment of 760 patients (380 per arm). Secondary objectives include time-to event outcomes (overall survival, organ preservation time and time to distant metastasis) and adverse event rates. Biospecimens including archival tumor tissue, plasma and buffy coat, and serial rectal MRIs will be collected for exploratory correlative research. This study, activated in late 2022, is open across the NCTN and had accrued 330 patients as of May 2024. Study support: U10CA180821, U10CA180882, U24 CA196171; https://acknowledgments.alliancefound.org.

**Discussion:**

Building on data from modern day rectal cancer trials and patient input from national advocacy groups, we have designed The Janus Rectal Cancer Trial studying chemotherapy intensification via a consolidation chemotherapy approach with the intent to enhance cCR and DFS rates, increase organ preservation rates, and improve quality of life for patients with rectal cancer.

**Trial registration:**

Clinicaltrials.gov ID: NCT05610163; Support includes U10CA180868 (NRG) and U10CA180888 (SWOG).

**Supplementary Information:**

The online version contains supplementary material available at 10.1186/s12885-024-12529-7.

## Background

The use of total neoadjuvant therapy (TNT) is now at the forefront for patients with locally advanced rectal cancer (LARC) [1–9]. The TNT treatment paradigm involves the delivery of both chemoradiation (CRT) and systemic chemotherapy in the neoadjuvant setting. There is mounting evidence that TNT leads to higher clinical and pathologic complete response (pCR) rates with improved treatment adherence, and provides a unique opportunity to assess biological response on an individual patient basis [2, 6, 9, 10]. 

As TNT has resulted in increased clinical complete response (cCR) rates, the need for surgery in patients with a cCR has been called into question, with increased interest in organ preservation and watch and wait (WW)/active surveillance strategies. It has long been known that patients with a pCR to preoperative CRT have lower tumor recurrence rates and improved survival compared to patients without a pCR, thus raising questions about the added value of total mesorectal excision (TME) for these individuals [11–13]. Habr-Gama et al. were the first to report on the safety and efficacy of WW in patients with a cCR after CRT in 2004, noting 26% of patients were able to avoid surgery with a durable complete response 10 years from CRT alone [14]. Since then, multiple, large retrospective institutional case series and more recent prospective data suggest that WW can be safely incorporated without compromising oncologic outcomes [9, 15].

The Organ Preservation in Patients with Rectal Adenocarcinoma (OPRA) [9] trial was a prospective, multicenter phase II clinical trial in which patients with stage II/III rectal cancer were randomized to receive either induction long course chemoradiation (LCRT) followed by consolidation chemotherapy or induction chemotherapy followed by consolidation LCRT. Patients subsequently underwent TME or were offered surveillance via a WW protocol based on tumor response [15]. The disease-free survival (DFS), overall survival (OS), local and distant recurrence-free survival were similar to patients treated with standard LCRT, TME, and adjuvant chemotherapy at both 3 and 5 years of follow-up [9, 16]. Approximately half of all patients treated with TNT achieved a cCR and were managed by active surveillance rather than surgery. The use of induction LCRT followed by consolidation chemotherapy resulted in a higher rate of 3-and-5-year organ preservation compared to induction chemotherapy followed by consolidation LCRT [9, 16].

Here we report on the details of The Janus Rectal Cancer Trial (NCT05610163), a National Clinical Trials Network (NCTN) Phase II/III trial testing the optimal TNT regimen using a consolidation chemotherapy approach of triplet versus doublet chemotherapy based on the hypothesis that a triplet chemotherapy regimen after induction LCRT will demonstrate superior cCR rates and DFS outcomes compared to a doublet chemotherapy regimen after induction LCRT**.** The Janus Rectal Cancer Trial is important for our rectal cancer patients as it builds on the findings of modern rectal cancer trials to move the field forward in validation of the cCR endpoint and to enhance quality of life for patients through increased rates of organ preservation using a chemotherapy intensification TNT approach [9, 10]. Furthermore, the Phase III portion has been designed to test whether triplet versus doublet chemotherapy will improve DFS. During protocol development, The Janus Rectal Cancer Trial study development team received input from two separate patient advocate groups and clinicians, noting that 76% of respondents preferred a chemotherapy intensification approach to a radiation escalation approach (Alvarez J, George M, Garcia R, et al. *unpublished*). Based on OPRA data and patient input, we have designed the current trial studying chemotherapy intensification via a consolidation chemotherapy approach with the intent to enhance cCR and DFS rates, increase organ preservation rates, and thereby improve quality of life for patients with rectal cancer.

## Methods

### Participants, interventions, and endpoints

#### Study setting

The Janus Rectal Cancer Trial is organized through the Alliance for Clinical Trials in Oncology, sponsored by the National Cancer Institute (NCI) and administered through the NCTN. It is unique in that the study has integrated collaboration in both design and leadership across the NCI-NCTN inclusive of the Alliance for Clinical Trials in Oncology (overall PI and Study Chair, Smith), NRG Oncology (co-PI, Hall), SWOG (co-PI, Dasari), and ECOG (Study Champion, Alese). ClinicalTrials.gov Identifier: NCT05610163.

#### Patient selection and eligibility

Patients will be recruited and consented to the study in colorectal surgery, surgical oncology, medical oncology, and radiation oncology clinics. To participate, patients must have a biopsy-proven clinical diagnosis of stage II or III (T4N0 or any T, node-positive disease) mismatch repair proficient (pMMR) adenocarcinoma of the rectum located 12 cm or less from the anal verge. Patients are only eligible if they have received no prior systemic chemotherapy, targeted therapy, immunotherapy, or radiation therapy administered as a treatment for colorectal cancer within the past five years and are older than 18 years old. Additional inclusion and exclusion criteria are provided in Table 1. Required eligibility testing will be completed, which includes the baseline tumor and characteristic documented at < = 12 cm from the anal verge via flexible sigmoidoscopy, a pelvic MRI with dedicated rectal protocol, and a biopsy completed if needed to confirm pMMR adenocarcinoma. Informed consent is obtained for eligible patients. The patient's eligibility checklist is verified by the local study team and then the patient is enrolled onto the trial and randomized 1:1 to either the experimental arm (triplet therapy) or the control arm (doublet therapy).

**Table 1:** Inclusion and exclusion criteria

Inclusion Criteria		Exclusion Criteria
General	Histologically confirmed diagnosis of adenocarcinoma of the rectum Patients must have clinical Stage II or III rectal adenocarcinoma defined as T4N0 or any T with node positive disease (any T, N +); also, T3N0 requiring APR or coloanal anastomosis Tumor site $\le 12\text{cm}$ from the anal verge Age $\ge$ 18 years No prior systemic chemotherapy, targeted therapy, or immunotherapy, or radiation therapy administered as treatment for colorectal cancer within the past 5 years Karnofksy $\ge$ 60%, ECOG $\le$ 2, ANC $\ge$ 1,500/mm^3^, Platelet $\ge$ 100,000/mm^3^, Creatinine $\le$ 1.5 × upper limit of normal OR Calc. Creatinine Clearance $\ge$ 50 mL/min, Total bilirubin $\le$ 1.5 × upper limit of normal, AST/ALT $\le$ 3 × upper limit of normal HIV infected patients on effective anti-retroviral therapy with undetectable viral load within 6 months Patients with known history or current symptoms of cardiac disease, or history of treatment with cardiotoxic agents, should have a clinical risk assessment or cardiac function using the New York Heart Association Classification. Patients should be Class 2B or better	Recurrent rectal cancer Upper rectal cancers (distal margin of tumor $>$ 12 cm from the anal verge Prior distal sigmoid cancer with a low anastomosis Prior trans-anal excision Known mismatch repair deficient rectal adenocarcinoma Chronic concomitant treatment with strong inhibitors of CYP3A4 that can not be discontinued 14 days prior to study registration and for duration of the study Chronic concomitant treatment with strong inducers of CYP3A4 that can not be discontinued 14 days prior to study registration and for duration of the study
Consent	Patients must read, agree to, and sign a statement of Informed Consent prior to participation in this study. Patients who do not read or understand English are eligible but must have the consent form read to them in its entirety by an official translator, either from the study site or via phone interpreter. Informed consent for non-literate or non-English speaking patients may not be obtained by using a relative or a member of the patient’s clinical team as a translator. Consortium sites must follow federal, local, and institutional regulations to ensure that non-English speaking patients are consented appropriately	N/A
Women	Women of childbearing potential who are negative for pregnancy test (urine or blood), not nursing, and who agree to use effective contraceptive methods. A woman of childbearing potential is defined as a sexually mature female who has not undergone hysterectomy or bilateral oophorectomy and has not been postmenopausal for 12 consecutive months	Women are pregnant or breast-feeding. Women of childbearing potential who are unwilling or unable to use an acceptable method of birth control to avoid pregnancy for the entire study period and for $\ge$ 9 months after the last dose of study drug
Men	Male subjects must also agree to effective contraception	Men who are unwilling or unable to use an acceptable method of birth control while in this study and for $\ge$ 6 months after the last treatment

#### Study design

**Fig. 1:**
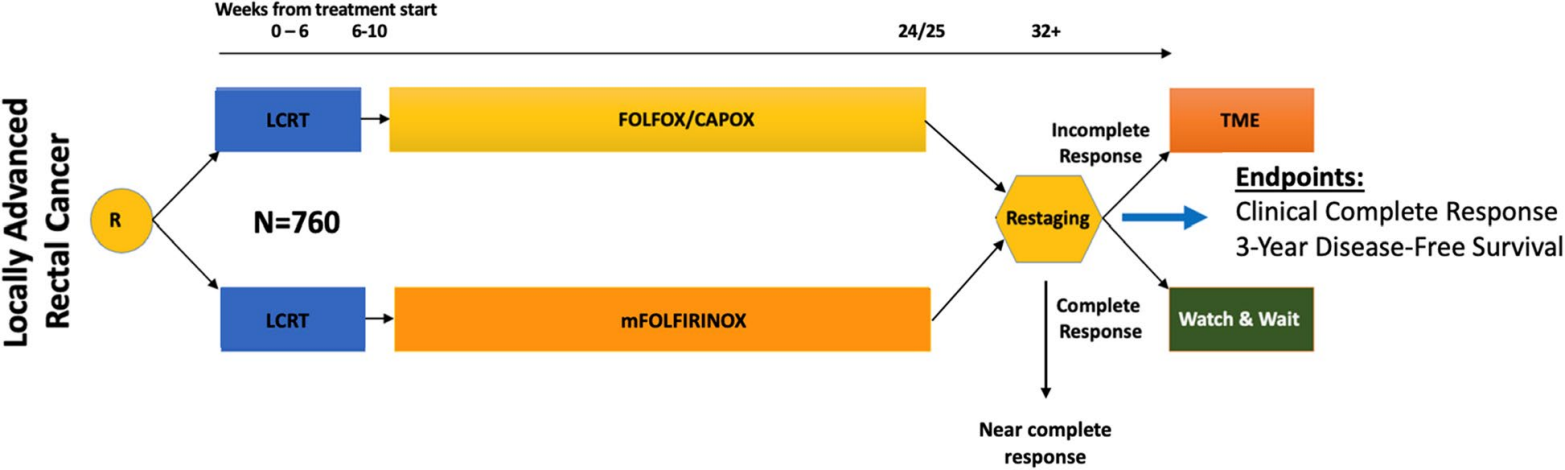
The Janus Rectal Cancer Trial Schema. Key: Randomization = R; LCRT = long-course chemoradiation; Restaging determination = endoscopy, MRI and clinical exam 8–12 (± 4) weeks post-completion of assigned TNT regimen, LARC < = 12 cm, cT4N0, any T, N + ; T3N0 that would require APR or coloanal anastomosis

The Janus Rectal Cancer Trial is a two arm, national, randomized, seamless phase II/III study investigating the effect of LCRT followed by either triplet chemotherapy or doublet chemotherapy in patients with LARC. The study was initially designed as a Phase II trial to test the hypothesis that triplet versus double chemotherapy after LCRT would improve cCR by 17% (from 50% for the control; power of 90% and one-sided alpha 0.048) yielding 312 patients for evaluation. The study was recently amended to a definitive phase III with a DFS primary endpoint (power 85%, one-sided alpha 0.025) for a total of 760 patients. The full study schema is illustrated in Fig. 1.

#### Treatment plan/intervention

Protocol therapy will consist of induction LCRT followed by consolidation chemotherapy. Induction LCRT includes radiation (45 Gy + 9 Gy boost in 27–30 fractions) in combination with a concomitantly administered fluoropyrimidine (preferred capecitabine; permissible substitution: continuous infusion 5-fluorouracil). Subsequently, patients will receive eight cycles of consolidation chemotherapy with either mFOLFOX6 (may be substituted by 5 cycles of CAPOX) in the control arm (Arm B) or eight cycles of mFOLFIRINOX in the experimental arm (Arm A). All patients will undergo assessment 8–12 (± 4) weeks post-completion of all therapy for the primary endpoint of cCR for the phase II portion. Patients who have an incomplete response will require TME, while patients who achieve a cCR will be recommended further management with WW. Uniquely, patients with a near complete response (nCR) will be recommended repeat assessment in 4–8 weeks and offered WW versus TME depending on their final response. If the tumor fails to evolve to a cCR then they will be recommended TME.

#### Primary endpoints

The primary endpoints of the The Janus Rectal Cancer Trial are to compare cCR rates and DFS between the two treatment groups for phase II and III portions, respectively. For the Phase II portion, the cCR rate is defined as the proportion of patients who achieved cCR at the end of TNT or who progressed to a cCR after nCR and re-evaluation. For the phase III portion, DFS is defined as time from date of randomization to the date of first occurrence of death due to all causes, tumor that recurs locally after an R0 resection TME, tumor that regrows after an initial apparent clinical and radiological complete response and cannot be surgically removed with an R0 resection TME, and/or M1 disease diagnosed at any point after the initiation of treatment. Note that local tumor regrowth that can be surgically removed with a R0 resection TME will not be a DFS event.

#### Secondary endpoints

Secondary endpoints include organ-preservation-time, time to distant metastasis, OS, and rate of adverse events (AEs). Organ-preservation time is defined as time from the date of randomization to the date of the first occurrence of TME (including successful or attempted and failed TME), tumor that regrows after an initial apparent clinical and radiological complete response, and death due to all causes. Time to distant metastasis is defined as time from the date of randomization to the date of first documented distant metastasis. OS is defined as time from the date of randomization to the date of death due to all causes. The rate of AEs is defined as the proportion of patients experienced at least one Grade 3, Grade 4, or Grade 5 of each type of AE.

#### Exploratory objective

Circulating tumor DNA (ctDNA) will be obtained from patients with consent during TNT and surveillance with the aim to correlate values with radiographic, pathologic, and clinical outcomes. The field of ctDNA assay development is rapidly evolving. Our study team will encourage prospective tissue and blood banking to then select the most appropriate assay based on sample availability and performance characteristics closer to the end of full study enrollment (at least 80%). Further details on the biobanking protocol are included in the full protocol included in supplementary material.

#### Participant timeline

Laboratory and clinical parameters during treatment are to be followed using individual institutional guidelines and the best clinical judgment of the responsible physician. It is expected that patients on this study will be cared for by physicians experienced in the treatment and supportive care of patients on this trial.

### Pre-study testing intervals

The pre-study testing intervals are guidelines only. When calculating days of tests and measurements, the day a test or measurement is done is considered Day 0. Therefore, if a test were done on a Monday, the Monday one week later would be considered Day 7.

- To be completed ≤ 28 DAYS before registration: All laboratory studies, history and physical, performance status, pregnancy test.
- To be completed ≤ 42 DAYS before registration: Any X-ray, scan of any type or ultrasound which is utilized for tumor measurement per protocol.
- To be completed ≤ 60 DAYS before registration: Any baseline exams used for screening, or any X-ray, scan of any type or ultrasound of uninvolved organs which is not utilized for tumor measurement.

Please refer to Table 2 for the complete Study Calendar for both arms.

**Table 2:** Study calendars

**ARM A—Experimental**																				
**Evaluations during treatment – (LCRT THEN mFOLFIRINOX)**																				
Study Week (± 14 days)^a^	Pre		10^k^		12		14		16		18		20		22		24		32–38^f^	
History and physical^b^	X		X		X		X		X		X		X		X		X		X	
Height	X																			
Adverse Event Assessment	X		X		X		X		X		X		X		X		X		X	
Colorectal surgeon eval	X		X																X	
Med Onc^g^	X		X		X		X		X		X		X		X		X		X	
Rad Onc	X		X																	
DRE(digital rectal exam)	X		X																X	
Sigmoidoscopy/Proctosigmoidoscopy^c^	X		X																X	
Biopsy^h^	X																			
MRI Rectum	X																		X	
CT CAP^i^	X																		X	
CBC & diff^j^	X																			
CMP & CEA	X		X																X	
Pregnancy Test^d^	X																			
**ARM B—Control**																				
**Evaluations during treatment – (LCRT THEN FOLFOX OR LCRT THEN CAPOX)**																				
Study Week (± 14 days)^a^		Pre		10^k^		12		14		16		18		20		22		24		32–38^f^
History and physical^b^		X		X		X		X		X		X		X		X		X		X
Height		X																		
Adverse Event Assessment		X		X		X		X		X		X		X		X		X		X
Colorectal surgeon eval		X		X																X
Med Onc^g^		X		X		X		X		X		X		X		X		X		X
Rad Onc		X		X																
DRE (digital rectal exam)		X		X																X
Sigmoidoscopy/Proctosigmoidoscopy^c^		X		X																X
Biopsy^h^		X																		
MRI Rectum		X																		X
CT CAP^i^		X																		X
CBC & diff^j^		X																		
CMP & CEA		X		X																X
Pregnancy Test^d^		X																		

^a^Timing can vary based on institutional standards (for example, if a center waits longer than 14 days between starting chemotherapy after completion of LCRT this is not a protocol violation) as some centers wait 4–6 weeks after LCRT completion to start systemic chemotherapy. As such, exact week number from registration may vary and as such can be adjusted to reflect timing of start of chemotherapy

^b^Weight, Pulse, BP. Performance Status will only be required at pre, week 10 and weeks 32–38

^c^The flexible sigmoidoscopy/proctosigmoidoscopy serves as a key feature for the baseline and final response of the primary tumor to the treatments assigned. The flexible sigmoidoscopy/proctosigmoidoscopy SHOULD NOT/MAY NOT be completed by the referring gastroenterologist BUT SHOULD BE/MUST BE completed by the evaluating/treating surgeon for baseline evaluation and during treatment assessments to maintain continuity of the response assessment (as this is critical for the primary endpoint of clinical complete response)

Baseline characteristics that should be captured include distance from the anal verge, photos, and the percent circumference of the lumen involved by the tumor. If the surgeon evaluating the patient at baseline WAS THE PHYSICIAN WHO completed the colonoscopy that was diagnostic then this would be sufficient for the initial baseline endoscopic evaluation as long the baseline characteristics of the tumor were captured including distance from the anal verge, photos and baseline characteristics of the tumor (as above). Given the importance for the primary endpoint it is critical for a surgeon to be involved who is willing to evaluate the primary tumor for the endpoint throughout the duration of the trial. The surgeon involved should be affiliated with an NCTN hospital or hospital system. It is permissible for the evaluating surgeon to be at a separate institution from the treating medical and radiation oncology teams as long as there is continuity in the management and seamless sharing of relevant clinical data between the teams (assuming all teams are part of NCTN or NCTN-affiliated hospitals and the arrangement is convenient for the patient). Further, the evaluating surgeon must have access to the protocol, all relevant documents and the tumor response forms to participate

^d^For women of childbearing potential (see Sect. 3.2.3 in study protocol/supplementary material). Must be done ≤ 7 days prior to registration

^e^Time of evaluation dependent on duration of neoadjuvant chemotherapy FOLFOX (16 weeks) or CAPOX (15 weeks)

^f^8–12 weeks (± 4 weeks) after completion of all neoadjuvant therapy

^g^Patients will be seen and evaluated during neoadjuvant chemoradiation and chemotherapy with necessary laboratory evaluations per institutional guidelines. For neoadjuvant chemotherapy, recommendation is to be evaluated by medical oncology during (Arm 1) and (Arm 2) chemotherapy every two weeks (every three weeks for patients getting CAPOX) or as needed per institutional guidelines. In patients not seen and evaluated by medical oncology every cycle, adverse events must still be collected and reported every cycle by relevant research staff

^h^Biopsy to confirm pathological diagnosis of rectal adenocarcinoma is REQUIRED

^i^CT of the Chest, Abdomen and Pelvis. Prefer with intravenous contrast, but per institutional standards based on patient’s labs and medical condition

^j^CBC & diff, CMP performed at baseline and with each cycle of chemotherapy or per institutional standards

^k^Assessment by surgeon, medical and radiation oncology occurs in a multidisciplinary fashion to ensure the patient has tolerated LCRT well and does not have to occur exactly at week 10; SHOULD BE PLANNED and completed prior to initiation of chemotherapy and is meant to ensure appropriate transition to the medical oncology team as they begin systemic chemotherapy. If the patient cannot tolerate a DRE and flexible sigmoidoscopy prior to chemotherapy initiation, the study team should document this; however, the patient must undergo an exam by all groups (surgery, medical oncology and radiation oncology) along with the scheduled laboratory tests as scheduled prior to chemotherapy initiation

### Sample size

For the phase II portion, a total of 296 evaluable patients (148 per arm) will be needed to evaluate cCR rate. An additional 16 patients (5% inflation) will be accrued to account for cancellation after randomization and major violations. The total target accrual will be up to **312** patients.

For the phase III portion, total sample size is **760** patients or 380 per arm. Estimated accrual rate is 180 patients per year. Accrual as of May 2024 is 330.

## Assignment of interventions

### Randomization and stratification factors

Consenting and eligible patients will be registered to the study. Stratification factors will be recorded including clinical tumor stage (T4 versus T1-3), clinical nodal stage (N + versus N0), and distance from the lower edge of the tumor to the anal verge (0 to < 4 cm; ≥ 4 cm to < 8 cm; ≥ 8 cm to ≤ 12 cm). Patients will be randomly assigned in a 1:1 ratio to one of the following treatment groups:

1. Induction LCRT followed by consolidation mFOLFIRINOX (Arm A): experimental arm
2. Induction LCRT followed by consolidation mFOLFOX6 or CAPOX (Arm B): control arm

## Statistical methods

The Phase II portion of this trial implements a group sequential design with a single interim analysis for futility evaluation, adopting Rho family (Rho = 2) beta spending function for controlling the overall type II error rate.

The OPRA trial reported 52.4% (87 out of 166 randomized to consolidation chemotherapy arm) of patients who achieved a sustained cCR and preserved the rectum. For the proposed trial, we assume a cCR rate of 50% in the control arm (Arm B). A total number of 296 evaluable patients will provide 90.5% power to detect a 17% increase in cCR rate (67% in the experimental arm [Arm A]) at a one-sided type I error rate of 0.048. The total number of patients accounting for violations or cancellations (5%) is 312.

The Phase III portion implements a group sequential design with one futility interim analysis based on a non-binding beta spending function (Rho family with Rho = 3.2), which will be performed when 50% of DFS events have been observed (143 events). The OPRA trial reported a three-year DFS rate of 76% (95% CI, 69–83%) for patients who received LCRT followed by consolidation chemotherapy [9]. A total number of 285 DFS events will provide 85% power to detect an effect size of hazard ratio (HR) = 0.70 (3-year DFS rate of 82.5% in the experimental arm A) at a one-sided type I error rate of 0.025. With further assumptions of an accrual rate of 180 patients per year and a minimum of four years of follow-up, a maximum of 760 patients (380 in each arm) are required to enroll, unless the study team makes a decision of early termination (as noted in the monitoring rules specified in the supplementary protocol).

For the phase II primary endpoint of cCR rate, hypothesis testing will be performed on the modified intent-to-treat (mITT) population defined as all patients properly randomized, completed LCRT and who started at least one dose of protocol defined chemotherapy treatment, with treatment grouping according to the original assignment at randomization. Sensitivity analysis will be performed on the per protocol (PP) population defined as all patients properly randomized who started at least three cycles of chemotherapy after LCRT, with treatment grouping according to actual treatment received during the first cycle of chemotherapy. An interim analysis for futility will be performed when 50% of patients in each arm (74 patients) are randomized and cCR status is determined. The analysis of the phase III primary endpoint of DFS will be performed on intention-to-treat population defined as all patients who are properly randomized, regardless of the actual treatment received. The treatment grouping will be according to the original assignment at randomization. Sensitivity analyses will be performed on mITT and PP population. At interim and final analyses, stratified Cox model will be conducted to compare DFS in the experimental arm to DFS in the control arm with stratification factors as stratum, based on all data collected at the analysis time point.

The analysis of secondary endpoints will be on mITT and PP population with the Kaplan–Meier method and stratified Cox regression models. The maximum grade for each type of AE related to study treatment will be recorded and reviewed to determine patterns. The overall AE rates for grade 3 or higher AEs will be compared between two treatment groups using Chi-square test (or Fisher’s exact test if the data in the contingency table is sparse).

## Monitoring

### Response evaluation

Patients will undergo assessment for tumor response at 8–12 (± 4) weeks post-completion of TNT. Patients with a cCR as determined by the MSK Regression Schema [9, 15] (no tumor on clinical exam, endoscopy, or MRI) may be offered a WW approach or TME depending on the outcome of an in-depth discussion and understanding of the risks and benefits of each approach. Patients with an incomplete response as determined by the MSK Regression Schema (any evidence of residual tumor on clinical exam, endoscopy, or MRI) will be recommended to undergo a TME. Similar to guidelines in the OPRA trial, if patients have a near complete response (nCR) they can undergo repeat assessment 4–8 weeks later. If there is evidence the tumor has stopped responding, continues to persist, or regrows then the patient will be recommended to undergo a TME. Endoscopy will be the deciding factor on determination of cCR if there is a discrepancy between clinical exam and MRI findings.

### Neoadjuvant treatment completion monitoring

The completion of neoadjuvant treatment will be closely monitored. We will compare early off treatment rates between both treatment arms at select timepoints. If the difference in early off treatment rate (experimental arm minus control arm) is greater than specified thresholds a formal review will be triggered and potential protocol modifications, including possible halting of accrual, will be formulated under consultation with the Cancer Therapy Evaluation Program (CTEP) and the study team.

### R0 resection for patients on WW/active surveillance monitoring

We will carefully monitor the R0 resection rate among patients who proceed to a WW strategy after TNT and later require TME during follow-up. Patients enrolled on both arms will be pooled for this monitoring. We have employed specific monitoring rules to test the hypothesis of whether an R0 resection rate in our population is adequate.

### Tumor regrowth

We will closely monitor tumor regrowth in patients who proceed to WW strategy after TNT. The five-year follow-up data from OPRA reported a 29% regrowth rate in patients randomized to induction LCRT and consolidation chemotherapy group who proceeded to a WW strategy [16]. Notably, 94% of local regrowth events occurred within the first 24 months [16]. The one-year regrowth rate will be defined as the number of patients who experience tumor regrowth (regardless of whether it can be salvaged by TME) within one year after the last dose of pre-operative TNT divided by the total number of patients in the analysis population. The one-year regrowth rate will be estimated within each arm separately, when all patients in this population are followed for at least one year after last dose of chemotherapy.

### Patient safety monitoring

The Study Chair(s) and the Study Statistician will review the study monthly to identify accrual, AE/safety trends, and any emerging concerns. The Study team will have monthly meetings to identify any issues that arise during the Phase II and Phase III portions of the study.

## Disease evaluation

### Measurement of Treatment Effect

Follow up after treatment consists of a schedule of endoscopy, digital rectal exam (DRE), CT Chest/Abdomen/Pelvis, MRI Pelvis, and CEA (Table 3). For specific surveillance intervals, refer to Table 4a and b for patients following WW protocol or for patients who are post-TME.

**Table 3:** Protocol duration of follow up

	**MRI rectum**	**Endoscopy and digital rectal exam**	**CT chest, abdomen, pelvis**	**CEA**
All patients who completed pre-operative protocol treatment	Pre-treatment, post-TNT re-staging	Pre-treatment, post-TNT re-staging	Pre-treatment, post-TNT re-staging, annually from 5 years post-registration	Pre-treatment, every 3–6 months post-TNT in Years 1–2, every 6 months post-TNT in Years 3–5
All patients who are off pre-operative protocol treatment early due to Progression of disease-	As per treatment team	As per treatment team	As per treatment team	As per treatment team
All patients who are off pre-operative protocol treatment early due to other reasons (patient refusal, clinician decision to withdraw/other)	As per treatment team	As per treatment team	As per treatment team	As per treatment team
Patients in WW group	After post-TNT re-staging: Q6 months in Years 1–2, Q12 months in Years 3–5 (As clinically indicated years 4–5)	Post-TNT re-staging: Q3 months in Years 1–2, Q6 months in Years 3–5	Pre-treatment, post-TNT re-staging, annually from 5 years post-registration	Pre-treatment, every 3–6 months post-TNT in Years 1–2, every 6 months post-TNT in Years 3–5

**Table 4:** Evaluations during surveillance for WW and post-TME

a: Evaluations during follow-up for WW patients (after completion of TNT with cCR and post-TNT restaging)																												
**Years on study**	**Year 1 (Every 3 months)**									**Year 2 (Every 3 months)**							**Year 3 (Every 6 months)**					Years 4–5* (Every 6 months)						
Months after post-TNT restaging (± 30 days)	**3**			**6**		**9**		**12**		**15**		**18**		**21**		**24**	**30**			**36**		**42**		**48**		**54**		**60**
History and Physical	X			X		X		X		X		X		X		X	X			X		X		X		X		X
Sigmoidoscopy/Proctosigmoidoscopy	X			X		X		X		X		X		X		X	X			X		X		X		X		X
MRI Rectum				X				X				X				X				X				X				X
CT CAP^1^								X								X				X				X				X
CEA levels^2^	X			X				X		X		X				X	X			X		X		X		X		X
b: Evaluations during follow-up for TME patients (after definitive surgical resection)^#^																												
**Years on study**		**Year 1 (Every 3 months)**							**Year 2 (Every 3 months)**									**Year 3 (Every 6 months)**					Years 4–5** (Every 6 months)					
Months after treatment (± 30 days)		**3**	**6**		**9**		**12**		**15**		**18**		**21**		**24**			**30**	**36**		**42**		**48**		**54**		**60**	
History and Physical		X	X		X		X		X		X		X		X			X	X		X		X		X		X	
Sigmoidoscopy/Proctosigmoidoscopy^**^							X								X				X				X				X	
CT CAP^1^			X				X				X				X				X				X				X	
CEA levels^2^		X	X		X		X		X		X		X		X			X	X		X		X		X		X	

^*^MRI in years 4 through 5 would only be supported if clinically indicated by treating team

^1^CT of the Chest, Abdomen and Pelvis if regrowth occurs and TME completed then patients will be followed as per NCCN guidelines

^2^After 24 months, CEA will be evaluated every 6 months up to five years, based on NCCN guidelines

^#^Institutional guidelines for surveillance can be followed post-TME in accordance with NCCN guidelines or as clinically indicated per the discretion of the treating physicians

^**^If TME completed via abdominoperineal resection – A FLEXIBLE SIGMOIDOSCOPY/PROCTOSIGMOIDOSCOPY IS not relevant and unnecessary to assess the anastomosis for recurrence

^1^CT of the Chest, Abdomen and Pelvis: TME group patients will be followed according to NCCN guidelines

^2^After 24 months, CEA will be evaluated every 6 months up to five years, based on NCCN guidelines

### Clinical tumor evaluation

On endoscopy, the length of the tumor is defined as the difference between the distance of the proximal and distal margins in relation to the anal verge. Endoscopic tumor response will be determined by the MSK Regression Schema (Table 5). For patients who ultimately undergo TME after TNT, clinical tumor evaluations with DRE, endoscopy, and MRI will determine the need for TME. For patients who elect for a WW approach, clinical tumor evaluations with DRE, endoscopy, MRI, CT Chest/Abdomen/Pelvis, and CEA will occur during the post-TNT follow-up, up to 5 years post-randomization, or up to salvage TME, whichever occurs first (Table 3).

**Table 5:** The MSK Regression Schema. Please refer to the following video for endoscopic and MRI response assessment—https://www.youtube.com/watch?v=38rsqZvJIHg

MSK Regression Schema^e^			
	**Clinical Complete Response**	**Near Complete Response**	**Incomplete / No Response**
**Endoscopy**	Flat, white scar Telangiectasia No ulcer No nodularity	Irregular mucosa Superficial ulceration Mild persisting erythema of the scar	Visible tumor
**Digital Rectal Exam**	Normal^f^	Smooth induration or minor mucosal irregularity^f^	Palpable tumor nodules^f^
**MRI-T2WI**	Normal appearing rectal wall OR Only fibrosis (dark T2 signal) and no intermediate signal intensity at the site of tumor^a^ AND No suspicious lymph nodes^b^	Predominantly fibrosis at the site of tumor^a^ with punctate areas of T2 intermediate signal AND/OR No suspicious or borderline enlarged lymph nodes^b^	Predominantly residual tumor with T2 intermediate signal and no or minimal fibrosis at the site of tumor^a^ AND/OR Suspicious lymph nodes^b^ AND/OR Mucin at the site of tumor^d^
**MRI-DWI**	No restricted diffusion^c^ at the site of tumor^a^	Punctate areas of restricted diffusion^c^ at the site of tumor^a^	Restricted diffusion^c^ at the site of tumor^a^

^a^Site of tumor: rectal wall, extramural vascular invasion and/or tumor deposit.

^b^Suspicious lymph nodes criteria: (a) mesorectal and superior rectal nodes > 0.5 cm in the short axis; (b) internal iliac > 0.4 cm in the short axis particularly if suspicious on baseline; (c) obturator > 0.6 cm in the short axis particularly if suspicious on baseline; (c) mucin within the lymph nodes since MRI cannot distinguish cellular from acellular mucin. Additional lymph nodes should be interpreted cautiously, as there are no well-defined radiological criteria to strongly support their significance.

^c^Restricted diffusion: high signal on DWI high b-value (minimum b800) and low signal on ADC map.

*T2 dark through* (low signal on both DWI and ADC map) and *T2 shine thought* (high signal on both DWI and ADC map) effects are not considered restricted diffusion.

^d^MRI is unable to differentiate cellular from acellular mucin.

^e^Clinicians can also refer to the following website for examples of cCR, nCR, and iCR: https://nomtrial.mskcc.org/Home/Index

^f^Note not all tumors can be palpated (e.g., 10-12 cm from the anal verge) and thus the endoscopic features will be paramount and take precedent for decision-making in these mid-rectal tumors along with the MRI features

### The MSK Regression Schema

**Fig. 2:**
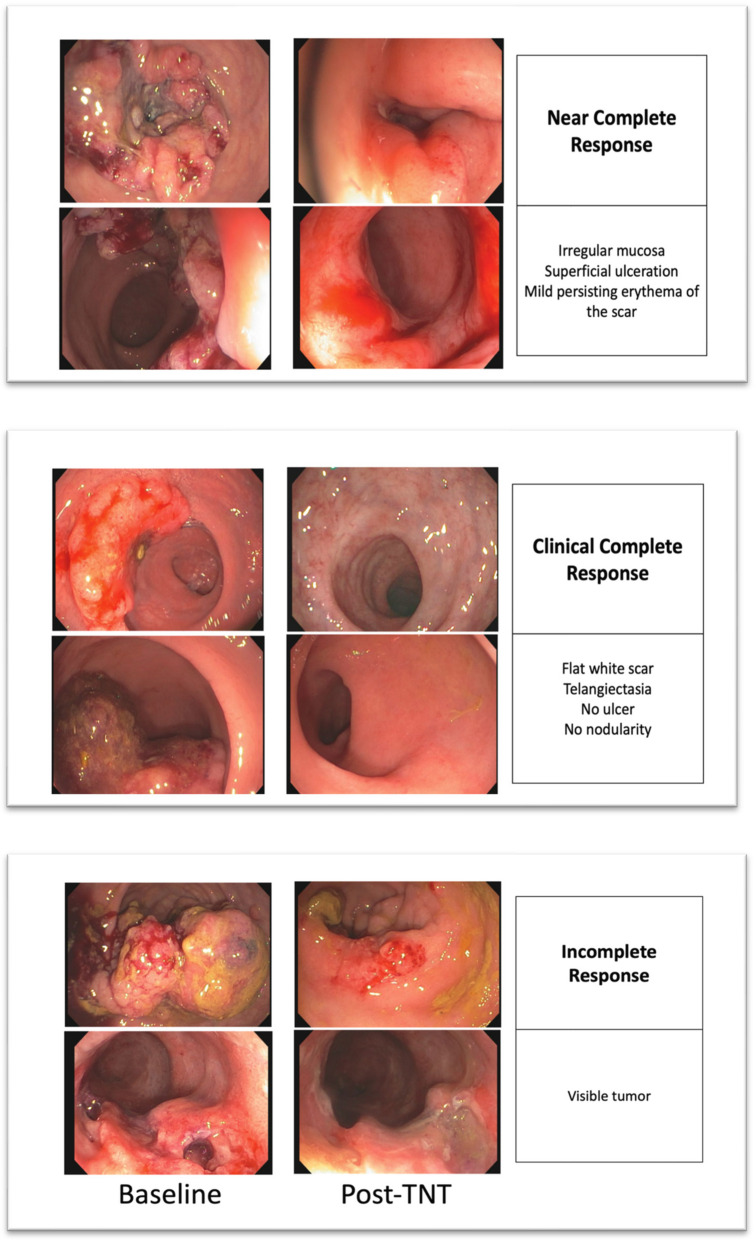
Endoscopic response images. Baseline and post-TNT endoscopy images showing a clinical complete, near complete, and incomplete response for patients who have completed TNT

**Fig. 3:**
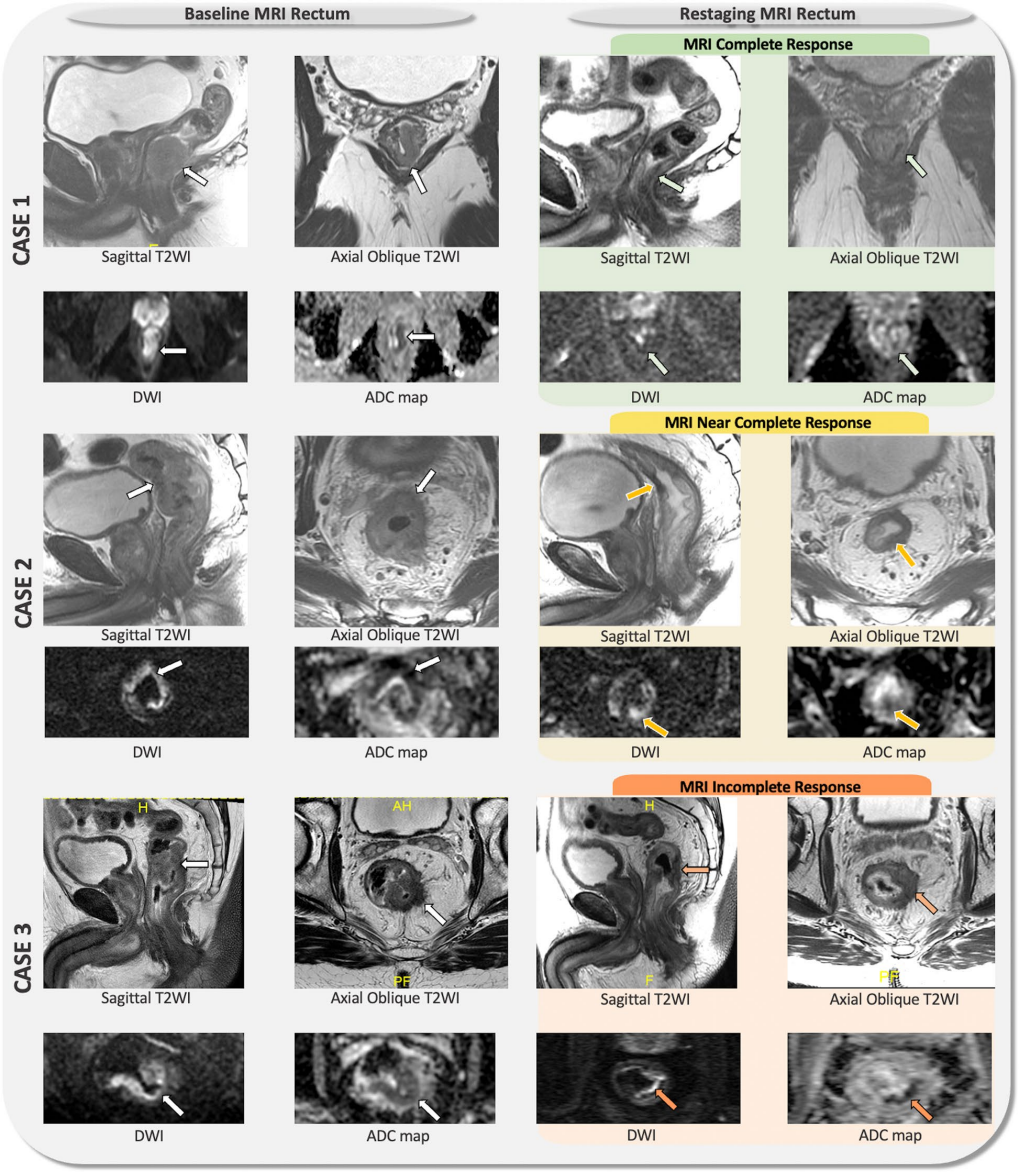
Baseline and re-staging MRI of the rectum of patients with complete response (case 1), near complete response (case 2) and incomplete response (case 3) based on the MRI assessment is shown. The white arrows on baseline MRI rectum indicate the primary rectal tumor, which shows intermediate signal intensity (SI) on T2-weighted imaging (T2WI), with high SI on diffusion-weighted imaging (DWI), and low SI on the ADC map, indicating restricted diffusion within the primary tumor.  Re-staging MRI on case 1 shows clinical complete response (green box) which is characterized by normalized rectal wall or only fibrosis (low signal intensity on T2WI, green arrows on T2WI) and no areas of restricted diffusion on DWI and ADC map (green arrows on DWI and ADC map).  Re-staging MRI on case 2 shows near complete response (yellow box) defined as small area of intermediate SI on T2WI within the tumor bed fibrosis (yellow arrows on T2WI) and small areas of restricted diffusion (yellow arrows on DWI and ADC map).  Re-staging MRI on case 3 shows incomplete response (orange box) characterized as significant areas of viable tumor with intermediate SI on T2WI within the tumor bed (orange arrows on T2WI) with restricted diffusion (orange arrows on DWI and ADC map). Reviewing the baseline MRI is highly recommended, as it helps to localize the tumor bed and guide the MRI to the appropriate angulation for high-resolution axial oblique T2WI acquisition perpendicular to the tumor bed

The MSK Regression Schema (Table 5) is based on subjective endoscopic and radiologic findings [9, 15]. It was developed by consensus with the aid of expert colorectal surgeons, medical oncologists, radiation oncologists, radiologists, and pathologists prior to the start of the OPRA trial to serve as a guideline to assess response and to provide uniformity in determining cCR, nCR, and incomplete/no tumor response after a patient has completed TNT (Fig. 2 and Fig. 3). Of note, as in OPRA, the endoscopic findings are deferred to for decision-making relative to cCR and decision for surveillance or TME versus what is found on MRI.

#### Radiologic tumor evaluation

Standard T2-weighted imaging (T2WI) and diffusion-weighted imaging (DWI) sequences will be obtained in 1.5 T or 3.0 T units using phased-array body coil. Expert radiologists from the patient’s primary treatment center will interpret all imaging studies according to the MSK Regression Schema [15]. Patients will require a baseline MRI and re-staging evaluations with MRI will be required of patients within the WW group (every 6 months for years 1–2, and every 12 months for year 3 and then as clinically indicated for years 4/5) to monitor closely for potential tumor regrowth. Refer to Table 6 which describes MRI features associated with local regrowth. Lastly, central radiology review is not required but imaging data will be centrally collected.

**Table 6:** Radiologic features suspicious for local or locoregional tumor regrowth on MRI

	**Radiological signs suspicious for local or locoregional tumor regrowth**
T2WI	New area of intermediate signal intensity on T2WI at the site of tumor AND/OR Increased size of previously suspicious lymph nodes AND/OR New suspicious lymph nodes AND/OR New areas of extramural vascular invasion or tumor deposit
DWI	New unequivocal areas of restricted diffusion in the tumor bed

Site of tumor: rectal wall, extramural vascular invasion and/or tumor deposit

#### Protocol follow up

Protocol intervention will continue until completion of LCRT and consolidation chemotherapy (8 cycles of FOLFOX or 5 cycles of CAPEOX), local/distant disease progression which preclude surgery, or unacceptable AEs. Patients who proceed to a WW strategy will be monitored as described in Table 3 for up to five years. TME will be performed as appropriate.

## Discussion

The Janus Rectal Cancer Trial expands on the findings of modern rectal cancer trials [4, 9, 10, 15] to provide further evidence to establish cCR as a robust endpoint and demonstrate improved patient outcomes with a consolidation chemotherapy intensification TNT approach. Preserving the rectum is a significant quality of life benefit for those patients who achieve a cCR and can progress to WW/active surveillance as it spares patients the morbidity of radical surgery and potential long term sequelae [17–20]. In addition, this trial will allow a venue to prospectively validate the MSK Regression Schema used in OPRA for assessing tumor response, and allow us to gain critical insight into the biology of response to consolidation TNT approaches in the context of standard clinical measures and novel correlative biomarkers.

Patients in The Janus Rectal Cancer Trial are randomized to induction LCRT followed by either mFOLFOX6/CAPEOX (doublet chemotherapy) or mFOLFIRINOX (triplet chemotherapy). Patients either proceed to surgery or WW based on tumor response. Multiple phase II and phase III clinical trials in metastatic colorectal cancer patients have compared doublet chemotherapy to triplet chemotherapy and have found consistently improved outcomes including objective radiographic response rates, OS and progression-free survival (PFS) [21–28]. Based on these results, the triplet regimen is included among first-line options in most clinical guidelines and recommendations worldwide [29–31]. More recently, the PRODIGE-23 trial enrolled 460 patients with LARC and randomized them to pre-operative CRT, TME, and adjuvant FOLFOX (control arm) versus induction mFOLFIRINOX followed by CRT, TME, and adjuvant FOLFOX (experimental arm) [4, 10]. The addition of 6 cycles of neoadjuvant mFOLIRINOX prior to CRT increased the pCR from 12 to 27%. Importantly, the 7-year updated data from PRODIGE-23 presented at ASCO 2023 demonstrated significantly better DFS, metastasis-free survival, and OS in the triplet TNT arm versus control arm (68% vs. 63% DFS). Together, these data convincingly show increased efficacy of triplet over doublet chemotherapy in patients with advanced colorectal cancer in improving R0 resection rates, objective response rates, PFS, DFS, and OS.

The Janus Rectal Cancer Trial will expand on the findings from OPRA which demonstrated improved organ preservation rates utilizing a consolidation chemotherapy approach [9]. The 5-year updated data in the OPRA trial has since resulted and demonstrated stable organ preservation rates for the consolidation chemotherapy arm (54%) versus the induction chemotherapy arm (39%, p = 0.012). Further, local regrowth rates remained lower in the consolidation chemotherapy arm (29%) versus in the induction chemotherapy arm (44%) (*p* = 0.02) [16]. The TIMING trial [7] reported increased pCR rates with additional cycles of FOLFOX in the consolidation setting. Additional evidence for the efficacy of the consolidation chemotherapy approach has been shown in the recent German trial/CAO/ARO/AIO-12 with acceptable pCR and superior complete response rates (25% pCR vs 17% pCR) compared to an induction chemotherapy approach [6, 32]. Building on these data and data from the OPRA trial, we anticipate that employing FOLFIRINOX in a consolidation approach after LCRT has the potential to further drive up response rates (increasing cCR rates) with an associated increase in long-term organ preservation rates.

Lastly, this study will measure ctDNA levels and study the potential use of ctDNA as an exploratory biomarker in the context of a prospective randomized trial. ctDNA levels will be used to measure response to treatment and may become a useful tool to help patients and clinicians choose TME versus WW. We also aim to develop a minimal residual disease-based risk classification for cCR patients. ctDNA has shown promise especially in the realm of colorectal cancer [33–38]. Multiple groups have reported worse recurrence-free survival in patients with positive ctDNA post-CRT, further supporting the utility of ctDNA during surveillance [33–36, 39]. Despite promising preliminary data, the kinetics of ctDNA after TNT, TME, and during surveillance and correlation with disease recurrence and overall survival has not been adequately studied. Our study serves as the optimal platform to study ctDNA as a predictive and prognostic biomarker in LARC. Additional predictive markers associated with complete response can be evaluated in future correlative studies on endoscopy, radiomics and spatial transcriptomics.

A major criticism of previously published WW data is that most of it comes from a select patient population treated at specialized centers. The recently reported OPRA trial was conducted across eighteen highly specialized academic centers, and thus provides the most robust, prospective data on outcomes of WW. While all OPRA sites were selected based on the expertise and clinical interest of the surgical team, The Janus Rectal Cancer Trial will determine generalizability of a WW approach across a more diverse population of patients, practice sites, and providers while incorporating a chemotherapy intensification approach in the context of modern TNT to improve response outcomes in a seamless phase II/III trial incorporating the WW strategy in a manner that will be acceptable to patients and clinicians. By running this trial through the NCI’s National Cancer Trial Network, this trial expands the opportunity to consider WW for patients treated at academic and community practices across the United States.

In summary, this study will explore the advantages of triplet versus doublet chemotherapy in LARC patients while expanding on findings from prior landmark trials by offering WW as an alternative to surgery in a national trial completed in a heterogeneous group of centers. We aim to optimize cCR rates via a chemo-intensification method, which was preferred by patients when surveyed in two separate patient advocate groups. In addition to cCR for the phase II portion, DFS will be the primary endpoint for our phase III portion. We will also evaluate and compare organ-preservation time, time to distant metastasis, OS, and toxicity profiles of TNT. Finally, we will conduct ctDNA surveillance and correlate it with patient outcomes, radiologic, and pathologic findings. Regardless of the trial results, this study has the potential to significantly impact the care of patients with LARC across the United States and abroad.

### Supplementary Information

Supplementary Material 1.

Supplementary Material 2.

## Data Availability

Not applicable.

## Abbreviations

cCR: Clinical complete response
CRT: Chemoradiation
ctDNA: Circulating tumor DNA
CTEP: Cancer Therapy Evaluation Program
DFS: Disease-free survival
DRE: Digital rectal examination
LARC: Locally advanced rectal cancer
LCRT: Long course chemoradiation
mITT: Modified intent-to-treat
NCI: National Cancer Institute
nCR: Near complete response
NCTN: National Clinical Trials Network
OPRA: Organ Preservation in Patients with Rectal Adenocarcinoma
OS: Overall survival
pCR: Pathologic complete response
PFS: Progression-free survival
PP: Per Protocol
TME: Total mesorectal excision
TNT: Total neoadjuvant therapy
WW: Watch and wait approach/Active surveillance
