# Supplementary material for: Alliance A022104/NRG-GI010: The Janus Rectal Cancer Trial: a randomized phase II/III trial testing the efficacy of triplet versus doublet chemotherapy regarding clinical complete response and disease-free survival in patients with locally advanced rectal cancer
Source: BMC Cancer. 2024 Jul 26;24:901. doi: 10.1186/s12885-024-12529-7 (PMC11282593; doi:10.1186/s12885-024-12529-7)
Supplement: Supplementary file 2 — Supplementary Material 2. [file 12885_2024_12529_MOESM2_ESM.pdf]

Update #02

ALLIANCE FOR CLINICAL TRIALS IN ONCOLOGY

PROTOCOL UPDATE TO ALLIANCE A022104

THE JANUS RECTAL CANCER TRIAL: A RANDOMIZED PHASE II/III TRIAL TESTING THE EFFICACY OF TRIPLET VERSUS DOUBLET CHEMOTHERAPY REGARDING CLINICAL COMPLETE RESPONSE AND DISEASE-FREE SURVIVAL IN PATIENTS WITH LOCALLY ADVANCED RECTAL CANCER

Commercial agent(s): Oxaliplatin (NSC #266046); 5-Fluorouracil (NSC #19893); Leucovorin calcium (NSC #3590); Capecitabine (NSC #712807); Irinotecan (NSC #616348)
IND Exempt

X	**Update:**		**Status Change:**
	Eligibility changes		Activation
X	Therapy / Dose Modifications / Study Calendar changes		Closure
X	Informed Consent changes		Suspension / temporary closure
X	Scientific / Statistical Considerations changes		Reactivation
X	Data Submission / Forms changes		
X	Editorial / Administrative changes		
X	Other: Updated CTSU language		

No recommended IRB level of review is provided by the Alliance since the CIRB is the IRB of record for this trial.
The site has 30 days after the posting of this amendment to implement it at their site. Please refer to the amendment application and CIRB guidelines for further instructions.

Reconsent is required by Alliance for all populations of the study.

UPDATES TO THE PROTOCOL:

Cover Page (p. 1)

- The study title has been updated to reflect the change from a Phase II to a Phase II/III as follows: The Janus Rectal Cancer Trial: A randomized phase II/III trial testing the efficacy of triplet versus doublet chemotherapy regarding ~~to achieve~~ clinical complete response and disease-free survival in patients with locally advanced rectal cancer.

Study Resources (p. 2)

- Lisa Kottschade has replaced Barbara Kleiber as the Nursing Contact.
- The Pharmacy contact's telephone number has been removed.

Cover Page for Data Submission (p.3)

- The following information has been removed from the Data Submission column: ~~Do not submit study data or forms to the CTSU. Do not copy the CTSU on data submission.~~

Schema (P. 4)

- The title on the schema page has been updated to reflect the change from a Phase II to a Phase II/III.
- Study schema has been update to reflect that patients will be followed for 5 ~~8~~ years.

Section 1.0 (Background)

- In section 1.1 (**Total Neoadjuvant Therapy (TNT) Strategy**), the 8th paragraph has been updated with more background information as follows: 'Importantly, the 7-year updated data from PRODIGE23 presented at ASCO 2023 demonstrated significantly better DFS, metastasis-free survival and overall survival in the TNT-arm versus the standard of care arm (68% vs. 63% DFS). These data support the use of a triplet TNT approach to improve DFS outcome for rectal cancer patients.'
- In section 1.4, (**Rationale for use of the consolidation TNT approach for the Janus Trial**), the second paragraph has been updated with more data as follows: 'Of note, 5-year data in the OPRA trial has since resulted and demonstrated stable organ preservation rates for the CNCT arm (54%) versus 39% in the INCT arm (p=0.012). Further, local regrowth rates remained lower in the CNCT arm (29%) versus 44% in the INCT arm (p=0.02).'

Section 2.0 (Objectives)

- In section 2.1 (**Primary Objectives**), a second paragraph has been added to reflect the studies expansion to a Phase II/III: 'Phase III: To evaluate and compare disease-free survival (DFS) in patients with locally advanced rectal cancer treated with neoadjuvant LCRT followed by neoadjuvant mFOLFIRINOX versus neoadjuvant LCRT followed by neoadjuvant mFOLFOX6.'
- In section 2.2 (**Secondary Objectives**), 2.2.2 has been deleted: ~~To evaluate and compare the disease-free survival (DFS) time between the two treatment arms.~~ and the other objectives have been renumbered.

Section 4.0 (Patient Registration)

- CTSU template language has been updated as required.

Section 5.0 (Study Calendar)

- **Arm 1 and Arm 2 Study Calendars**
  - A header has been added to the Arm 1 and Arm 2 study calendars.
  - a second sentence was added to Footnote *: 'As such, exact week number from registration may vary and as such can be adjusted to reflect timing of start of chemotherapy.'
  - the study week has been update from ~~week 30-36~~ to weeks '32-38.'
  - the following tests and procedures have been added 'History, physical, height and adverse event assessments' In addition, a new footnote ** has been added to define what has to be collected during the history and physical assessment as follows: 'Weight, Pulse, BP, Performance Status. Performance Status will only be required at pre, week 10 and weeks 32-38' and the old footnote ** is now footnote ***.
  - the definition of DRE (digital rectal exam) has been added for clarity.

- footnote **** has been added: 'For women of childbearing potential (see Section 3.2.3). Must be done ≤ 7 days prior to registration.'
- Footnote 3 has been revised to clarify the Med Onc visit: 'Patients will be seen and evaluated during neoadjuvant chemoradiation and chemotherapy with necessary laboratory evaluations per institutional guidelines. For neoadjuvant chemotherapy, recommendation is to be evaluated by medical oncology during (Arm 1) and (Arm 2) chemotherapy every two weeks (every three weeks for patients getting CAPOX) or as needed per institutional guidelines. In patients not seen and evaluated by medical oncology every cycle, adverse events must still be collected and reported every cycle by relevant research staff.' ~~Patients will be seen and evaluated by medical oncology during (Arm 1) and (Arm 2) chemotherapy every two weeks (every three weeks for patients getting CAPOX) or as needed per institutional guidelines. In patients not seen and evaluated by medical oncology every cycle, adverse events must still be collected and reported every cycle by relevant research staff.~~
- ~~(~~**Study Week 10**) footnote #7 has been added for clarification: 'Assessment by surgeon, medical and radiation oncology occurs in a multidisciplinary fashion to ensure the patient has tolerated LCRT well and does not have to occur exactly at week 10; simply should occur prior to initiation of chemotherapy and is meant to ensure appropriate transition to the medical oncology team as they begin systemic chemotherapy.'

- The study calendars for **Evaluations during follow-up for WW** patients and **Evaluations during follow-up for TME patients** have been separated for ease of use for study sites.
- In the WW study calendar (**Footnote ****) has been changed to reflect that MRIs conducted during years 4 through 5 will only be covered if clinically indicated.
- In the WW study calendar, (**Footnote 2**) the second sentence has been deleted as follows: After 36 months, CEA will be evaluated every 6 months up to five years, based on NCCN guidelines. ~~One additional 10 ml EDTA tube of blood will be collected at these time points for research purposes.~~

Section 6.0 (Data and Specimen Submission)

- In section 6.1 the CTSU template language has been updated as required.

Section 7.0 (Treatment Plan/Intervention)

- In section 7.1 (**Neoadjuvant Chemoradiotherapy**)
  - new first and second sentences have been added as follows: 'For specific questions or concerns regarding this section, please contact Dr. William A. Hall- whall@mcw.edu and/or Dr. Paul B. Romesser- romessep@mskcc.org,' and 'Protocol treatment with long course chemoradiation therapy should be initiated within 28 days following protocol registration.'
  - the first sentence of the third paragraph has been revised as follows: During radiation, a fluoropyrimidine must be administered concomitantly (preferred capecitabine 1650 mg/m2/d PO in two divided doses, 'rounded as appropriately per institutional guidelines'…
  - a new second, third and fourth sentence has been added to the end of the third paragraph: 'If in the opinion of the investigator, a toxicity is considered to be due solely to the radiation, then the radiation can be held at the investigator's discretion. The rationale for treatment break and the duration of the break needs to be documented. Investigators are encouraged to reach out to Dr. William A. Hall and/or Dr. Paul B. Romesser to discuss concerns with radiation toxicity resulting in a treatment break.'
  - the first sentence of the fourth paragraph has been revised: Note: Intensity modulated radiation therapy (IMRT) is permitted ~~If treatment includes the inguinal lymph nodes, IMRT is recommended.~~

- In section 7.1 **(Clinical Target Volume)** the fourth sentence has been revised: If the external iliac nodes or inguinal nodes are felt to be at risk for 'micrometastases' ~~involvement~~ by the radiation…and a new fifth sentence has been added" 'If treating the inguinal lymph nodes prophylactically IMRT is required.'
- In section 7.1.1**(Radiation Therapy Quality Assurance)** the Protocol Dosimetrist's email address has been hyperlinked.
- In section 7.2 (**Consolidation Neoadjuvant Chemotherapy)**
  - a new first sentence has been added for clarification: 'For specific questions or concerns regarding this section, please contact Dr. Arvind Dasari adasari@mdanderson.org and/or Dr. Hanna Sanoff mailto:hanna_sanoff@med.unc.edu'.
  - the fifth paragraph has been revised as follows: 'Infusion times, concurrent and/or order of drug administration for all chemotherapy in this protocol may be modified per institutional guidelines.' ~~Administer drugs in the order listed, but infusion times for all chemotherapy in this protocol are per institutional guidelines.~~
- Table 7.2.2 (**Treatment regimen for Control Arm (CAPOX for 5 cycles)** footnote b has been updated with the medication instructions from the medication diary: 'Do not double up doses. If a dose is missed for any reason, skip the dose and take the next dose at the normal time.' And footnote C has been updated with instructions regarding medication compliance as follows: 'Sites may provide ~~Use of~~ patients with a ~~patient~~ pill diary (**Appendix III**) to record capecitabine compliance. However, it is NOT required. Non-English-speaking patients should be instructed to self-document their compliance.'
- In section 7.3 (**Surgery**) the sixth sentence of the first paragraph has been revised: … endoscopy/DRE then endoscopy/DRE should take precedence in the decision making 'on whether' to proceed to WW or TME.

Section 8.0 (Dose and Treatment Modifications)

- In section 8.2 (Dose Modifications) and new eighth paragraph has been added: 'For specific questions or concerns regarding this section, please contact Dr. Arvind Dasari adasari@mdanderson.org and/or Dr. Hanna Sanoff- mailto:hanna_sanoff@med.unc.edu. In the event of treatment-related AEs during consolidation chemotherapy that requires treatment hold for >=30 days from the date of the planned cycle, consolidation chemotherapy should be discontinued. If consolidation chemotherapy is discontinued because of delay, patients the subject should be referred for presurgical restaging as detailed in the study calendar in section 5.0,' and a new nineth paragraph has been added: 'In the event of non-treatment related AEs that result in dose delays of 30-60 days and have resolved to grade <= grade 1 by 60 days from the date of the planned cycle, patients may continue with consolidation chemotherapy if deemed in their best interest by the treating oncologist.'
- In table 8.2.3.2 a minor formatting update has been made to the table instructions: Refer to footnote **'a'** for management of anemia.
- In section 8.2.4 (**Treatment Management of Patients on Chemoradiation**) a NOTE has been added after table 8.2.4.2: 'NOTE: If in the opinion of the investigator, a toxicity is considered to be due solely to the radiation, then the radiation can be held at the investigator's discretion. The rationale for treatment break and the duration of the break needs to be documented. Investigators are encouraged to reach out to Dr. William A. Hall and/or Dr. Paul B. Romesser to discuss concerns with radiation toxicity resulting in a treatment break.'

Section 10.4 (Leucovorin calcium)

- In the third paragraph of the Drug Interactions section, the drug name has been updated as follows: 'Phenobarbital' ~~PHENobarbital:~~ Leucovorin Calcium-Levoleucovorin may decrease the serum concentration of 'Phenobarbital' ~~PHENobarbital~~

Section 11.0 (Measurement of Effect)

- In section 11.2.1 (Clinical and Radiologic Assessment) the link to the endoscopic and MRI educational video has been added: 'Please see CTSU website for endoscopic and MRI assessment of response video by Drs. Smith and Horvat. Please contact Drs. Horvat and/or Smith with questions about this section (see face page for contact information). Alliance A022104 (JANUS) Trial Webinar - October 2023 (youtube.com).'
- In section 11.2, (Clinical Tumor Evaluation prior to TME), the second sentence in the second paragraph has been updated to reflect that patients will be followed for 5 ~~8~~ years.

Section 12.0 (End of Treatment/Intervention)

- In section 12.3.1 (Duration of Follow-up) the table has been completely revised.

Section 13.0 (Statistical Considerations)

- Section 13.0 has been revised to reflect the studies expansion from a Phase II to a Phase II/III.

Section 16.0 (References)

- References 44-48 have been added to reflect the studies expansion from a Phase II to a Phase II/III.

UPDATES TO THE MODEL CONSENT:

- The **official study title** has been updated to reflect the change from a Phase II to a Phase II/III as follows: The Janus Rectal Cancer Trial: A randomized phase II/III trial testing the efficacy of triplet versus doublet chemotherapy regarding ~~to achieve~~ clinical complete response and disease-free survival in patients with locally advanced rectal cancer.
- Under '**What will happen if I decide to take part in this study?'** the second and third sentences of the second paragraph have been updated: They will check you about every '3' ~~4~~ months for 2 years… and After that, they will check you about every 6 months for 3 years 'up to 5 years.'
- Under '**What is the purpose of this study**,' the second sentence of the third paragraph has been updated to reflect the change in the overall enrollment number from ~~312~~ to 760.
- Under 'What are the study groups?, the number of participants in each group has been updated from ~~156~~ to 380.
- Under the '**Drug Risks'** the risk list for **capecitabine** has been updated with the most recent version dated October 12, 2023.
- Under '**Drug Risks'** the risk list for **oxaliplatin** has been updated with the most recent version dated December 21, 2022.

Replacement protocol and model consent documents have been issued.

ATTACH TO THE FRONT OF EVERY COPY OF THIS PROTOCOL

ALLIANCE FOR CLINICAL TRIALS IN ONCOLOGY

ALLIANCE A022104

THE JANUS RECTAL CANCER TRIAL: A RANDOMIZED PHASE II/III TRIAL TESTING THE EFFICACY OF TRIPLET VERSUS DOUBLET CHEMOTHERAPY REGARDING CLINICAL COMPLETE RESPONSE AND DISEASE-FREE SURVIVAL IN PATIENTS WITH LOCALLY ADVANCED RECTAL CANCER

Commercial agent(s): Oxaliplatin (NSC #266046); 5-Fluorouracil (NSC #19893); Leucovorin calcium (NSC #3590); Capecitabine (NSC #712807); Irinotecan (NSC #616348)
IND Exempt

ClinicalTrials.gov Identifier: NCT05610163

Study Chair
J. Joshua Smith, MD, PhD
Memorial Sloan Kettering Cancer Center
1275 York Avenue, SR-201
New York, NY 10065
Tel: 212-639-5807 Fax: 929-321-1061
smithj5@mskcc.org

NRG Co-chair and Champion
William Hall, MD
Medical College of Wisconsin
Tel: 414-719-4694
whall@mcw.edu

SWOG Co-chair and Champion
Arvind Dasari, MD
MD Anderson Cancer Center
adasari@mdanderson.org

Surgical Oncology Co-chair
Julio Garcia-Aguilar, MD, PhD
Memorial Sloan Kettering
Tel: 626-471-9309
garciaaj@mskcc.org

Medical Oncology Co-chair
Hanna Sanoff, MD, MPH
University of North Carolina
Tel: 877-668-0683
hanna_sanoff@med.unc.edu

Community Oncology Co-chair
Tareq Al Baghdadi, MD
St. Joe's Health
Tel: 734-712-1000
tareq_albaghdadi@ihacares.com

Correlative Co- chair
Paul Romesser, MD
Memorial Sloan Kettering
Tel: 646-888-2118
romessep@mskcc.org

Alliance GI Committee Co-chairs
Jeffrey Meyerhardt, MD, MPH
Dana Farber Cancer Institute
Tel: 617-632-6855
jeffrey_meyerhardt@dfci.harvard.edu

Eileen O'Reilly, MD
Memorial Sloan Kettering
Tel: 646-888-4182
oreillye@mskcc.org

ECOG Champion
Olatunji Alese, MD
Emory University
Tel: 404-778-1900
Olatunji.alese@emory.edu

Radiology Co-chair
Natally Horvat, MD, PhD
Memorial Sloan Kettering
Tel: 646-608-8008
horvatn@mskcc.org

SWOG GI Committee Chair
Philip Philip, MD
Wayne State University
ad6613@wayne.edu

NRG GI Committee Chair
Thom George, MD
University of Florida
thom.george@medicine.ufl.edu

Primary Statistician
Qian Shi, PhD
Tel: 507-538-4340
shi.qian2@mayo.edu

Secondary Statistician
Garth D. Nelson, MS
Tel: 507-266-9483
nelson.garth@mayo.edu

Protocol Coordinator
Jamie Crawley
Tel: 773-702-9934
jcrawley@bsd.uchicago.edu

Data Manager
Kelsey Peterson
Tel: 507-538-1700
peterson.kelsey1@mayo.edu

Participating Organizations:
Alliance/Alliance for Clinical Trials in Oncology (lead), **ECOG-ACRIN**/ECOG-ACRIN Cancer Research Group, **NRG**/NRG Oncology, **SWOG**/SWOG

Study Resources:

**Expedited Adverse Event Reporting**<br>https://ctepcore.nci.nih.gov/ctepaers	**Medidata Rave® iMedidata portal**<br>https://login.imedidata.com
**OPEN (Oncology Patient Enrollment Network)**<br>https://open.ctsu.org	**Biospecimen Management System**<br>http://bioms.allianceforclinicaltrialsinoncology.org

Protocol Contacts:

A022104 Nursing Contact
Lisa Kottschade, APRN, MSN, CNP, FAPO
Mayo Clinic
kottschade.lisa@mayo.edu

A022104 Pharmacy Contact
Maria Andrea Monckeberg, MS, RPh, BCOP
Rhode Island Hospital
mamonckeberg@lifespan.org

Alliance Biorepository at Washington University
BJC Institute of Health
425 S. Euclid Avenue, Rm 5120
St. Louis, MO 63110
Tel: 314-747-4402
alliance@email.wustl.edu

IROC
For Imaging: IROC Ohio,
alliance022104@irocohio.org or 614-293-2929
For RT QA: IROC Rhode Island,
IROCRI@QARC.org or 401-753-7600
For RT Credentialing: IROC Houston,
irochouston@mdanderson.org, or 713-745-8989

Protocol-related questions may be directed as follows:

**Questions**	**Contact (via email)**
Questions regarding patient eligibility, treatment, and dose modification:	Study Chair, Nursing Contact, Protocol Coordinator, and (where applicable) Data Manager
Questions related to data submission, RAVE or patient follow-up:	Data Manager
Questions regarding the protocol document and model informed consent:	Protocol Coordinator
Questions related to IRB review	Alliance Regulatory Inbox<br>regulatory@allianceNCTN.org
Questions regarding CTEP-AERS reporting:	Alliance Pharmacovigilance Inbox<br>pharmacovigilance@alliancenctn.org
Questions regarding specimens/specimen submissions:	Alliance Biorepository
Questions regarding drug administration	Pharmacy Contact

CANCER TRIALS SUPPORT UNIT (CTSU) ADDRESS AND CONTACT INFORMATION

**For regulatory requirements:**	**For patient enrollments:**	**For data submission:**
Regulatory documentation must be submitted to the Cancer Trials Support Unit (CTSU) via the Regulatory Submission Portal.<br><br>(Sign in at https://www.ctsu.org, and select the Regulatory > Regulatory Submission.)<br><br>Institutions with patients waiting that are unable to use the Portal should alert the CTSU Regulatory Office immediately by phone or email: 1-866-651-CTSU (2878), or CTSURegHelp@coccg.org to receive further instruction and support.<br><br>Contact the CTSU Regulatory Help Desk at 1-866-651-CTSU (2878) or CTSURegHelp@coccg.org for regulatory assistance.	Refer to the patient enrollment section of the protocol for instructions on using the Oncology Patient Enrollment Network (OPEN). OPEN is accessed at https://www.ctsu.org/OPEN_SYSTEM/ or https://OPEN.ctsu.org.<br><br>Contact the CTSU Help Desk with any OPEN-related questions by phone or email : 1-888-823-5923, or ctsucontact@westat.com.	Data collection for this study will be done exclusively through Medidata Rave. Refer to the data submission section of the protocol for further instructions.
The most current version of the **study protocol and all supporting documents** must be downloaded from the protocol-specific page located on the CTSU members' website (https://www.ctsu.org).<br><br>Permission to view and download this protocol and its supporting documents is restricted and is based on person and site roster assignment housed in the Roster Maintenance application and in most cases viewable and manageable via the Roster Update Management System (RUMS) on the CTSU members' website.		
**For clinical questions (i.e., patient eligibility or treatment-related)** see Protocol Contacts, Page 2.		
**For non-clinical questions (i.e., unrelated to patient eligibility, treatment, or clinical data submission)** contact the CTSU Help Desk by phone or email:<br><br>CTSU General Information Line – 1-888-823-5923, or ctsucontact@westat.com. All calls and correspondence will be triaged to the appropriate CTSU representative.		

THE JANUS RECTAL CANCER TRIAL: A RANDOMIZED PHASE II/III TRIAL TESTING THE EFFICACY OF TRIPLET VERSUS DOUBLET CHEMOTHERAPY REGARDING CLINICAL COMPLETE RESPONSE AND DISEASE-FREE SURVIVAL IN PATIENTS WITH LOCALLY ADVANCED RECTAL CANCER

Eligibility Criteria (see Section 3.2)

- Clinical stage II or III rectal adenocarcinoma defined as T4N0, or any T with node positive disease (any T, N+); also T3N0 requiring APR or coloanal anastomosis
- No prior systemic chemotherapy, targeted therapy, or immunotherapy; or radiation therapy administered as treatment for colorectal cancer within the past 5 years
- Not pregnant and not nursing
- Age ≥ 18 years
- ECOG Performance Status 0-1
- No upper rectal tumors (distal margin of tumor > 12 cm from the anal verge)
- No recurrent rectal cancer; prior transanal excision, prior distal sigmoid cancer with a low anastomosis
- No known mismatch repair deficient rectal adenocarcinoma

Required Initial Laboratory Values

ANC	≥ 1500/mm^3
Platelet count:	≥ 100,000/mm^3
Creatinine:	≤ 1.5 x upper limit of normal (ULN) OR
Calc. creatinine clearance:	≥ 50 mL/min
Total bilirubin:	≤ 1.5 x ULN
AST/ALT:	≤ 3 x ULN

Schema

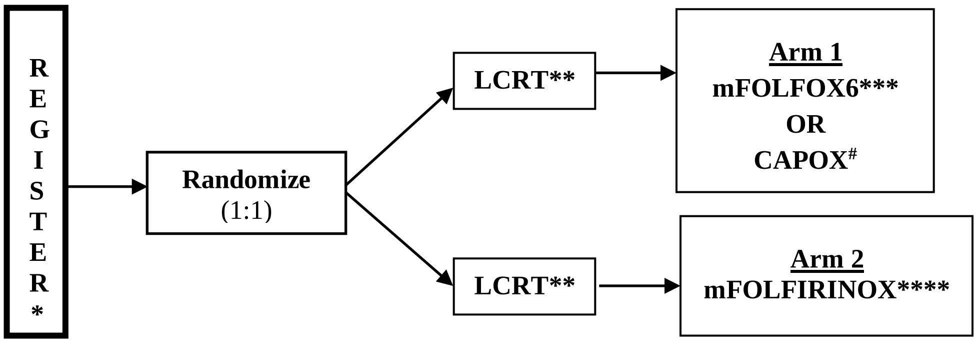

* Patients with locally advanced rectal cancer: <=12cm, T4N0 OR anyT, N+ OR T3N0 that would require APR or coloanal anastomosis
** LCRT = long-course chemoradiation (5 weeks)
***mFOLFOX6 = 8 cycles (1 cycle = 2 weeks)
****mFOLFIRINOX = 8 cycles (1 cycle = 2 weeks)
CAPOX = 5 cycles (1 cycle = 3 weeks)

Treatment is to continue for the full course of LCRT and Arm 1 or Arm 2 chemotherapy unless there is a clinical reason to stop. Following neoadjuvant chemotherapy, patients will either proceed to surgery (TME) or watch & wait (WW). Patients will be followed for 5 years or until death, whichever comes first.

Please refer to the full protocol text for a complete description of the eligibility criteria and treatment plan.

If the Group credited for enrollment is a non-Alliance Group, then other requirements from the credited Group may apply.

Table of Contents

Section Page

1.0 BACKGROUND ... 7
1.1 Total Neoadjuvant Therapy (TNT) Strategy ... 7
1.2 Watch & Wait (WW) Rationale ... 10
1.3 The Janus Rectal Cancer Trial ... 11
1.4 Rationale for use of the consolidation TNT approach for the Janus Trial ... 12

2.0 OBJECTIVES ... 14
2.1 Primary objective ... 14
2.2 Secondary objective(s) ... 14
2.3 Exploratory objective ... 14

3.0 PATIENT SELECTION ... 15
3.1 On-Study Guidelines ... 15
3.2 Eligibility Criteria ... 15

4.0 PATIENT REGISTRATION ... 17
4.1 Investigator and Research Associate registration with CTEP ... 17
4.2 Cancer Trials Support Unit registration procedures ... 18
4.3 Patient Registration Requirements ... 20
4.4 Patient registration/randomization procedures (Step 1) ... 21
4.5 Stratification Factors and Treatment Assignments ... 21

5.0 STUDY CALENDAR ... 23

6.0 DATA AND SPECIMEN SUBMISSION ... 26
6.1 Data Collection and Submission ... 26
6.2 Specimen collection and submission ... 28
6.3 Digital radiation therapy data submission using Transfer of Images and Data (TRIAD) ... 28

7.0 TREATMENT PLAN/INTERVENTION ... 29
7.1 Neoadjuvant Chemoradiotherapy ... 30
7.2 Consolidation Neoadjuvant Chemotherapy ... 35
7.3 Surgery ... 37
7.4 Imaging ... 37

8.0 DOSE AND TREATMENT MODIFICATIONS ... 37
8.1 Ancillary Therapy, Concomitant Medications, and Supportive Care ... 37
8.2 Dose Modifications ... 40

9.0 ADVERSE EVENTS ... 52
9.1 Routine Adverse Event Reporting ... 52
9.2 CTCAE Routine Reporting Requirements ... 53
9.3 Expedited Adverse Event Reporting (CTEP-AERS) ... 53

10.0 DRUG INFORMATION ... 56
10.1 General Considerations: ... 56
10.2 Oxaliplatin (NSC #266046) ... 57
10.3 5-Fluorouracil (NSC# 19893) ... 61
10.4 Leucovorin calcium (NSC# 3590) ... 63
10.5 Capecitabine (NSC #712807) ... 65
10.6 Irinotecan (NSC# 616348) ... 67

11.0 MEASUREMENT OF EFFECT ... 70

11.1 Radiological Tumor Evaluation....70
11.2 Clinical Tumor Evaluation prior to TME....70
11.3 Pathological Tumor Response at TME....72
11.4 Disease Evaluation after TME....73

12.0 END OF TREATMENT/INTERVENTION....74

12.1 Duration of Protocol Treatment....74
12.2 Criteria for Discontinuation of Protocol Treatment/Intervention....74
12.3 Follow-up....75
12.4 Extraordinary Medical Circumstances....76
12.5 Managing ineligible patients and registered patients who never receive protocol intervention..76

13.0 STATISTICAL CONSIDERATIONS....76

13.1 Study Endpoints....76
13.2 Sample Size....77
13.3 Power Justification....77
13.4 Statistical Analysis Plan....79
13.5 Monitoring....80
13.6 Inclusion of Women and Minorities....83

14.0 CORRELATIVE AND COMPANION STUDIES....84

14.1 Correlative Science....84

15.0 MONITORING PLAN....85

16.0 REFERENCES....86

APPENDIX I: RECOMMENDED CLINICAL MANAGEMENT OF DIARRHEA....90

APPENDIX II: LEVOLEUCOVORIN DRUG DOSE AND ADMINISTRATION INSTRUCTIONS....91

APPENDIX III PATIENT MEDICATION DIARIES....92

1.0 BACKGROUND

1.1 Total Neoadjuvant Therapy (TNT) Strategy

TNT Strategy: Patients accrued to this study will be treated with either LCRT followed by consolidation FOLFOX (control arm), as this has been associated with the highest rate of organ preservation in the OPRA trial[1] or LCRT followed by consolidated chemotherapy with mFOLFIRINOX (experimental arm) as this approach using triplet versus doublet regimens has been demonstrated to improve response rates in both the primary and metastatic rectal and colorectal setting ([2], [3]. We have designed the Janus trial to expand on the findings of the OPRA trial, and to further examine a TNT method utilized to improve DFS outcomes and drive up response rates using a chemotherapy intensification approach. Of note, we queried two separate patient advocate groups (Colontown and Fight Colorectal Cancer) on whether they would prefer a chemoescalation approach versus a radiation escalation approach (anonymous online surveys, with permission). The patients indicated by an 80% to 20% margin, a strong preference for a chemoescalation approach (Smith JJ, George M, Garcia R, et al. March 2021, unpublished). This chemoescalation approach has been further discussed at the Spring (2021) SWOG, NRG, Alliance, the most recent study team meetings, and the most recent NCI RATF meeting and has been approved by all groups to move forward.

- Consolidation chemotherapy (CNCT) as the control arm: CNCT (LCRT then chemotherapy) has been thought to potentially delay treatment of micrometastases for several weeks compared to induction chemotherapy, but it did not seem to interfere with LCRT in either the OPRA trial[1] or the recent German trial[4]. Furthermore, we observed no differences in DFS, organ preserved DFS, or local regrowth-free distant metastasis-free survival in OPRA compared to induction chemotherapy followed by LCRT[1]. A CNCT approach has been reported in anal cancer in the ACT II trial (UK[5]) and in LARC in the TIMING (US[6]) and CAO/ARO/AIO-12 (GER[4]) trials. It has been well tolerated with no untoward effects on response to treatment[1], [4], [6]. The TIMING (*Timing of Rectal Cancer Response to Chemoradiation*) trial reported pCR rates of 38%, the highest to date, in patients with LARC who received 6 cycles of FOLFOX following CRT[6]. Additional evidence for the efficacy of this approach has been shown in the recent German trial with acceptable pCR and superior CR rates (25% pCR vs 17% pCR) compared to an induction chemotherapy approach[4].

- Consolidation chemotherapy with mFOLFIRINOX as the experimental arm:
  5-FU, Irinotecan and Oxaliplatin in Advanced Colorectal Cancer: Chemotherapy regimens combining fluoropyrimidine (5-FU / capecitabine), oxaliplatin and/or irinotecan form the mainstay of systemic treatment of colorectal cancer. Multiple phase II and phase III clinical trials have compared doublets (fluoropyrimidine with oxaliplatin or irinotecan) with the triplet regimen (fluoropyrimidine with oxaliplatin and irinotecan) to consistently improve outcomes including objective radiographic response rates (ORR), overall survival (OS) and progression free survival (PFS) [2], [7]–[13]. Based on these results, the triplet regimen is included among first-line options in most clinical guidelines and recommendations worldwide [14]–[16]. A recent individual patient data meta-analysis of triplet versus doublets as the initial therapy of advanced unresectable metastatic colorectal cancer pooled data from 5 such trials [2]. The primary end point of the study was OS and key secondary end points included PFS, ORR and R0 resection rate. A total of 1,697 patients were included in this analysis and after a median follow-up of 39.9 months, patients assigned to the triplet regimen had significantly longer OS than those assigned to doublets (median, 28.9 *v* 24.5 months; hazard ratio [HR], 0.81; 95% CI, 0.72 to 0.91; *P*< .001). A total of 1,489 (88%) patients experienced first-line disease progression and median PFS was 12.2 months (95% CI, 11.6 to 12.8 months) in the triplet group and 9.9 months

(95% CI, 9.5 to 10.3 months) in the doublets group (HR, 0.74; 95% CI, 0.67 to 0.82; *P* < .001). The estimated 5-year OS rate was 22.3% (95% CI, 18.0% to 26.6%) in the triplet group and 10.7% (95% CI, 6.6% to 14.8%) in the doublets group (*P* < .001). Among 1,695 of the patients evaluable for RECIST, 545 (64.5%) of 845 in the triplet group and 456 (53.6%) of 850 in the doublet group achieved an objective response (odds ratio [OR], 1.57; 95% CI, 1.29 to 1.91; *P* < .001). Together, these data convincingly show *increased efficacy* of triplet over doublet chemotherapy in patients with advanced colorectal cancer in improving ORR, PFS and OS.

5-FU, Irinotecan and Oxaliplatin in Resectable Colorectal Cancer: Although the impact of conversion to resectability of metastatic disease and subsequent relapse free survival (RFS) were not primary endpoints of most of these trials, data suggest trends towards improvement in both outcomes with triplet vs doublet regimen. The OLIVIA (ClinicalTrials.gov identifier:NCT00778102) trial was a multinational open-label phase II study that randomized 80 colorectal cancer patients with unresectable liver metastases to doublet vs triplet based chemotherapy. The overall resection rate for first resections, the primary end point, was 61% (95% CI 45% to 76%) in the triplet group and 49% (95% CI 32% to 65%) in the doublet (difference 12%; 95% CI –11% to +36%). The final R0 resection rates were 54% (95% CI 37% to 69%) with triplet and 31% (95% CI 17% to 48%) with doublet regimens respectively [10]. Intriguingly, median RFS, which was assessed in patients with an R0/R1 status after first resection, was 17.1 months (95% CI 12.3 months to not reached) with triplet regimen and 8.1 (95% CI 3.8–11.7) months with doublet (hazard ratio 0.31; 95% CI 0.12–0.75), suggesting an improvement in RFS in patients with resectable disease with the triplet regimen. Similar trends were noted in the above-mentioned pooled analysis with R0 resection being performed in 16.4% of patients in the triplet group and 11.8% patients in the doublet group (OR, 1.48; 95% CI, 1.12 to 1.95; *P*= .007). In patients with R0 resection, although RFS was not reported, median OS was 64.0 months with the triplet and 52.6 months with doublets (HR, 0.79; 95% CI, 0.50 to 1.24). These data together strongly suggest improvement in RFS and OS with the triplet regimen over fluoropyrimidine + oxaliplatin in patients with resectable metastatic CRC. These data have provided adequate credence to design large trials evaluating the role of the triplet regimen in adjuvant therapy of high-risk colon cancer towards improving disease free / relapse free survival over the doublet of fluoropyrimidine and oxaliplatin. The ongoing CCTG trial CO.27 (EudraCT number: 2016-001491-29) is a phase III randomized trial comparing FOLFIRINOX triplet chemotherapy to FOLFOX in high-risk stage III colon cancer in the adjuvant setting. The recently approved phase II / III NRG GI008 proposes to compare doublet chemotherapy with fluoropyrimidine and oxaliplatin to triplet chemotherapy in stage III colon cancer patients with ctDNA defined minimal residual disease (ClinicalTrials.gov identifier pending).

5-FU, Irinotecan and Oxaliplatin in Rectal Cancer: The FFCD 1102 study was a multicenter non-randomized phase II trial that enrolled 65 rectal cancer patients with unresectable synchronous metastases[3]. Patients received induction chemotherapy with FOLFIRINOX for up to 8 cycles with the aim of evaluating the efficacy of induction chemotherapy with FOLFIRINOX in these patients. Central review of CT scans and rectal magnetic resonance imaging (MRI) was planned in the protocol and performed blinded to the clinical data and to the tumor response as assessed by investigators. Tumor response according to RECIST criteria was assessed using CT scans, and response of rectal tumors was assessed by MRI, at baseline, after 4 and 8 cycles of FOLFIRINOX. A partial response was defined as a tumor volume reduction of at least 70%. The 4-month disease control(DC) rate was 91% (95%CI, 82.3% - 95.8%) according to the investigators and 94% (95% CI, 86.3 - 97.8) according to the review committee, as compared to the planned alternative

hypothesis of a 4-month DC rate of >75%. Partial response was noted in 47% of patients after 4 cycles and in 86% of patients after 8 cycles in metastatic sites according to central independent review. Response in primary rectal tumors was noted in 49% after 4 cycles and in 63% after 8 cycles. At inclusion, 47 patients (72%) had local symptoms: rectal bleeding (n = 27, 42%) and/or rectal syndrome (n = 39, 60%). Local symptoms, rectal bleeding and/or rectal syndrome were seen in 16%, 5% and 14% of patients after 4 cycles of FOLFIRINOX, and in 10%, 3% and 7%, after 8 cycles, respectively. Similarly, another phase II study, GRECCAR 4 reported an 80% ORR (more than 50% size reduction of tumor) of patients with locally advanced rectal cancer after four cycles of FOLFIRINOX[17]. Together, these studies demonstrate *substantial tumor regression* with FOLFIRINOX in rectal cancer patients.

More recently, the PRODIGE23 trial enrolled 460 patients with locally advanced rectal cancer (cT3-4 cN0-N+) to LCRT followed by surgery and adjuvant FOLFOX (control, CRT arm) vs induction mFOLFIRINOX followed by LCRT, surgery and adjuvant FOLFOX (experimental, TNT arm)[18]. Approximately 63% had tumors 5 cm or beyond from the anal verge, 80% had cT3 tumors, 90% had node positive disease and 27% had threatened circumferential margins. The study met its primary endpoint of improvement in 3-year DFS (75.7%, 95% CI 69.4 – 80.8 vs 68.5%, 95% CI 61.9 – 74.2%; HR 0.69; p = 0.034) along with improvement in pathological response (pCR 27.8% vs 12.1%; p < 0.001). Cumulatively, these data provide support for a TNT approach including FOLFIRINOX to improve response rates in patients with LARC. Building on these data and the data from OPRA, we anticipate that employing FOLFIRINOX in a consolidation approach after LCRT has the potential to further drive response rates higher (increasing cCR rates) with an associated increase in organ preservation rates. Notably, FOLFIRINOX continues to be actively studied in Europe. In fact, the ongoing phase III NORAD01-GRECCAR16 trial (ClinicalTrials.gov identifier NCT03875781) is randomizing 551 patients with T3N0 or T1-3N+ rectal adenocarcinoma with CRM > 2 mm (e.g.. patients with relatively low risk rectal cancer) utilizing a non-inferiority design to modified FOLFIRINOX (mFOLFIRINOX) vs chemoradiation followed by surgery and adjuvant therapy per physicians' discretion. The primary endpoint is 3-year DFS utilizing a non-inferiority design. The rationale for NORAD01-GRECCAR16 is to demonstrate non-inferiority of preoperative mFOLFIRINOX alone compared to CRT in early stage locally advanced resectable rectal cancer on oncologic outcomes towards avoiding late side effects associated with pelvic irradiation.

5-FU, Irinotecan and Oxaliplatin Dosing: The initial regimens combining these three drugs involved a higher dose of 5-FU at 3,200 mg/m^2 continuous infusion over 48 hours, leucovorin 400 mg/m^2, irinotecan 150 mg/m^2 and oxaliplatin 85 mg/m^2 on day 1 (FOLFOXIRI). However, the dose of 5-FU used in these European studies is poorly tolerated in U.S. patients and the dosing for the regimen will be modified based on the NCCN guidelines as follows (mFOLFIRINOX):

**Drug**	**Dose**
5-Flurouracil	2400 mg/m^2 continuous iv infusion over 46-48 hours
Leucovorin	400 mg/m^2 iv day 1
Irinotecan	150 mg/m^2 iv day 1
Oxaliplatin	85 mg/m^2 iv day 1
	Repeat every 2 weeks

In the PRODIGE23 trial (that used doses as detailed above except for a slightly higher dose of irinotecan at 180 mg/m^2 iv day 1), although slightly higher rates of ≥ grade 3 neutropenia (2.8% vs 0) and capecitabine discontinuation (8% vs 3%; $p < 0.02$) were noted during LCRT in the TNT arm, they did not impact proportion of patients undergoing subsequent surgery (92.2% vs 94.8%; $p = 0.26$) perioperative morbidity or quality of life. In fact, trends towards improvement in non-therapeutic surgery (0% vs 3.7%); metastatic disease at pre-surgical staging (1% vs 4.7%) and better tolerance of subsequent adjuvant therapy (≥ grade 3 toxicities 11.7% vs 20.7%) were noted favoring the TNT arm. Importantly, the 7-year updated data from PRODIGE23 presented at ASCO 2023 demonstrated significantly better DFS, metastasis-free survival and overall survival in the TNT-arm versus the standard of care arm (68% vs. 63% DFS)[44]. These data support the use of a triplet TNT approach to improve DFS outcome for rectal cancer patients.

1.2 Watch & Wait (WW) Rationale

WW Rationale: The treatment of patients with non-metastatic locally advanced rectal cancer (LARC) includes pre-operative chemoradiation (CRT), TME, and post-operative adjuvant chemotherapy[19]. This trimodal treatment provides excellent local tumor control and survival, but each modality is associated with long-term AEs that permanently impair QOL. The challenge is to identify treatment approaches that could maintain or improve oncologic outcomes while preserving QOL[20]. Some patients with LARC have a pCR after CRT. Patients with a pCR have lower tumor recurrence and improved survival compared to patients without a pCR, and thus raising questions about the added value of TME for these individuals[20]–[22]. As most of the morbidity from trimodal therapy is related to TME, selective avoidance of TME in patients who obtain a CR to CRT could reduce overtreatment and improve QOL. Patients participating in this trial could therefore benefit from organ preservation and improvement in QOL. However, identifying patients with a pCR without removing the rectum is difficult, and a selective WW strategy for patients with an apparent cCR after neoadjuvant therapy (NAT) carries risks of tumor regrowth and distant metastases. Thus, with significant input from the thought leaders in Alliance, NRG, SWOG, NCI, CTEP and the GISC in addition to critical patient input, we designed the current trial of chemotherapy escalation to maximize cCR using a modern design that builds on recent randomized trial data in locally advanced rectal cancer which allows patients who have excellent responses (cCR) a WW option (NOT mandated) whereas, those with incomplete responses will be managed with traditional surgery.

Retrospective institutional case series suggest that a WW strategy is feasible and well accepted by patients with rectal cancer who achieve a cCR[23]–[25]. Prospective data from the OPRA trial now shows that incorporating WW for those achieving a cCR using a TNT strategy is not associated with worse DFS outcomes than what has been observed in historical control patients treated with CRT and then TME[1]. Now, we design a trial employing an optimal TNT approach expanding on the findings of OPRA and by incorporating patient input to drive up responses by using a chemotherapy intensification approach to increase cCR compared to a standard consolidation TNT approach. We will also examine the 3-year organ preservation rates between the two arms along with DFS and OS outcomes.

WW Challenges: While optimal in design, a phase II or III trial randomizing patients with a cCR to either WW or TME after NAT completion is not feasible. In working with **national colorectal cancer patient advocates**, our study leadership team has established that patients with a cCR to NAT **will not accept randomization between WW and TME**. Clinicians have mixed opinions regarding WW, some supporting its adoption, others strongly opposing WW. Many rectal cancer patients have expressed a preference for preserving the rectum after achieving a CR to neoadjuvant chemoradiation. However, a significant proportion of them wonder whether WW is safe in the long-term. We have designed the current trial of

chemotherapy intensification using patient and provider input with the intent to *increase cCR rates and drive up organ preservation rates* and thus *improve quality of life* for patients with rectal cancer. Therefore, in this study, we propose a nationwide trial of optimal TNT using a consolidation approach of 3-drugs (mFOLFIRINOX) versus 2-drugs (FOLFOX) based on the hypothesis that the 3-drug approach after LCRT will demonstrate superior cCR rates compared to the 2-drug approach after LCRT. The OPRA trial showed an organ preservation rate at 3 years post-TNT of 53% for the consolidation arm (CRT then chemotherapy) and 41% for the induction arm (chemotherapy then CRT) with no significant differences in 3-year DFS between the arms[1]. To maintain consistency from OPRA to this trial, the determination of cCR will be based on the same regression schema (see Section 11.2) used to determine response in OPRA[1].

1.3 The Janus Rectal Cancer Trial

The Janus trial is important for our rectal cancer patients as a builds on the findings of modern rectal cancer trials[1], [18] to move the field forward relative to validation of the cCR endpoint and to enhance quality of life for patients using a chemotherapy intensification TNT approach. Randomizing patients to either mFOLFOX6 + LCRT or mFOLFIRINOX + LCRT and followed by selective surgery or WW based on tumor response, we seek to: a) increase cCR rates and b) improve the response of the primary tumor. With the current trial design, we aim to better our patient's lives by improving their organ preservation outcomes and by giving more patients the chance for improved QoL. Preserving the rectum is a significant quality of life benefit for those patients who achieve a cCR. Importantly, this trial will provide an opportunity to improve quality of life for our patients, will allow a venue to prospectively validate the schema used in OPRA for assessing tumor response, and allow us to gain critical insight into the biology of response to consolidation TNT approaches in the context of standard clinical measures and novel correlative biomarkers.

A major criticism of previously published WW data is that most of it comes from a select patient population treated at specialized centers. The recently reported OPRA trial was conducted across sixteen highly specialized centers, and thus provides the most robust, prospective data on outcomes of WW[1]. While all OPRA sites were selected based on the expertise and clinical interest of the surgical team, the Janus trial will determine generalizability of a WW approach across a more diverse population of patients, practice sites, and providers while incorporating a chemotherapy intensification approach in the context of modern TNT to improve response outcomes in an agile Phase II trial incorporating the WW strategy in a manner that will be acceptable to patients and clinicians.

Despite the strengths of OPRA, it is a single phase II study with limited statistical power to inform a change in practice. Here we have an opportunity to validate the findings in OPRA for patients achieving a cCR and thus managed with a WW strategy. Importantly, now that OPRA has shown that a WW strategy can be safely incorporated, in this trial, we will endeavor to expand on those findings by using the consolidation arm of OPRA as the control arm of the study and then comparing this to a chemotherapy escalation approach to demonstrate that using mFOLFIRINOX after LCRT is superior relative to cCR rates compared to mFOLFOX after LCRT. In this trial, we will use the same clinical response criteria used in the OPRA trial relative to clinical exam, endoscopy, and MRI findings[1], [26] (see MSK Regression Schema below). Importantly for this national trial, we will provide educational opportunities via webinars prior to starting the trial for the assessment and recognition of cCR by surgeons and radiologists. Further, we have developed an online tool which can be employed for broad access during the trial for use by surgeons and radiologists as a reference point during the course of the trial. Further, we will add in safety and data monitoring for cCR rates and time to TME, as well as rates of local regrowth and distant metastasis. For example, among patients who start a WW strategy after TNT, we will monitor the proportion of patients who develop progression of

disease which would preclude TME. If this rate is high, we will pause the trial to investigate. Also, among patients who start a WW strategy after TNT, then undergo salvage TME, we will monitor the proportion of patients who achieve R0 resection. If this rate is too low, we will pause the trial to investigate data. Additionally, we propose the measurement of ctDNA as an exploratory biomarker and aim to develop a meaningful “minimal residual disease” based risk classification for cCR patients as a potential future tool to help guide patients and clinicians in the difficult shared decision-making process of TME. A well-powered, nationwide trial will help validate the safety and efficacy of organ preservation outside of specialized centers for our patients with rectal cancer; further without a properly powered randomized trial with cCR as the primary endpoint it will be difficult to fully validate the findings of OPRA and make it difficult to formally incorporate a WW approach into standard guidelines for national thought leaders (e.g., NCCN). The trial will compare treatment compliance and AEs between those randomized to the LCRT + mFOLFIRINOX arm to those randomized to the LCRT + mFOLFOX6 arm, and it will identify opportunities for improving delivery of radiotherapy by leveraging the expertise of the NCTN, the Alliance, SWOG, and NRG Oncology.

1.4 Rationale for use of the consolidation TNT approach for the Janus Trial

TNT and the consolidation approach: The use of TNT is now at the forefront for patients with locally advanced rectal cancer[1], [6], [27]–[30]. The TIMING trial investigated the effect of delivering 2, 4, or 6 cycles of CRT then FOLFOX followed by TME to patients with LARC[6]. This study found that adding 2, 4, or 6 cycles of FOLFOX after LCRT increases the pCR rates to 25%, 30%, and 38%, respectively, compared to LCRT alone (18%) without increasing the rate of AEs or complications. Additional evidence for the use of a consolidation approach comes from the recent phase II randomized German trial which compared two TNT approaches (a) induction chemotherapy and b) consolidation chemotherapy) and showed that a consolidation approach was associated with a 25% pCR rate versus a 17% pCR rate with an induction approach[4]. Moreover, the toxicity profile, post-operative morbidity and tolerance of both regimens was similar. Recently, the RAPIDO trial, as an adaptation of the TIMING trial, but employing LCRT concurrent with chemotherapy followed by TME versus SCRT followed by CAPOX or FOLFOX followed by TME, showed that this approach is safe and that this derivative TNT approach (SCRT → chemotherapy then TME) is associated with higher pCR rates compared to LCRT and TME alone (28% vs. 14%), with no decrement in survival outcomes and less distant metastases[30], [31]. These studies demonstrate that a TNT approach increases compliance with systemic chemotherapy and increases rates of tumor response compared to LCRT alone, and further indicate that consolidation approaches further increase complete response rates. Of note, the safety and efficacy of SCRT has not been evaluated in the context of organ preservation and the German trial group (Fokas E and Rodel C et al – personal communication) is investigating this strategy in an onoing trial comparing LCRT vs. SCRT followed by consolidative chemotherapy then WW or TME based on response – thus, we do not plan to allow SCRT in this trial to avoid competing with an ongoing trial in Europe.

Specific rationale for use of the proposed consolidation approach for Janus in the control arm: OPRA showed a higher cCR rate using a consolidation versus an induction approach (55% versus 42%; 3-year organ preservation rates). **Figure 1** provides compelling rationale for use of CRT followed by chemotherapy (CNCT) as the control arm TNT strategy for Janus [1]. Of note, the trials mentioned above using consolidation approaches showed pCR rates ranging from 25% to 38%, whereas OPRA allowed an organ preservation option and demonstrated a cCR rate of 53%. The higher cCR rates could be related to employing a cCR/near Complete Response (nCR) endpoint 8-12 weeks post-TNT to allow organ preservation[26] versus mandating a set endpoint with TME, as has been standard in many prior studies which analyzed pCR rates[6], [31]. nCR patients were given at least 4-8 weeks to evolve to a cCR. If they evolved to a cCR then the

patients were recommended to undergo a WW approach. Patients were monitored carefully as long as the tumor continued to respond and regress to a cCR. If the tumor stopped responding or progressed, the patients were recommended to undergo TME. The criteria for cCR, nCR and

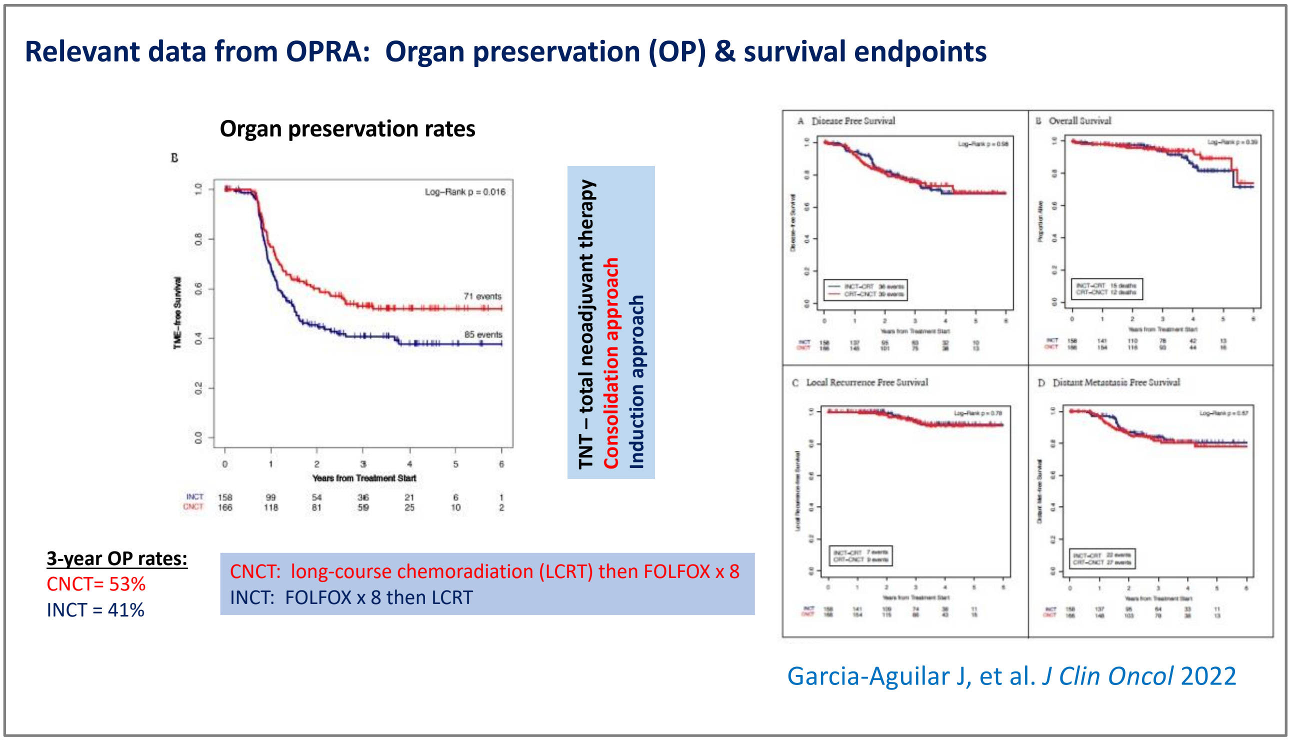

FIGURE 1: Data from the OPRA trial (as presented at ASCO 2020 and now online (Garcia-Aguilar J. et al. *J Clin Oncol*. 2022)) is shown above demonstrating 53% 3-year organ preservation rate for the CNCT group versus 41% for the INCT group. No differences were noted between either TNT arm (INCT or CNCT) for 3-year DFS, overall, local recurrence-free or distant metastasis-free survival. In addition, pathological outcomes for each TNT arm were noted and there was no difference in the rate of pCR between the TNT arms (9% (INCT) vs. 8% (CNCT)). CNCT – consolidation chemotherapy after CRT; INCT – induction chemotherapy before CRT.

incomplete response (iCR) were evaluated at the end of TNT and are listed below in the MSK Regression Schema (see Section 11.2). Given the level of organ preservation observed (measured as organ preservation or TME-free survival rates), we feel that this is the optimal manner by which to enhance the cCR rates in patients who go on to WW in the Janus trial. Of note, 5-year data in the OPRA trial has since resulted and demonstrated stable organ preservation rates for the CNCT arm (54%) versus 39% in the INCT arm (p=0.012). Further, local regrowth rates remained lower in the CNCT arm (29%) versus 44% in the INCT arm (p=0.02)[45].

WW in context: WW for patients with rectal cancer with a cCR after LCRT was pioneered by Dr. Habr-Gama in 2004[32]. A recent pooled analysis of ~1000 patients with rectal cancer with a cCR and managed by W&W reported a 25% local regrowth rate, >90% salvage rate, and ~80% 5-year survival[24]. These results are consistent with the MSKCC experience with 113 patients managed by WW; a 21% local regrowth, a 91% salvage rate, and an 82% rectal preservation at 5 years[25]. The first randomized trial investigating the WW strategy in the US, the OPRA trial, has been reported at ASCO 2020 and is now published [PMID: 35483010], and demonstrates that WW can be incorporated safely using a TNT strategy with no decrement noted in 3-year DFS compared to historical controls, lower local regrowth rates than reported in retrospective series, and no differences in 3-year distant metastasis-free survival rates, local recurrence free

or overall survival[1]. Accrual in OPRA was brisk, owing to provider and patient interest in rectal preservation and QOL issues, and it was completed ahead of schedule.

In addition, to provide further rationale for selection of the CNCT arm as the control arm in the Janus trial, data from the OPRA trial shows a lower 3-year regrowth rate in the CNCT group (27%) compared to the INCT group (40%) (P=0.033, data not shown).

2.0 OBJECTIVES

2.1 Primary objective

Phase II: To evaluate and compare the cCR rates in patients with locally advanced rectal cancer treated with neoadjuvant LCRT followed by neoadjuvant mFOLFIRINOX versus neoadjuvant LCRT followed by neoadjuvant mFOLFOX6.

Phase III: To evaluate and compare disease-free survival (DFS) in patients with locally advanced rectal cancer treated with neoadjuvant LCRT followed by neoadjuvant mFOLFIRINOX versus neoadjuvant LCRT followed by neoadjuvant mFOLFOX6.

2.2 Secondary objective(s)

2.2.1 To evaluate and compare organ-preservation-time (OPT) between two treatment arms.

2.2.2 To evaluate and compare time to distant metastasis between two treatment arms.

2.2.3 To evaluate and compare overall survival (OS) between two treatment arms.

2.2.4 To evaluate and compare toxicity profiles of TNT between two treatment arms.

2.3 Exploratory objective

2.3.1 Evaluation of ctDNA kinetics during neoadjuvant therapy & surveillance and to correlate with radiographic, pathologic, and clinical outcomes.

3.0 PATIENT SELECTION

For questions regarding eligibility criteria, see the Study Resources page. Please note that the Study Chair cannot grant waivers to eligibility requirements.

3.1 On-Study Guidelines

This clinical trial can fulfill its objectives only if patients appropriate for this trial are enrolled. All relevant medical and other considerations should be taken into account when deciding whether this protocol is appropriate for a particular patient. Physicians should consider the risks and benefits of any therapy, and therefore only enroll patients for whom this treatment is appropriate.

- Physicians should consider whether any medical conditions would make this protocol unreasonably hazardous for the patient.
- Patients with a prior or concurrent malignancy whose natural history or treatment does not have the potential to interfere with the safety or efficacy assessment of the investigational neoadjuvant regimen or primary endpoint are eligible for this trial.

In addition:

- Women and men of reproductive potential should agree to use an appropriate method of birth control throughout their participation in this study due to the teratogenic potential of the therapy utilized in this trial. Appropriate methods of birth control include abstinence, oral contraceptives, implantable hormonal contraceptives or double barrier method (diaphragm plus condom).

3.2 Eligibility Criteria

Use the spaces provided to confirm a patient's eligibility by indicating Yes or No as appropriate. It is not required to complete or submit the following page(s).

When calculating days of tests and measurements, the day a test or measurement is done is considered Day 0. Therefore, if a test were done on a Monday, the Monday one week later would be considered Day 7.

A female of childbearing potential is a sexually mature female who: 1) has not undergone a hysterectomy or bilateral oophorectomy; or 2) has not been naturally postmenopausal for at least 12 consecutive months (i.e., has had menses at any time in the preceding 12 consecutive months).

___ **3.2.1 Documentation of Disease**

Histologic Documentation:

Stage: Clinical stage II or III rectal adenocarcinoma defined as T4N0 or any T with node positive disease (any T, N+); also T3N0 requiring APR or coloanal anastomosis

Tumor Site: Rectum; ≤ 12cm from the anal verge

___ **3.2.2 Prior Treatment**

No prior systemic chemotherapy, targeted therapy, or immunotherapy; or radiation therapy administered as treatment for colorectal cancer within the past 5 years is allowed.

___ **3.2.3 Not pregnant and not nursing,** because this study involves an agent that has known genotoxic, mutagenic and teratogenic effects.

Therefore, for women of childbearing potential only, a negative pregnancy test (urine or serum according to institutional guidelines) done ≤ 14 days prior to registration is required. Female subjects agree to use highly effective contraception combined with an additional barrier method (eg, diaphragm, with a spermicide) while on study and for ≥ 9 months after last dose of study drug, and the same criteria are applicable to male subjects if they have a partner of childbirth potential. Male subject agrees to use a condom and not donate sperm while in this study and for ≥ 6 months after the last treatment.

___ **3.2.4 Age ≥ 18 years**

___ **3.2.5 ECOG Performance Status 0-1 (or Karnofsky ≥ 60%)**

___ **3.2.6 Required Initial Laboratory Values:**

Absolute Neutrophil Count (ANC)	≥ 1,500/mm^3
Platelet Count	≥ 100,000/mm^3
Creatinine	≤ 1.5 x upper limit of normal (ULN) OR
Calc. Creatinine Clearance	≥ 50 mL/min
Total Bilirubin	≤ 1.5 x upper limit of normal (ULN)
AST / ALT	≤ 3 x upper limit of normal (ULN)

___ **3.2.7 Comorbid conditions**

- No upper rectal tumors (distal margin of tumor >12 cm from the anal verge)
- No recurrent rectal cancer; prior transanal excision, prior distal sigmoid cancer with a low anastomosis
- No known mismatch repair deficient rectal adenocarcinoma
- HIV-infected patients on effective anti-retroviral therapy with undetectable viral load within 6 months are eligible for this trial.
- Patients with known history or current symptoms of cardiac disease, or history of treatment with cardiotoxic agents, should have a clinical risk assessment of cardiac function using the New York Heart Association Functional Classification1. To be eligible for this trial, patients should be class 2B or better.

___ **3.2.8 Concomitant medications**

Chronic concomitant treatment with strong inhibitors of CYP3A4 is not allowed on this study. Patients on strong CYP3A4 inhibitors must discontinue the drug for 14 days prior to registration on the study.

Chronic concomitant treatment with strong CYP3A4 inducers is not allowed. Patients must discontinue the drug 14 days prior to the start of study treatment.

4.0 PATIENT REGISTRATION

4.1 Investigator and Research Associate registration with CTEP

Food and Drug Administration (FDA) regulations require sponsors to select qualified investigators. National Cancer Institute (NCI) policy requires all individuals contributing to NCI-sponsored trials to register with their qualifications and credentials and to renew their registration annually. To register, all individuals must obtain a Cancer Therapy Evaluation Program (CTEP) credentials necessary to access secure NCI Clinical Oncology Research Enterprise (CORE) systems. Investigators and clinical site staff who are significant contributors to research must register in the Registration and Credential Repository (RCR). The RCR is a self-service online person registration application with electronic signature and document submission capability.

RCR utilizes five person registration types.

- **Investigator (IVR)** — MD, DO, or international equivalent;
- **Non Physician Investigator (NPIVR)** — advanced practice providers (e.g., NP or PA) or graduate level researchers (e.g., PhD);
- **Associate Plus (AP)** — clinical site staff (e.g., RN or CRA) with data entry access to CTSU applications such as the Roster Update Management System (RUMS), OPEN, Rave; acting as a primary site contact, or with consenting privileges
- **Associate (A) —** other clinical site staff involved in the conduct of NCI-sponsored trials; and
- **Associate Basic (AB)** — individuals (e.g., pharmaceutical company employees) with limited access to NCI-supported systems.

RCR requires the following registration documents:

Documentation Required	IVR	NPIVR	AP	A	AB
FDA Form 1572	✓	✓			
Financial Disclosure Form	✓	✓	✓		
NCI Biosketch (education, training, employment, license, and certification)	✓	✓	✓		
GCP training	✓	✓	✓		
Agent Shipment Form (if applicable)	✓				
CV (optional)	✓	✓	✓		

In addition, IVRs and NPIVRs must list all clinical practice sites and Institutional Review Boards (IRBs) covering their practice sites on the FDA Form 1572 in RCR to allow the following:

- Addition to a site roster;
- Selection as the treating, credit, or drug shipment investigator or consenting person in OPEN;
- Ability to be named as the site-protocol Principal Investigator (PI) on the IRB approval; and
- Assignment of the Clinical Investigator (CI) task on the Delegation of Tasks Log (DTL).

In addition, all investigators acting as the Site-Protocol PI (investigator listed on the IRB approval), consenting/treating/drug shipment investigator in OPEN, or as the CI on the DTL must be rostered at the enrolling site with a participating organization.

Refer to the NCI RCR page on the CTEP website for additional information. For questions, please contact the **RCR Help Desk** by email at RCRHelpDesk@nih.gov.

4.2 Cancer Trials Support Unit registration procedures

Permission to view and download this protocol and its supporting documents is restricted and is based on the person and site roster assignment housed in the Roster Maintenance application and in most cases viewable and manageable via the Roster Update Management System (RUMS) on the Cancer Trials Support Unit (CTSU) members' website.

This study is supported by the NCI CTSU.

IRB Approval

As of March 1, 2019, all U.S.-based sites must be members of the NCI Central Institutional Review Board (NCI CIRB) in order to participate in Cancer Therapy Evaluation Program (CTEP) and Division of Cancer Prevention (DCP) studies open to the National Clinical Trials Network (NCTN) and NCI Community Oncology Research Program (NCORP) Research Bases. In addition, U.S.-based sites must accept the NCI CIRB review to activate new studies at the site after March 1, 2019. Local IRB review will continue to be accepted for studies that are not reviewed by the CIRB, or if the study was previously open at the site under the local IRB. International sites should continue to submit Research Ethics Board (REB) approval to the CTSU Regulatory Office following country-specific regulations.

Sites participating with the NCI CIRB must submit the Study Specific Worksheet (SSW) for Local Context to the CIRB using IRBManager to indicate their intent to open the study locally. The NCI CIRB's approval of the SSW is automatically communicated to the CTSU Regulatory Office, but sites are required to contact the CTSU Regulatory Office at CTSURegPref@ctsu.coccg.org to establish site preferences for applying NCI CIRB approvals across their Signatory Network. Site preferences can be set at the network or protocol level. Questions about establishing site preferences can be addressed to the CTSU Regulatory Office by email or calling 1-888-651-CTSU (2878).

Sites using their local IRB or REB, must submit their approval to the CTSU Regulatory Office using the Regulatory Submission Portal located in the Regulatory section of the CTSU website. Acceptable documentation of local IRB/REB approval includes:

- Local IRB documentation;
- IRB-signed CTSU IRB Certification Form; and/or
- Protocol of Human Subjects Assurance Identification/IRB Certification/Declaration of Exemption Form.

In addition, the Site-Protocol Principal Investigator (PI) (i.e., the investigator on the IRB/REB approval) must meet the following criteria for the site to be able to have an Approved status following processing of the IRB/REB approval record:

- Have an Active CTEP status;
- Have an active status at the site(s) on the IRB/REB approval on at least one participating organization's roster;
- If using NCI CIRB, be active on the NCI CIRB roster under the applicable Signatory Institution(s) record;

- Include the IRB number of the IRB providing approval in the Form FDA 1572 in the RCR profile;
- List all sites on the IRB/REB approval as Practice Sites in the Form FDA 1572 in the RCR profile; and
- Have the appropriate CTEP registration type for the protocol.

4.2.1 Additional site registration requirements

Additional site requirements to obtain an approved site registration status include:

- An active Federal Wide Assurance (FWA) number;
- An active roster affiliation with the Lead Protocol Organization (LPO) or a Participating Organization (PO)
- An active roster affiliation with the NCI CIRB roster under at least one CIRB Signatory Institution (US sites only); and
- Compliance with all applicable protocol-specific requirements (PSRs).

4.2.2 Protocol specific requirements for A022104 site registration

IROC credentialing:

This is a study with a radiation and/or imaging (RTI) component and the enrolling site must be aligned to an RTI provider. To manage provider associations or to add or remove associated providers, access the Provider Association page from the Regulatory section on the CTSU members' website at https://www.ctsu.org/RSS/RTFProviderAssociation. Sites must be linked to at least one Imaging and Radiation Oncology Core (IROC) provider to participate on trials with an RTI component. Enrolling sites are responsible for ensuring that the appropriate agreements and IRB approvals are in place with their RTI provider. An individual with a primary role on a treating site roster can update the provider associations, though all individuals at a site may view provider associations. To find who holds primary roles at your site, view the Person Roster Browser under the RUMS section on the CTSU website.

IROC Credentialing Status Inquiry (CSI) Form – this form is submitted to IROC Houston to verify credentialing status or to begin a new modality credentialing process.

To complete protocol-specific credentialing the RTI provider or enrolling site should follow instructions in the protocol to submit documentation or other materials to the designated IROC Quality Assurance (QA) center. Upon the IROC QA center approving the RTI provider for the study modality, IROC will automatically send the approval to the Regulatory and Roster Maintenance applications to comply with the protocol specific requirement unless otherwise noted at the bottom of the IROC Credentialing Approval notification. IROC will continue to copy the provider and/or enrolling site on modality approvals.

Upon site registration approval in the Regulatory application, the enrolling site may access OPEN to complete enrollments. The enrolling site will select their credentialed provider treating the subject in the OPEN credentialing screen and may need to answer additional questions related to treatment in the eligibility checklist.

4.2.3 Downloading Site Registration Documents

Download the site registration forms from the protocol-specific page located on the CTSU members' website. Permission to view and download this protocol and its supporting

documents is restricted to institutions and their associated investigators and staff on a participating roster. To view/download site registration forms:

- Log on to the CTSU members' website (https://www.ctsu.org);
- Click on *Protocols* in the upper left of your screen
  - Enter the protocol number in the search field at the top of the protocol tree, or
  - Click on the By Lead Organization folder to expand, the select *Alliance,* and protocol number *A022104*;
- Click on *Documents, Protocol Related Documents, and* use the *Document Type* filter and select *Site Registration* to download and complete the forms provided. (Note: For sites under the CIRB initiative, IRB data will load automatically to the CTSU as described above).

4.2.4 Submitting regulatory documents

Submit required forms and documents to the CTSU Regulatory Office using the Regulatory Submission Portal on the CTSU members' website.

To access the Regulatory Submission Portal log in to the CTSU members' website, go to the *Regulatory* section and select *Regulatory Submission*.

Institutions with patients waiting that are unable to use the Regulatory Submission Portal should alert the CTSU Regulatory Office immediately by phone or email: 1-866-651-CTSU (2878), or CTSURegHelp@coccg.org to receive further instruction and support.

4.2.5 Checking site registration status

Site registration status may be verified on the CTSU members' website.

- Click on *Regulatory* at the top of the screen;
- Click on *Site Registration*; and
- Enter the site's 5-character CTEP Institution Code and click on Go.
  - Additional filters are available to sort by Protocol, Registration Status, Protocol Status, and/or IRB Type.

Note: The status shown only reflects institutional compliance with site registration requirements as outlined within the protocol. It does not reflect compliance with protocol requirements for individuals participating on the protocol or the enrolling investigator's status with NCI or their affiliated networks.

4.3 Patient Registration Requirements

4.3.1 Informed consent

The patient must be aware of the neoplastic nature of his/her disease and willingly consent after being informed of the procedure to be followed, the experimental nature of the therapy, alternatives, potential benefits, side-effects, risks, and discomforts. Current human protection committee approval of this protocol and a consent form is required prior to patient consent and registration.

Patients with impaired decision making capacity may be enrolled on this study, where institutional policy and IRB of record allow.

4.4 Patient registration/randomization procedures (Step 1)

The Oncology Patient Enrollment Network (OPEN) is a web-based registration system available on a 24/7 basis. OPEN is integrated with CTSU regulatory and roster data and with the LPOs' registration/randomization systems or the Theradex Interactive Web Response System (IWRS) for retrieval of patient registration/randomization assignment. OPEN will populate the patient enrollment data in NCI's clinical data management system, Medidata Rave.

Requirements for OPEN access:

- Active CTEP registration with the credentials necessary to access secure NCI/CTSU IT systems;
- To perform enrollments or request slot reservations: Must be on an LPO roster, ETCTN Corresponding roster, or participating organization roster with the role of Registrar. Registrars must hold a minimum of an Associate Plus (AP) registration type;
- If a Delegation of Tasks Log (DTL) is required for the study, the registrars must hold the OPEN Registrar task on the DTL for the site; and
- Have an approved site registration for the protocol prior to patient enrollment.

To assign an Investigator (IVR) or Non-Physician Investigator (NPIVR) as the treating, crediting, consenting, drug shipment (IVR only), or receiving investigator for a patient transfer in OPEN, the IVR or NPIVR must list the IRB number used on the site's IRB approval on their Form FDA 1572 in RCR. If a DTL is required for the study, the IVR or NPIVR must be assigned the appropriate OPEN-related tasks on the DTL.

Prior to accessing OPEN, site staff should verify the following:

- Patient has met all eligibility criteria within the protocol stated timeframes; and
- All patients have signed an appropriate consent form and Health Insurance Portability and Accountability Act (HIPAA) authorization form (if applicable).

Note: The OPEN system will provide the site with a printable confirmation of registration and treatment information. You may print this confirmation for your records.

Access OPEN at https://open.ctsu.org or from the OPEN link on the CTSU members' website. Further instructional information is in the OPEN section of the CTSU website at https://www.ctsu.org or https://open.ctsu.org. For any additional questions, contact the CTSU Help Desk at 1-888-823-5923 or ctsucontact@westat.com.

4.5 Stratification Factors and Treatment Assignments

The randomization routine is found in Section 13 (Statistical Considerations).

4.5.1 Stratification Factors

1) Tumor stage based on clinical evaluation: T4 vs. T1-3
2) Nodal stage based on clinical evaluation: N+ vs. N0
3) The distance from the lower edge of tumor to the anal verge: 0 to < 4cm; ≥ 4cm to < 8cm; ≥ 8cm to ≤12cm

4.5.2 Treatment Assignments

The factors defined in Section 4.5.1 will be used as stratification factors.

After the patient has been registered into the study, the values of the stratification factors will be recorded, and the patient will be assigned to one of the following treatment groups using the Pocock and Simon dynamic allocation procedure which balances the marginal distributions of the stratification factors between the treatment groups.

1) LCRT followed by mFOLFOX6 (or CAPOX)
2) LCRT followed by mFOLFIRINOX

5.0 STUDY CALENDAR

Laboratory and clinical parameters during treatment are to be followed using individual institutional guidelines and the best clinical judgment of the responsible physician. It is expected that patients on this study will be cared for by physicians experienced in the treatment and supportive care of patients on this trial.

Pre-study Testing Intervals

The pre-study testing intervals are guidelines only. When calculating days of tests and measurements, the day a test or measurement is done is considered Day 0. Therefore, if a test were done on a Monday, the Monday one week later would be considered Day 7.

To be completed ≤ 28 DAYS before registration: All laboratory studies, history and physical.

To be completed ≤ 42 DAYS before registration: Any X-ray, scan of any type or ultrasound which is utilized for tumor measurement per protocol.

To be completed ≤ 60 DAYS before registration: Any baseline exams used for screening, or any X-ray, scan of any type or ultrasound of uninvolved organs which is not utilized for tumor measurement.

ARM 1

**Evaluations during treatment – Arm 1 (LCRT THEN FOLFOX OR LCRT THEN CAPOX)**									
Study Week (+/- 14 days)*	Pre	10[7]	12	14	16	18	20	22	24	32-38[, 2]
History and physical**	X	X	X	X	X	X	X	X	X	X
Height	X									
Adverse Event Assessment	X	X	X	X	X	X	X	X	X	X
Colorectal surgeon eval	X	X								X
Med Onc[3]	X	X	X	X	X	X	X	X	X	X
Rad Onc	X	X								
DRE (digital rectal exam)	X	X								X
Sigmoidoscopy/ Proctosigmoidoscopy***	X	X								X
Biopsy[4]	X									
MRI Rectum	X									X
CT CAP[5]	X									X
CBC & diff[6]	X									
CMP & CEA	X	X								X
Pregnancy Test****	X									

ARM 2

Evaluations during treatment – Arm 2 (LCRT THEN mFOLFIRINOX)										
Study Week (+/- 14 days)*	Pre	10[7]	12	14	16	18	20	22	24	32-38, [2]
History and physical**	X	X	X	X	X	X	X	X	X	X
Height	X									
Adverse Event Assessment	X	X	X	X	X	X	X	X	X	X
Colorectal surgeon eval	X	X								X
Med Onc [3]	X	X	X	X	X	X	X	X	X	X
Rad Onc	X	X								
DRE(digital rectal exam)	X	X								X
Sigmoidoscopy/ Proctosigmoidoscopy***	X	X								X
Biopsy[4]	X									
MRI Rectum	X									X
CT CAP[5]	X									X
CBC & diff[6]	X									
CMP & CEA	X	X								X
Pregnancy Test****	X									

* Timing can vary based on institutional standards (for example, if a center waits longer than 14 days between starting chemotherapy after completion of LCRT this is not a protocol violation) as some centers wait 4-6 weeks after LCRT completion to start systemic chemotherapy. As such, exact week number from registration may vary and as such can be adjusted to reflect timing of start of chemotherapy.
** Weight, Pulse, BP. Performance Status will only be required at pre, week 10 and weeks 32-38.
*** Should be completed by the evaluating/treating surgeon for baseline evaluation and during treatment assessments to maintain continuity of the response assessment (as this is critical for the primary endpoint). Baseline characteristics that should be captured include distance from the anal verge, photos, and the percent circumference of the lumen involved by the tumor. If the surgeon evaluating the patient at baseline completed the colonoscopy that was diagnostic then this would be sufficient for the initial baseline endoscopic evaluation as long the baseline characteristics of the tumor were captured including distance from the anal verge, photos and baseline characteristics of the tumor (as above). Given the importance for the primary endpoint it is critical for a surgeon to be involved who is willing to evaluate the primary tumor for the endpoint throughout the duration of the trial. The surgeon involved should be affiliated with an NCTN hospital or hospital system. It is permissible for the evaluating surgeon to be at a separate institution from the treating medical and radiation oncology teams as long as there is continuity in the management and seamless sharing of relevant clinical data between the teams (assuming all teams are part of NCTN or NCTN-affiliated hospitals and the arrangement is convenient for the patient). Further, the evaluating surgeon must have access to the protocol, all relevant documents and the tumor response forms to participate.
**** For women of childbearing potential (see Section 3.2.3). Must be done ≤ 7 days prior to registration.

[1] Time of evaluation dependent on duration of neoadjuvant chemotherapy FOLFOX (16 weeks) or CAPOX (15 weeks)
[2] 8-12 weeks (+/- 4 weeks) after completion of all neoadjuvant therapy
[3] Patients will be seen and evaluated during neoadjuvant chemoradiation and chemotherapy with necessary laboratory evaluations per institutional guidelines. For neoadjuvant chemotherapy, recommendation is to be evaluated by medical oncology during (Arm 1) and (Arm 2) chemotherapy every two weeks (every three weeks for patients getting CAPOX) or as needed per institutional guidelines. In patients not seen and evaluated by medical oncology every cycle, adverse events must still be collected and reported every cycle by relevant research staff.
[4] Biopsy to confirm pathological diagnosis of rectal adenocarcinoma is REQUIRED
[5] CT of the Chest, Abdomen and Pelvis. Prefer with intravenous contrast, but per institutional standards based on patient's labs and medical condition.
[6] CBC & diff, CMP performed at baseline and with each cycle of chemotherapy or per institutional standards.
[7] Assessment by surgeon, medical and radiation oncology occurs in a multidisciplinary fashion to ensure the patient has tolerated LCRT well and does not have to occur exactly at week 10; simply should occur prior to initiation of chemotherapy and is meant to ensure appropriate transition to the medical oncology team as they begin systemic chemotherapy.

Evaluations during follow-up for WW patients (after completion of TNT with cCR and post-TNT restaging)

Years on study	Year 1 (Every 3 months)				Year 2 (Every 3 months)				Year 3 (Every 6 months)		Years 4-5** (Every 6 months)			
Months after post-TNT restaging (+/- 30 days)	**3**	**6**	**9**	**12**	**15**	**18**	**21**	**24**	**30**	**36**	**42**	**48**	**54**	**60**
History and Physical	X	X	X	X	X	X	X	X	X	X	X	X	X	X
Sigmoidoscopy/ Proctosigmoidoscopy	X	X	X	X	X	X	X	X	X	X	X	X	X	X
MRI Rectum		X		X		X		X		X		X		X
CT CAP[1]				X				X		X		X		X
CEA levels[2]	X	X		X	X	X		X	X	X	X	X	X	X

*If TME completed via abdominoperineal resection – not relevant and unnecessary
**MRI in years 4 through 8 would only be supported if clinically indicated by treating team.

[1] CT of the Chest, Abdomen and Pelvis Patients in the TME group will be followed according to NCCN guidelines.[1]
[2] After 36 months, CEA will be evaluated every 6 months up to five years, based on NCCN guidelines.

Evaluations during follow-up for TME patients (after definitive surgical resection)

Years on study	Year 1								Year 2								Year 3			Years 4-5**		
Months after treatment (+/- 30 days)	**3**	**4**	**5**	**6**	**9**	**10**	**11**	**12**	**15**	**16**	**17**	**18**	**21**	**22**	**23**	**24**	**30**	**36**	**42**	**48**	**54**	**60**
History and Physical	X	X	X	X	X	X	X	X	X	X	X	X	X	X	X	X	X	X	X	X	X	X
Sigmoidoscopy/ Proctosigmoidoscopy					X	X	X	X					X	X	X	X		X		X		X
CT CAP[1]					X	X	X	X					X	X	X	X		X		X		X
CEA levels[2]	X	X	X	X	X	X	X	X	X	X	X	X	X	X	X	X	X	X	X	X	X	X

*If TME completed via abdominoperineal resection – not relevant and unnecessary
**MRI in years 4 through 8 would only be supported if clinically indicated by treating team.

[1] CT of the Chest, Abdomen and Pelvis Patients in the TME group will be followed according to NCCN guidelines.[1]
[2] After 36 months, CEA will be evaluated every 6 months up to five years, based on NCCN guidelines.

6.0 DATA AND SPECIMEN SUBMISSION

6.1 Data Collection and Submission

6.1.1 Data submission schedule

A Data Submission Schedule (DSS) is available on the Alliance study webpage, within the Case Report Forms section. The Data Submission Schedule is also available on the CTSU site within the study-specific Case Report Forms folder.

6.1.2 Medidata Rave

Medidata Rave is the clinical data management system being used for data collection for this trial/study. Access to the trial in Rave is controlled through the CTEP-IAM system and role assignments.

Requirements to access Rave via iMedidata:

- Active CTEP registration with the credentials necessary to access secure NCI/CTSU IT systems; and
- Assigned a Rave role on the LPO or PO roster at the enrolling site of: Rave CRA, Rave Read Only, Rave CRA (LabAdmin), Rave SLA, or Rave Investigator.

Rave role requirements:

- Rave CRA or Rave CRA (Lab Admin) role must have a minimum of an Associate Plus (AP) registration type;
- Rave Investigator role must be registered as a Non-Physician Investigator (NPIVR) or Investigator (IVR); and
- Rave Read Only or Rave SLA role must have at a minimum an Associate (A) registration type.

Refer to https://ctep.cancer.gov/investigatorResources/default.htm for registration types and documentation required.

This study has a Delegation of Tasks Log (DTL). Therefore, those requiring write access to Rave must also be assigned the appropriate Rave tasks on the DTL.

Upon initial site registration approval for the study in the Regulatory application, all persons with Rave roles assigned on the appropriate roster will be sent a study invitation email from iMedidata. No action will be required; each study invitation will be automatically accepted and study access in Rave will be automatically granted. Site staff will not be able to access the study in Rave until all required Medidata and study-specific trainings are completed. Trainings will be in the form of electronic learnings (eLearnings) and can be accessed by clicking on the eLearning link in the Tasks pane located in the upper right corner of the iMedidata screen. If an eLearning is required for a study and has not yet been taken, the link to the eLearning will appear under the study name in the Studies pane located in the center of the iMedidata screen; once the successful completion of the eLearning has been recorded, access to the study in Rave will be granted, and a Rave EDC link will replace the eLearning link under the study name.

No action will be required by site staff (to activate their account) who have not previously activated their iMedidata/Rave account at the time of initial site registration approval for the study in the Regulatory application. Pending study invitations (previously sent but not accepted or declined by a site user) will be automatically accepted and study access in Rave will be automatically granted for the site user. Account activation instructions are located

on the CTSU website in the *Data Management* section under the Data Management Help Topics > Rave resource materials (*Medidata Account Activation and Study Invitation*). Additional information on iMedidata/Rave is available on the CTSU members' website in the *Data Management* > *Rave section* or by contacting the CTSU Help Desk at 1-888-823-5923 or by email at ctsucontact@westat.com.

6.1.3 Data Quality Portal

The Data Quality Portal (DQP) provides a central location for site staff to manage unanswered queries and form delinquencies, monitor data quality and timeliness, generate reports, and review metrics.

The DQP is located on the CTSU members' website under Data Management. The Rave Home section displays a table providing summary counts of Total Delinquencies and Total Queries. DQP Queries, DQP Delinquent Forms, DQP Form Status, and the DQP Reports modules are available to access details and reports of unanswered queries, delinquent forms, forms with current status, and timeliness reports. Site staff should review the DQP modules on a regular basis to manage specified queries and delinquent forms.

The DQP is accessible by site staff who are rostered to a site and have access to the CTSU website. Staff who have Rave study access can access the Rave study data via direct links available in the DQP modules.

CTSU Delinquency Notification emails are sent to primary contacts at sites twice a month. These notifications serve as alerts that queries and/or delinquent forms require site review, providing a summary count of queries and delinquent forms for each Rave study that a site is participating in. Additional site staff can subscribe and unsubscribe to these notifications using the CTSU Report and Information Subscription Portal on the CTSU members' website.

To learn more about DQP use and access, click on the Help Topics button displayed on the Rave Home, DQP Queries, DQP Delinquent Forms, DQP Form Status, and DQP Reports modules.

6.1.4 Supporting documentation to be submitted to the Alliance

This study requires supporting documentation for diagnosis, response, progression, disease recurrence, and surgical outcomes. Supporting documentation will include pathology, radiology, endoscopy, digital rectal exam and surgery reports along with tumor response assessment forms (TRAFs) filled in by the treating surgeon who decides on WW or TME for the patient. These must be submitted at the following time points:

- Baseline: radiology and endoscopy reports supporting diagnosis
- End of TNT, surveillance and prior to surgery: radiology, endoscopy and digital rectal exam reports along with TRAFs (for those on WW)
- Time of surgery: surgery and pathology reports
- Time of recurrence or disease progression: radiology or pathology report indicating recurrence or disease progression

Supporting documentation is to be submitted via Rave.

6.2 Specimen collection and submission

The Alliance A022104 Correlative Science Manual (CSM) contains instructions for specimen collection, processing and shipping. The manual can be found on the BioMS and CTSU websites. Questions regarding the CSM should be addressed to the contacts specified in the manual.

For patients consenting to biobanking: All participating institutions must ask patients for their consent to participate in biobanking for future research, although patient participation is optional. Rationale and methods for the scientific components of these studies are described in Section 14.0. For patients who consent to participate, tissue and blood will be collected at the time points listed below for these studies:

**WW**	**After Registration/Prior to Initiation of Study Treatment**	**After Chemo-radiation/ Prior to first cycle of Consolidative Chemotherapy**	**At time of 1st re-staging**	**N/A**	**4 months +/- 2 months after 1st re-staging**	**8 months +/- 2 months after 1st re-staging**	**12 months +/- 2 months after 1st re-staging**	**At Progression[2]**
**Surgery**	**After Registration/Prior to Initiation of Study Treatment**	**After Chemo-radiation/ Prior to first cycle of Consolidative Chemotherapy**	**At time of 1st re-staging**	**Surgery**	**4 months +/- 2 months post surgery**	**8 months +/- 2 months post surgery**	**12 months +/- 2 months post surgery**	**At Progression[2]**
Tumor Tissue*	X			X[1]				X
Plasma and Buffy coat from EDTA tubes**	3 x 10 mL	3 x 10 mL	3 x 10 mL		3 x 10 mL	3 x 10 mL	3 x 10 mL	3x10ml

1. For patients going through surgery after study chemotherapy only
2. Progression here includes recurrence, regrowth and/or metastases. The method for research tissue procurement (endoluminal biopsy with forceps through the scope as per standard practice (rectal cancer biopsies), needle core biopsy (for metastatic site sampling), and/ or sampling of surgically resected tumor (for patients with tumor recurrence or regrowth undergoing salvage surgery)) is dependent on the disease site, clinical scenario, and individual patient.

* Retrieved fresh tissue can be formalin fixed and paraffin embedded (FFPE) as per institutional standard operating procedures (SOPs). Please refer to the correlative science procedure manual (CSM) for complete details.

** Buffy coat collection is only required at the After Registration/Prior to Initiation of Study Treatment time point.

6.3 Digital radiation therapy data submission using Transfer of Images and Data (TRIAD)

Transfer of Images and Data (TRIAD) is the American College of Radiology's (ACR) image exchange application. TRIAD provides sites participating in clinical trials a secure method to transmit images. TRIAD anonymizes and validates the images as they are transferred.

6.3.1 TRIAD Access Requirements

- Active CTEP registration with the credentials necessary to access secure NCI/CTSU IT systems;
- Registration type of: Associate (A), Associate Plus (AP), Non-Physician Investigator (NPIVR), or Investigator (IVR). Refer to the CTEP Registration Procedures section for instructions on how to request a CTEP-IAM account and complete registration in RCR; and
- TRIAD Site User role on an NCTN, ETCTN, or other relevant roster.

All individuals on the Imaging and Radiation Oncology Core provider roster have access to TRIAD and may submit images for credentialing purposes, or for enrollments to which the provider is linked in OPEN.

6.3.2 TRIAD Installations

To submit images, the individual holding the TRIAD Site User role will need to install the TRIAD application on their workstation. TRIAD installation documentation is available at https://triadinstall.acr.org/triadclient/.

This process can be done in parallel to obtaining your CTEP-IAM account and RCR registration.

For questions, contact TRIAD Technical Support staff via email TRIAD-Support@acr.org or 1-703-390-9858.

See Section 7.1 for details regarding submitting radiation therapy data via TRIAD.

7.0 TREATMENT PLAN/INTERVENTION

Protocol treatment is to begin ≤ 28 days of registration.

For questions regarding treatment, please see the study contacts page.

It is acceptable for individual chemotherapy doses to be delivered ≤ a 24-hour (1 business day) window before and after the protocol-defined date for Day 1 of a new cycle. For example, if the treatment due date is a Friday, the window for treatment includes the preceding Thursday through the following Monday. In addition, patients are permitted to have a new cycle of chemotherapy delayed up to 7 days for major life events (e.g., serious illness in a family member, major holiday, vacation that cannot be rescheduled) without this being considered a protocol violation. Documentation to justify this delay should be provided.

Protocol therapy will consist of neoadjuvant chemoradiation followed by neoadjuvant consolidation chemotherapy. Neoadjuvant chemoradiation includes radiation (45Gy + 9 Gy boost) in combination with a fluoropyrimidine (preferred capecitabine; permissible substitution: 5-Fluorouracil continuous infusion).

Subsequently, patients will receive eight cycles of chemotherapy with mFOLFOX6 (may be substituted by 5 cycles of CAPOX) in the control arm or eight cycles of chemotherapy with mFOLFIRINOX in the experimental arm. All patients will undergo assessment 8-12 weeks post-completion of this therapy for the primary endpoint of cCR and based on this assessment recommended further management with watch and wait, repeat assessment 8 weeks later, or surgery

In patients requiring surgery, it may begin any time after assessment for primary endpoint, i.e. 8-12 weeks following completion of chemotherapy.

7.1 Neoadjuvant Chemoradiotherapy

For specific questions or concerns regarding this section, please contact Dr. William A. Hall- whall@mcw.edu and/or Dr. Paul B. Romesser- *romessep@mskcc.org*.

Protocol treatment with long course chemoradiation therapy should be initiated within 28 days following protocol registration.

During radiation, a fluoropyrimidine must be administered concomitantly (preferred capecitabine 1650 mg/m^2/d PO in two divided doses, rounded as appropriately per institutional guidelines Monday-Friday on radiation days; Permissible Substitution: 5-Fluorouracil continuous infusion 225 mg/m^2/day, Monday-Friday on radiation days or 7 days per week based on preference of the site). If in the opinion of the investigator, a toxicity is considered to be due solely to the radiation, then the radiation can be held at the investigator's discretion. The rationale for treatment break and the duration of the break needs to be documented. Investigators are encouraged to reach out to Dr. William A. Hall and/or Dr. Paul B. Romesser to discuss concerns with radiation toxicity resulting in a treatment break.

Note: Intensity modulated radiation therapy (IMRT) is permitted.

Technical Factors: Megavoltage equipment (minimum acceptable energy is 6 MV) with image guidance capable of delivering either 3D conformal radiation or static intensity modulation with a multileaf collimator or dynamic intensity modulation (using a multileaf collimator or tomotherapy) is required.

CT Simulation

Patients will be simulated (and treated) in either prone or supine positioning. Immobilization is required. A belly board, alpha cradle or body vac fix is recommended. Rectal contrast is permitted along with a radio opaque marker of the location of the anal verge. CT slice thickness must be no greater than 3 mm. Oral contrast is optional during the CT sim, but can be used. If oral contrast is used, then approximately 10 ml of omnipaque can be mixed with 120 ml of water mix for the CT simulation and two-thirds of this ingested approximately 30 minutes prior to simulation. IV contrast is also recommended in the clinically node positive setting. To help protect the small intestine a comfortably full bladder is recommended. Patients should be simulated in the "arms up" position whether prone or supine. If a patient has limited range of motion in the shoulders or is limited by pain the arms can be placed on the high chest in the supine position.

Diagnostic MRI registration (highly encouraged):

If a diagnostic MRI was acquired for staging, then these images should be registered to the CT simulation. This can be done using the Axial T2 or Sagittal T2 weighted images. This is often using a rigid registration method, deformable registration is typically not necessary for this registration.

MR simulation (optional)

Patients must be set up in the treatment position on an MR-compatible, flat, table-top couch insert and be immobilized, using the devices created during CT simulation. Phased-array receiver coils should be utilized to facilitate use of parallel imaging. Images should be acquired in the transverse plane, using navigator gating. The imaging protocol should include T2-weighted images, diffusion-weighted images (DWI), fat-suppressed T1-weighted images and fat-suppressed, late arterial phase, postcontrast T1-weighted images. High-order shimming over a reduced volume fully encompassing the patient is strongly recommended prior to any fat-suppressed T1-weighted imaging.

Upon completion of the MR simulation and before transfer to a radiation treatment planning system, all images must be corrected for gradient nonlinearity-induced geometric distortion by applying a vendor-provided 3D distortion correction algorithm. MR Simulation should be registered with CT simulation using image registration software.

Target Volume Definitions

The Gross Tumor Volume (GTV): defined as all known gross disease as determined from a combination of physical exam, colonoscopy, ultrasound, CT, MRI, and/or PET-CT (if acquired). When possible, it is best practice to co-register imaging in the planning system, or image support software to help guide volume delineation.

a) The primary GTV and nodal GTV will be contoured separately. The primary GTV should be defined as the area of gross tumor within the rectum. Here co-registration of the diagnostic MRI may help delineate the GTV using primarily the T2 sequences.

b) Nodal GTV should be defined on either the CT or the MRI. Dimensions for what should be considered "clinical node positivity" are included below. Clinically positive nodes, such as inguinal nodes, should also be contoured as nodal GTV.

Table 7.1.1. Radiological criteria of suspicious lymph nodes on baseline MRI rectum.

Anatomic Location	Baseline MRI rectum
**TME** (mesorectal, superior rectal)	Short axis > 9 mm or<br>Short axis 5-9 mm and at least 2 morphologic criteria*or<br>Short axis < 5 mm and at least 3 morphologic criteria* or
**Internal iliac and obturator**	Short axis > 7 mm
**M1** (inguinal, external iliac, common iliac, retroperitoneal)	Short axis > 10 mm
**Presence of mucin**	Suspicious regardless the size of location

* Suspicious morphologic criteria: round shape, irregular borders, heterogeneous signal intensity

Clinical Target Volume (CTV_45) includes the GTV areas at risk for harboring microscopic disease. The CTV 45 should include the mesorectum (perirectal fat and the presacral space) and internal iliac lymph nodes. The CTV for a T4 tumor will include these same structures but should also include the external iliac lymph nodes. If the external iliac nodes or inguinal nodes are felt to be at risk for micrometastases by the radiation oncologist (for example, distal tumors involving the anal sphincter complex) or tumors with anterior extension and nodes in the external iliac region, they can be contoured and included at the discretion of the radiation oncologist. If treating the inguinal lymph nodes prophylactically IMRT is required. See the NRG contouring atlas as a guide at https://www.nrgoncology.org/Portals/0/Scientific%20Program/CIRO/Atlases/AnorectalContouringGuidelines.pdf.

CTV_54: Is defined as the gross disease (primary and nodal disease) plus a 1.5-2 cm margin, along with the entire mesorectum (in the region of the tumor and any gross nodes, CT sim slices that include gross disease) cropped on each CT sim slice out of barriers to regional spread (for example, this structure should NOT extend into local normal structures, such as the sacrum, bladder, or uninvolved skeletal muscle). The CTV should be modified slice by slice and barriers to anatomic spread should be excluded.

Planning Target Volume (PTV_45): *CTV_45 + 0.5 – 1.0 cm symmetric margin.*

PTV_54: *CTV 54 + 0.5 – 1.0 cm margin (this should be the same, or slightly smaller, than the PTV_45 margin)*

Prescription Dose and Fractionation

IMRT is not required.

3 or 4-Field 3D-CRT Treatment Planning:

- PTV45: The total dose to the PTV45 will be 45 Gy delivered in 25 fractions of 1.8 Gy per day.
- PTV54: The total dose to the PTV54 will include the initial 45 Gy followed by a sequential "cone-down" boost of 9 Gy delivered in 5 fractions of 1.8 Gy per day (total dose of 54 Gy).

IMRT Planning: (dose painted OR sequential IMRT boost strategies are permitted)

Dose painting for simultaneous integrated boost (SIB)

- PTV45: The total dose to the PTV45 will be 45 Gy delivered in 27 fractions of 1.67 Gy per day.
- PTV54: The total dose to the PTV54 will be 54 Gy delivered in 27 fractions of 2.0 Gy per day.

Sequential Boost:

- PTV45: The total dose to the PTV45 will be 45 Gy delivered in 25 fractions of 1.8 Gy per day.
- PTV54: A cone down to the PTV54 will be 9 Gy delivered in 5 fractions of 1.8 Gy per day (PTV54 total dose 45+9=54 Gy).

Table 7.1.2		
Structure	Constraint, #Fx	
PTV Constraints		
	Ideal	Acceptable
PTV_45	V(4500 cGy) ≥ 95%.	V(4500 cGy) ≥ 80%
PTV_54	V(boost dose) ≥ 95%<br>Max dose less than 110%<br>Min dose is more than 80%	V(boost dose) ≥ 70%<br>Max dose is 115%<br>Min dose is 60%
OAR Constraints		
Small Bowel	V(≥ 4500 cGy) ≤ 150cc<br>V(≥ 5000 cGy) ≤ 30 cc	
	Dmax < 5500 cGy	Dmax < 6000
Colon (uninvolved)	V(≥ 3000 cGy) ≤ 200 cc	n/a
	V(≥ 3500 cGy) ≤ 150 cc	n/a
	Dmax < 5500 cGy	Dmax < 6000

Femur_L	V(≥ 3000 cGy) ≤ 50%	n/a
	V(≥ 4000 cGy) ≤ 35%	n/a
	V(≥ 4400 cGy) ≤ 5%	n/a
	Dmax < 5200 cGy	Dmax < 5500 cGy
Femur_R	V(≥ 3000 cGy) ≤ 50%	n/a
	V(≥ 4000 cGy) ≤ 35%	n/a
	V(≥ 4400 cGy) ≤ 5%	n/a
	Dmax < 5200 cGy	Dmax < 5500 cGy
External Gen (Vulva or Testes)	V(≥ 2000 cGy) ≤ 50%	n/a
	V(≥ 3000 cGy) ≤ 35%	n/a
	V(≥ 4000 cGy) ≤ 5%	V(≥ 4000 cGy) ≤ 10%
Bladder	V(≥ 3500 cGy) ≤ 50%	n/a
	V(≥ 4000 cGy) ≤ 35%	n/a
	V(≥ 5000 cGy) ≤ 5%	V(≥ 5000 cGy) ≤ 45%
	Dmax < 5500 cGy	Dmax < 6000 cGy

Note: An unacceptable deviation will also be assessed if there is incomplete contouring of the entire GTV.

Definition of Critical Structures and Margins

Note: All structures must be named for digital RT data submission as listed in the table below. The structures marked as "Required" in the table must be contoured and submitted with the treatment plan. Structures marked as "Required when applicable" must be contoured and submitted when applicable. Resubmission of data may be required if labeling of structures does not conform to the standard name listed. Capital letters, spacing and use of underscores must be applied exactly as indicated.

**Table 7.1.3**		
**Standard Name**	**Description**	**Validation**
Bowel_Large	Colon (uninvolved with tumor) for each CT sim image slice that includes the PTV with 3 slices above and below	Required
Bowel_Small	Loops should be contoured for each CT sim image slice that includes the PTV with 3 slices above and below	Required
Femur_L, Femur_R	Full neck of the femur through to the greater trochanter	Required
Skin	3 mm rind on periphery of patient	Required
SpinalCord	The spinal cord is contoured as the true spinal cord, not the spinal canal.	Required
Bladder	Entire extent of the bladder as seen on the CT sim	Required
Stomach_PRV05	Stomach + 0.5 cm symmetric margin	Required
External	External contour encompassing all patient anatomy	Required

Detailed Specifications for contouring normal organs:

Organs at risk should be contoured on each CT sim image slice encompassing the PTV and expanding 3 slices above and 3 slices below the PTV.

Bowel_Large: The large bowel encompasses the caecum, ascending colon, transverse colon, descending colon, and sigmoid colon in one contour. Contour from the ileocaceal junction to the recto-sigmoid junction. The large bowel can be discriminated from the small bowel by the appearance of bowel contents, presence of haustra, sacculations, and appendices epiploicae. The contour adheres closely to the outer boundary of the external wall and includes large bowel contents. This should not be including any regions involved with tumor.

Bowel_Small: Individual loops of bowel seen on the CT simulation. The small bowel encompasses the duodenum, jejunum, and ileum in one contour. Contour from the pylorus to the ileocaecal junction. Ensure small bowel in the lower pelvis caudal to the recto-sigmoid junction is included. The small bowel can be discriminated from the large bowel by the appearance of bowel contents and the presence valvulae conniventes. The contour adheres closely to the outer boundary of the external wall and includes small bowel contents.

Bladder: Bladder should be contoured in its entirety

Femurs: Full neck of the femur through to the greater trochanter

7.1.1 Radiation Therapy Quality Assurance

Submission of treatment plans in digital format as DICOM RT is required. Digital data must include CT scans, structures, plan, and dose files. This study uses TRIAD for RT data submission. Use of TRIAD requires several preliminary steps (see Section 6.3). Additional information is available at: https://triadinstall.acr.org/triadclient/

Any items on the list below that are not part of the digital submission may be included with the transmission of the digital RT data. https://irocri.qarc.org/

Within 3 days of starting radiotherapy, simulation and treatment planning data must be submitted via TRIAD:

- Copies of pre-treatment CT and MRI studies and reports identifying the location of the primary rectal tumor.
- RT treatment plans in DICOMRT format, including treatment planning CT, structures, dose, and plan files.
- RT-1 Dosimetry Summary Form
- Treatment planning system summary report that includes the monitor unit calculations, beam parameters, calculation algorithm, and volume of interest dose statistics.

Within one week of completion of radiotherapy, the following data must be submitted via TRIAD for all patients:

- RT-2 Radiotherapy Total Dose Record Form
- A copy of the patient's radiotherapy record including the prescription and the daily and cumulative doses to all required areas.

Supportive data and forms should be included with the transmission of the digital RT data. Questions regarding the dose calculations or documentation should be directed to:

Protocol Dosimetrist

IROC Rhode Island QA Center

Phone: (401 753-7600

Email: mailto:physics@qarc.org

7.2 Consolidation Neoadjuvant Chemotherapy

For specific questions or concerns regarding this section, please contact Dr. Arvind Dasari adasari@mdanderson.org-and/or Dr. Hanna Sanoff- mailto:hanna_sanoff@med.unc.edu

Choice of regimen (mFOLFOX6 for 8 cycles or CAPOX for 5 cycles) for control arm is per the investigator's choice.

Chemotherapy should preferably begin within 2-3 weeks following completion of radiation in patients with mild suggestive as grade 1 or less non- hematological toxicity) or no residual side effects from chemoradiation. In patients with more severe side effects, chemotherapy may be delayed up to 4 weeks at investigators' discretion; further delays must be discussed with the study PI.

Central venous access is strongly recommended.

Adverse events and results of laboratory safety assessments are to be reviewed prior to administration of study therapy.

Infusion times, concurrent and/or order of drug administration for all chemotherapy in this protocol may be modified per institutional guidelines.

Table 7.2.1: Treatment Regimen for Control Arm (mFOLFOX6 for 8 cycles)

Drug	Dose	Administration	Dosing Interval	Planned Duration
**Oxaliplatin**	85 mg/m^2	IV, given concurrently through separate lines connected by Y-line tubing, over 2 hours. *(see footnote **a**)*	Day 1 every 2 weeks	8 cycles (16 weeks)
**Leucovorin**	400 mg/m^2			
**5-Fluorouracil (5-FU)**	400 mg/m^2	IV bolus recommended infusion time of 2–4 minutes immediately following oxaliplatin/leucovorin infusion		
	2400 mg/m^2	IV continuous infusion over 46-48 hours (total dose)		

a Oxaliplatin is not compatible with normal saline solution or with 5-FU. The infusion line must be thoroughly flushed with D5W after administration with oxaliplatin. If oxaliplatin **is held**, administer leucovorin over 2 hours (preferred); however, administration time per institutional practice is permitted. Levoleucovorin can be substituted for leucovorin throughout this protocol, per institutional practice or as needed for drug availability, at a dose of 200 mg/m^2.

Table 7.2.2: Treatment regimen for Control Arm (CAPOX for 5 cycles)

Drug	Dose	Administration	Dosing Interval	Planned Duration
**Oxaliplatin**	130 mg/m2	IV given over 2 hours.<br>(see footnote **a**)	Day 1<br>every 3 weeks	5 cycles<br>(15 weeks)
**Capecitabine**	2000 mg/m2/day in two divided doses	By mouth<br>(see footnotes **b, c**)	Days 1-14<br>every 3 weeks	

a Oxaliplatin is not compatible with normal saline solution.

b Capecitabine should be taken in the morning and evening within 30 minutes after a meal (breakfast and dinner). Do not double up doses. If a dose is missed for any reason, skip the dose and take the next dose at the normal time.

c **Sites may provide** patients with a pill diary (Appendix III) to record capecitabine compliance. However, it is NOT required. Non-English speaking patients should be instructed to self-document their compliance.

Table 7.2.3: Treatment regimen for Experimental Arm (mFOLFIRINOX for 8 cycles)

Drug	Dose	Administration	Dosing Interval	Planned Duration
**Oxaliplatin**	85 mg/m^2	IV, given concurrently through separate lines connected by Y-line tubing, over 2 hours.<br>*(see footnote **a**)*	Day 1<br>every 2 weeks	8 cycles<br>(16 weeks)
**Leucovorin**	400 mg/m^2			
**Irinotecan**	150 mg/m^2	IV continuous infusion over 30-90 minutes<br>*(see footnote **b**)*		
**5-Fluorouracil (5-FU)**	2400 mg/m^2	IV continuous infusion over 46-48 hours (total dose)		

a Oxaliplatin is not compatible with normal saline solution or with 5-FU. The infusion line must be thoroughly flushed with D5W after administration with oxaliplatin. If oxaliplatin **is held**, administer leucovorin over 2 hours (preferred); however, administration time per institutional practice is permitted. Levoleucovorin can be substituted for leucovorin throughout this protocol, per institutional practice or as needed for drug availability, at a dose of 200 mg/m^2.

b Prophylactic or therapeutic administration of intravenous or subcutaneous atropine 0.25 mg-1.0 mg should be considered (unless clinically contraindicated). Following completion of the first irinotecan infusion, patients can remain in the treatment area for a minimum of 1 hour in case acute abdominal cramping occurs (local site standard practice for post-infusion management should prevail).

7.3 Surgery

All patients will undergo assessment at 8-12 weeks post-TNT completion. If patients have a cCR (no tumor on endoscopy, MRI, or clinical exam using the MSK regression schema) then patients may be offered a WW approach or TME depending on the outcome of a thorough discussion with their treating physician concerning the risks and benefits of each approach and both they and their physician agree with the intended approach. If the patients have an incomplete response (any evidence of residual tumor by endoscopy, MRI or clinical exam using the MSK regression schema) then they be recommended to undergo TME (not mandated and identical to OPRA). Of note, and as in OPRA, if patients have a near complete response (nCR) then patients can undergo a repeat assessment 8 weeks later (+/-2 weeks). If there is any evidence that the tumor has stopped responding, persists or regrows then the patient will be recommended to undergo TME (e.g. nCR that fails to evolve to a cCR at 20 weeks (+/- 4 weeks) post-completion of TNT OR local regrowth after initial cCR determination). If there is a discrepancy between MRI and endoscopy/DRE then endoscopy/DRE should take precedence in the decision making on whether to proceed to WW or TME.

7.3.1 Surgical Quality Assurance

We expect that board-certified surgeons who care for patients with rectal cancer and who do at least 5 total mesorectal excisions per year in a multi-disciplinary setting will participate in this trial.

7.4 Imaging

ALLIANCE A022104 prescribes limited image acquisition parameters, so any imaging not specified is expected to be done under the site's own standard of care. Specifically, CT, and/or MRIs must be acquired at the following times.

1. Baseline:
   a. MRI of the rectum

      High-resolution rectal MRI using phase-array MRI coil using either 1.5 or 3 Tesla scans are required. Bowel preparation, intravenous or rectal contrast, and endorectal coil are not necessary.

      Sagittal, axial, and coronal 2D T2-weighted sequences without fat saturation are recommended, including high-resolution T2-weighted images perpendicular to the plane of the tumor with slice thickness of 3-4 mm. For tumors in the lower third of the rectum, coronal parallel to the anal canal is recommended.

      Diffusion weighted images with at least b-value of 800 and apparent diffusion coefficient map are recommended.

8.0 DOSE AND TREATMENT MODIFICATIONS

8.1 Ancillary Therapy, Concomitant Medications, and Supportive Care

All supportive therapy for optimal medical care will be given during the study period at the discretion of the treating physician(s) within the parameters of the protocol and documented on each site's source documents as concomitant medication.

Herbal and Nutritional Supplement: The concomitant use of herbal therapies is generally not recommended, as their pharmacokinetics, safety profiles, and potential drug-drug interactions are generally unknown. However, the use of general nutritional foundation supplements will be allowed including: calcium with vitamin D and/or minerals, Omega3s (fish oil), Vitamin B6, Vitamin B12, basic multivitamins, L-glutamine, or probiotics oral supplements will be

permitted either at or below the recommended dosing by a healthcare provider. Herbal-based multivitamins are not allowed.

8.1.1 Patients should not receive any other treatment which would be considered treatment for the primary neoplasm or impact the primary endpoint. This includes any surgical intervention, radiotherapy, cryotherapy, systemic therapy, ablation, etc.

8.1.2 Patients should receive full supportive care while on this study. This includes blood product support, antibiotic treatment, and treatment of other newly diagnosed or concurrent medical conditions. All blood products and concomitant medications such as antidiarrheals, analgesics, and/or antiemetics received from the first day of study treatment administration until 30 days after the final dose will be recorded in the medical records.

8.1.3 G-CSF

- Use of growth factors during consolidative chemotherapy is permitted on this study, while use of growth factors during chemotherapy + XRT phase is prohibited. Do not administer G-CSF within 24 hours of chemotherapy. See Section 8.2 for treatment modifications/management. The use of growth factors should follow published guidelines of the American Society of Clinical Oncology Clinical Practice Guideline Update. J Clin Oncol 33: 3199-3212, 2015.
- Use of epoetin in this protocol is discouraged
- Choice of growth factor for neutropenia is at the investigator's discretion.
- If needed, pegfilgrastim and filgrastim are recommended; however, if required by institutional standards, GM-CSF may be administered as an alternative. Biosimilars are also permitted based on institutional practice.
- Loratadine (10 mg) or similar agents are permitted if pegfilgrastim is used.

8.1.4 Management of nausea or vomiting

Antiemetic therapy should be administered according to National Comprehensive Cancer Network (NCCN) (https://www.nccn.org) or American Society of Clinical Oncology (ASCO) clinical practice guidelines (Hesketh 2017). See Section 8.2 for dose modifications and delays.

8.1.5 Management of Diarrhea

Diarrhea is a commonly occurring toxicity with the therapies included in this protocol. Without appropriate treatment, diarrhea can be prolonged, severe, and lead to dehydration and other complications. (See Appendix I for clinical management of diarrhea.)

Inform patients that they may experience diarrhea while on chemotherapy and possibly for several weeks after chemotherapy has stopped.

Patients **must** be instructed to:

- have ready access to antidiarrheal agents (e.g., loperamide) starting on Day 1 of treatment.
- All patients must be instructed to begin taking loperamide at the earliest sign of poorly-formed or loose stools (≥ grade 1). Early intervention is important for patient safety. See Section 8.2 for dose modifications and delays.
- continue prophylactic therapy as directed

- promptly report diarrhea symptoms
- report constipation *before* taking any laxatives or stopping antidiarrheal medication.

Patients who have multiple loose bowel movements and any worsening of fatigue, nausea, vomiting, right upper quadrant abdominal pain or tenderness, fever, rash, or eosinophilia should be promptly evaluated for changes in liver function. (See Appendix I for sample patient instructions for diarrhea management.)

Aggressive supportive care should be provided for patients with grade 4 ANC and ≥ grade 3 diarrhea until neutropenia and diarrhea resolve. See Appendix I for clinical management of diarrhea. Hospitalization for evaluation and management of grade 3 or grade 4 complicated diarrhea, as defined in Appendix I, is strongly recommended.

Refer to the ASCO Recommended Guidelines for Treatment of Cancer Treatment-Induced Diarrhea for additional recommendations regarding diarrhea (Benson 2004).

8.1.6 Management of pharyngolaryngeal dysesthesias

Oxaliplatin may cause discomfort in the larynx or pharynx associated with the sensation of dyspnea, anxiety, and swallowing difficulty. Exposure to cold can exacerbate these symptoms.

Refer to Section 8.2 for dose modification instructions.

Do NOT use ice chips or other forms of oral cryotherapy to decrease stomatitis in conjunction with oxaliplatin.

Anxiolytics may be used at the physician's discretion.

8.1.7 Management of Irinotecan-related cholinergic syndrome (Experimental Arm)

- Lacrimation, rhinorrhea, miosis, diaphoresis, hot flashes, flushing, abdominal cramping, diarrhea, or other symptoms of early cholinergic syndrome may occur during or shortly after receiving irinotecan. Atropine, 0.25-1.0 mg IV or SC may be used to treat these symptoms or may be used prophylactically. Additional antidiarrheal measures may be used at the discretion of the investigator.
- Combination anticholinergic medications containing barbiturates or other agents (e.g., Donnatal®) should not be used because these may affect irinotecan metabolism. Anticholinergics should be used with caution in patients with potential contraindications (e.g., obstructive uropathy, glaucoma, tachycardia, etc.).
- Diarrhea developing more than 24 hours after the irinotecan dose should be managed with loperamide per protocol.

8.1.8 Hypersensitivity/infusion reactions

Treat hypersensitivity and infusion reactions to oxaliplatin per institutional standards.

8.1.9 Drug/drug interactions

- **Coumarin:** Altered coagulation parameters and/or bleeding, including death, have been reported in patients taking capecitabine concomitantly with coumarin-derivative anticoagulants such as warfarin and phenprocoumon. These events occurred within several days and up to several months after initiating capecitabine therapy and, in a few cases, within 1 month after stopping capecitabine. These events occurred in patients with and without liver metastases.

- **It is required that the INR be monitored carefully** (at least weekly) while the patient is receiving treatment with capecitabine and warfarin concurrently and for an additional 4 weeks following the patient's last capecitabine dose. Institutional standards for this drug combination should be followed closely. Subcutaneous heparin or fractionated heparin products are permitted.
- **Phenytoin:** Increased phenytoin levels have also been reported in patients taking capecitabine concurrently with phenytoin and, therefore, need to be monitored.
- Chronic concomitant treatment with strong inhibitors or inducers of CYP3A4 is not allowed on this study. Patients on strong CYP3A4 inhibitors or inducers must discontinue the drug for 14 days prior to registration on the study.

8.2 Dose Modifications

The CTCAE v5.0 must be used to grade the severity of AEs. Refer to http://ctep.cancer.gov/protocolDevelopment/electronic_applications/ctc.htm.

Deviations from the recommended treatment modifications/management must be documented in the patient treatment record.

In the event of disease recurrence, second primary colorectal cancer, or diagnosis of an invasive second primary/secondary malignancy, study therapy will be discontinued; further therapy is at the investigator's discretion. Tests, exams, and assessments **are required** and patients will continue to be followed off treatment for vital status per protocol.

If, in the opinion of the investigator, a toxicity is considered to be due solely to one component of the study treatment (i.e., 5-FU, capecitabine, oxaliplatin, or irinotecan) and the dose of that component is held or modified in accordance with the guideline below, the other components may be administered per protocol guidelines.

If several toxicities with different grades of severity occur at the same time, the dose modifications should be according to the highest grade observed.

Any chemotherapy doses that have been reduced may not be escalated.

For any concomitant conditions apparent at baseline, the dose modifications will apply according to the corresponding shift in toxicity grade, if the investigator feels it is appropriate. For example, if a patient has Grade 1 asthenia at baseline that increases to Grade 2 during treatment, this will be considered a shift of one grade and treated as Grade 1 toxicity for dose-modification purposes only.

For specific questions or concerns regarding this section, please contact Dr. Arvind Dasari adasari@mdanderson.org-and/or Dr. Hanna Sanoff- mailto:hanna_sanoff@med.unc.edu.
In the event of treatment-related AEs during consolidation chemotherapy that requires treatment hold for >=30 days from the date of the planned cycle, consolidation chemotherapy should be discontinued. If consolidation chemotherapy is discontinued because of delay, patients the subject should be referred for presurgical restaging as detailed in the study calendar in section 5.0
In the event of non-treatment related AEs that result in dose delays of 30-60 days and have resolved to grade <= grade 1 by 60 days from the date of the planned cycle, patients may continue with consolidation chemotherapy if deemed in their best interest by the treating oncologist.

8.2.1 Dose Levels

Chemotherapy dose modifications for patients are detailed in Tables 8.2.1.2 and 8.2.1.3. Dose modifications are based on the dose level changes outlined in Table 8.2.1.1. Additionally, the following mFOLFOX6 dose modification instructions must be followed:

All doses must be based on the AE requiring the greatest modification.

Any chemotherapy doses that have been reduced may not be escalated.

- If ≥ grade 2 toxicity occurs ***during the 46-48 hour infusion of 5-FU***, discontinue the infusion, and refer to Table 8.2.1.2 for dose modifications for the next cycle of mFOLFOX6.
- The leucovorin dose remains 400 mg/m^2 regardless of changes in the 5-FU and oxaliplatin doses. If 5-FU is held, leucovorin should also be held.
- If oxaliplatin is discontinued, treatment should continue with 5-FU and leucovorin.

Table 8.2.1.1. mFOLFOX6 dose levels

	**Dose Level 0** *Starting Dose* (mg/m^2)	**Dose Level -1** (mg/m^2)	**Dose Level -2** (mg/m^2)	**Dose Level -3**
**Oxaliplatin**	85	65	50	Discontinue
**Leucovorin***	400	400	400	Discontinue
**5-FU bolus**	400	320	270	Discontinue
**5-FU infusion**	2400	1920	1600	Discontinue
*Levoleucovorin at 200 mg/m^2 can be substituted for leucovorin per institutional practice or as needed for drug availability (see Appendix II).				

Table 8.2.1.2. Treatment management for mFOLFOX6 -(See Table 8.2.1.3 for oxaliplatin-specific toxicities.)

Important table instructions:

- All dose modifications for mFOLFOX6 are based on the dose level changes on Table 8.2.1.1.
- Dose modifications must be based on AEs that occurred during the cycle (column 2) ***and*** AEs present on the scheduled Day 1 of Cycles 2-12 (column 3).
- Refer to footnote **a** for management of anemia.
- Modifications in dose levels apply to 5-fluorouracil and oxaliplatin unless otherwise indicated; leucovorin doses remain unchanged.
- Dose modifications must be based on the AE requiring the greatest modification.

CTCAE v5.0 Adverse Event/Grade	Modifications for AEs that occurred during a cycle but RESOLVE PRIOR TO THE NEXT TREATMENT CYCLE (See footnote **b**)	Modifications for AEs that REQUIRE A DELAY IN ADMINISTRATION OF THE TREATMENT CYCLE (See footnote **c**)

**<u>Neutrophil count decreased</u>:**<br><br>Grades 2 (ANC 1500-1000/mm^3)	Maintain dose.<br>*Consider use of growth factors to avoid delay with subsequent cycles.*	*Consider use of growth factors to avoid delay with subsequent cycles.*<br><br>*Hold until ≥ 1500/mm^3. If recovery takes:*<br><br>1 wk – maintain dose;<br>≥ 2 wks – ↓ one dose level
**<u>Grade 3</u>**	Maintain dose<br><br>*Consider use of growth factors to avoid delay with subsequent cycles.*	*Consider use of growth factors to avoid delay with subsequent cycles.*<br><br>*Hold until ≥ 1500/mm^3. If recovery takes:*<br><br>1 wk – maintain dose;<br>≥ 2 wks – ↓ one dose level
**<u>Grade 4</u>**	↓ one dose level.<br><br>*Consider use of growth factors to avoid delay with subsequent cycles.*	*Consider use of growth factors to avoid delay with subsequent cycles.*<br><br>*Hold until ≥ 1500/mm^3.*<br><br>↓ one dose level.
**<u>Platelet count decreased</u>:**<br><br>Grade 2	Maintain dose	*Hold until ≥ 75,000/mm^3.*<br>*If recovery takes:*<br><br>1-3 wks – maintain dose<br><br>≥ 3 wks – ↓ one dose level
Grade 3,4	↓ one dose level	*Hold until ≥ 75,000/mm^3. ↓ one dose level*
**<u>GI</u>:**<br><br>**Diarrhea** *(despite optimal antidiarrheal management)*<br><br>Grade 2	Maintain dose	↓ only 5-FU one dose level
Grade 3	↓ only 5-FU one dose level	↓ only 5-FU one dose level

Table 8.2.1.2. Treatment management for mFOLFOX6 (continued)

CTCAE v5.0<br>Adverse Event/Grade	Modifications for AEs that occurred during a cycle but RESOLVE PRIOR TO THE NEXT TREATMENT CYCLE<br>**(**See footnote **b*)***	Modifications for AEs that REQUIRE A DELAY IN ADMINISTRATION OF THE TREATMENT CYCLE<br>**(**See footnote **c*)***
Grade 4	↓ 5-FU two dose levels *and*<br>↓ oxaliplatin one dose level	Discontinue
**Mucositis oral**	Maintain dose	↓ only 5-FU one dose level

Grade 2		
Grade 3	↓ only 5-FU one dose level	↓ 5-FU two dose levels and<br>↓ oxaliplatin one dose level
Grade 4	↓ 5-FU two dose levels and<br>↓ oxaliplatin one dose level	Discontinue
**Vomiting** *(despite optimal antiemetics)*<br>Grade 2	Maintain dose	↓ one dose level
Grades 3, 4	↓ one dose level	Discontinue
**Investigations (hepatic):**<br>**Bilirubin, AST, alk phos**<br>Grade 2	Maintain dose	*Hold until bilirubin returns to the* ***baseline grade*** *and AST and alk phos have returned to ≤grade 1, then:*<br>↓ oxaliplatin one dose level
Grade 3	↓ 5-FU *and* oxaliplatin one dose level	*Hold until bilirubin returns to the* ***baseline grade*** *and AST and alk phos have returned to ≤grade 1, then:*<br>↓ 5-FU *and* oxaliplatin two dose levels
Grade 4	Discontinue	Discontinue
**Febrile neutropenia:**<br>Grade 3	*Consider use of growth factors to avoid delay with subsequent cycles.*<br>↓ one dose level	*Consider use of growth factors to avoid delay with subsequent cycles.*<br>↓ one dose level
Grade 4	↓ one dose level	Discontinue
**Infection:**<br>Grade 2	Maintain dose	Maintain dose
Grade 3	↓ one dose level	↓ one dose level
Grade 4	↓ one dose level	Discontinue
**Other clinically significant AEs:**[d]<br>Grade 2	Maintain dose	Maintain dose
Grade 3	↓ one dose level	↓ one dose level
Grade 4	↓ one dose level	Discontinue

**a**	Chemotherapy should not proceed with ≥ grade 3 anemia. Transfusion is acceptable for improving the hemoglobin value to allow therapy to continue without delay. The patient should be assessed to rule out other causes of anemia. *Use of erythropoiesis-stimulating agents is discouraged*.
**b**	Resolved means that all clinically significant AEs are ≤ grade 1 (except bilirubin, which must be ≤ the baseline grade) on Day 1 of the next scheduled cycle (i.e., treatment can be given without delay).
**c**	Hold and check weekly. ***With exception of bilirubin, resume treatment when toxicity is ≤ grade 1.*** If toxicity has not resolved after 4 weeks of delay, discontinue mFOLFOX6
**d**	Determination of "clinically significant" AEs is at the discretion of the investigator.

Table 8.2.1.3. Treatment management for oxaliplatin-specific toxicities

**Nervous System Disorders**		
**Paresthesias/Dysesthesias/ Neuropathy (Peripheral motor; Peripheral sensory)**	**1-7 day duration** *(intermittent or continuous)*	**> 7 day duration[a]** *(intermittent or continuous)*
Grade 1	Maintain dose	Maintain dose
Grade 2	Maintain dose[a]	Decrease ***oxaliplatin*** one dose level[a]
Grade 3	**First episode**: Decrease ***oxaliplatin*** one dose level **a** **Second episode**: Discontinue ***oxaliplatin***	Discontinue ***oxaliplatin***
**Respiratory, thoracic and mediastinal disorders**		
**Laryngopharyngeal dysesthesia**	**1-7 day duration** *(intermittent or continuous)*	**> 7 day duration** *(intermittent or continuous)*
Grade 1<br>Grade 2	Maintain dose and consider increasing infusion time of oxaliplatin to 6 hours	Maintain dose and consider increasing infusion time of oxaliplatin to 6 hours
Grade 3	At the investigator discretion, either discontinue oxaliplatin or increase duration of infusion to 6 hours	Discontinue oxaliplatin
Grade 4	Discontinue oxaliplatin	Discontinue oxaliplatin
**Respiratory, thoracic and mediastinal disorders**		

**Dyspnea** ≥ grade 2<br>**Hypoxia** ≥ grade 2<br>**Pneumonitis/pulmonary infiltrates** ≥ grade 2<br>**Pulmonary fibrosis** ≥ grade 2<br>**Cough** ≥ grade 3	Hold **all therapy** until interstitial lung disease is ruled out.<br>• If non-infectious interstitial lung disease is confirmed, oxaliplatin must be discontinued.<br>• If non-infectious interstitial disease is ruled out and infection (if any) has resolved, patients with persistent Grade 2 dyspnea or hypoxia can resume treatment at the discretion of the investigator.
**a** ***Hold oxaliplatin for persistent moderate intensity grade 2 neuropathy***. When improved to ***less than moderate intensity***, resume treatment with dose modification for oxaliplatin. If ***moderate intensity Grade 2 toxicity*** persists after 4 weeks of delay, discontinue oxaliplatin. Continue 5-FU + LV while oxaliplatin is held.	

8.2.2 Treatment management for patients receiving CAPOX

Capecitabine dose modifications for patients treated with CAPOX are detailed in Table 8.2.2.2. See Tables 8.2.1.2 and 8.2.1.3 for oxaliplatin-specific toxicities. Dose modifications are based on the dose level changes outlined in Table 8.2.2.1.

Additionally, the following dose modification instructions must be followed:

All dose modifications should be based on the adverse event requiring the greatest dose modification.

If capecitabine is held or discontinued, oxaliplatin continues per protocol unless otherwise contraindicated.

Capecitabine and oxaliplatin doses that have been reduced may not be escalated.

Table 8.2.2.1. CAPOX (capecitabine per day in two divided doses) dose levels

	**Dose Level 0**<br>***Starting Dose***<br>**($mg/m^2/d$)**	**Dose Level -1**<br>**($mg/m^2/d$)**	**Dose Level -2**<br>**($mg/m^2/d$)**	**Dose Level -3**
**Oxaliplatin**	130	100	85	Discontinue
**Capecitabine**	2000	1500	1000	Discontinue

Table 8.2.2.2. Treatment management for capecitabine (See Tables 8.2.1.2 and 8.2.1.3 for oxaliplatin-specific toxicities.)

**Important table instructions**:<br>• Dose modifications for capecitabine are based on the dose level changes on Table 8.2.2.1.<br>• **Hold capecitabine until any AE requiring dose modification returns to ≤ grade 1** unless indicated otherwise in the treatment management sections/tables. If recovery to ≤ grade 1 (or to other level specified) has not occurred after 3 weeks of delay, study therapy must be discontinued.	
CTCAE v5.0<br>Adverse Event/Grade	Modifications for AEs that<br>REQUIRED DELAY IN TREATMENT

**<u>Neutrophil count decreased</u>:**<br>Grades 2 (ANC 1000-1500/mm^3), 3, 4	*Hold until ≥ 1500/mm^3.If recovery takes:*<br>1 wk – maintain dose;<br>2-3 wks – ↓ one dose level
**<u>Platelet count decreased</u>:**<br>Grades 2, 3	*Hold until ≥ 75,000/mm^3*<br>*If recovery takes:*<br>1 wk – maintain dose;<br>2-3 wks – ↓ one dose level
Grade 4	*Hold until ≥ 75,000/mm^3*<br>↓ one dose level

Table 8.2.2.2. Treatment management for capecitabine *(continued)*

CTCAE v5.0<br>Adverse Event/Grade	Modifications for AEs that<br>REQUIRED DELAY IN TREATMENT
Grade 1	Maintain dose level
Grade 2	***Treatment must be held until resolved to grade 0 or 1 to avoid severe complications.***<br>1st occurrence – Maintain dose level<br>2nd occurrence –↓ one dose level<br>3rd occurrence – ↓ one dose level<br>4th occurrence – discontinue
Grade 3	***Treatment must be held until resolved to grade 0 or 1 to avoid severe complications.***<br>1st occurrence -↓ one dose level<br>2nd occurrence - ↓ one dose level<br>3rd occurrence – discontinue
Grade 4	Discontinue permanently OR if the physician deems it to be in the patient's best interest to continue, interrupt until resolved to grade 0 – 1.<br>↓ two dose levels
***** Determination of "clinically significant" AEs is at the discretion of the investigator.	

8.2.3 Treatment management for patients receiving mFOLFIRINOX

mFOLFIRINOX dose modifications for patients treated with mFOLFIRINOX are detailed in Table 8.2.3.2. Dose modifications are based on the dose level changes outlined in Table 8.2.3.1.

Additionally, the following dose modification instructions must be followed:

All doses must be based on the AE requiring the greatest modification.

Any chemotherapy doses that have been reduced may not be escalated.

- If ≥ grade 2 toxicity occurs ***during the 46-48 hour infusion of 5-FU***, discontinue the infusion and refer to Table 8.2.3.2 for dose modifications for the next cycle of mFOLFIRINOX.
- The leucovorin dose remains 400 mg/m^2 regardless of changes in the 5-FU, oxaliplatin, and irinotecan doses. If 5-FU is held, leucovorin should also be held.
- If oxaliplatin is discontinued, treatment should continue with 5-FU, leucovorin, and irinotecan.
- If irinotecan is discontinued, treatment should continue with 5-FU, leucovorin, and oxaliplatin.
- If 5-FU and leucovorin are discontinued, treatment should continue with irinotecan and oxaliplatin.

Table 8.2.3.1: Dose levels for mFOLFIRINOX

	**Dose Level 0** ***Starting Dose*** **(mg/m^2)**	**Dose Level -1 (mg/m^2)**	**Dose Level -2 (mg/m^2)**	**Dose Level -3**
**Oxaliplatin**	85	65	50	Discontinue
**Leucovorin***	400	400	400	Discontinue
**5-FU infusion**	2400	1920	1600	Discontinue
**Irinotecan**	150	135	120	Discontinue
*Levoleucovorin at 200 mg/m^2 can be substituted for leucovorin per institutional practice or as needed for drug availability (see Appendix II).				

Table 8.2.3.2. Treatment management for mFOLFIRINOX -(See Table 8.2.1.3 for oxaliplatin-specific toxicities.)

**<u>Important table instructions</u>:** • All dose modifications for mFOLFIRINOX are based on the dose level changes on Table 8.2.3.1. • Dose modifications must be based on AEs that occurred during the cycle (column 2) ***and*** AEs present on the scheduled Day 1 of Cycles 2-12 (column 3). • Refer to footnote '**a**' for management of anemia. • Modifications in dose levels apply to 5-fluorouracil, irinotecan, and oxaliplatin unless otherwise indicated; leucovorin doses remain unchanged. • Dose modifications must be based on the AE requiring the greatest modification.		
CTCAE v5.0 Adverse Event/Grade	Modifications for AEs that occurred during a cycle but RESOLVE PRIOR TO THE NEXT TREATMENT CYCLE (See footnote **b**)	Modifications for AEs that REQUIRE A DELAY IN ADMINISTRATION OF THE TREATMENT CYCLE (See footnote **c**)

**<u>Neutrophil count decreased</u>:**<br>Grades 2 (ANC 1000-1500/mm^3)	Reduce irinotecan one dose level<br>*Consider use of growth factors to avoid delay with subsequent cycles.*	*Consider use of growth factors to avoid delay with subsequent cycles.*<br>*Hold until ≥ 1500/mm^3. If recovery takes:* 1-2 wks – maintain dose<br>≥2 wks - ↓ one dose level
Grade 3	Reduce irinotecan one dose level. Maintain dose level of other drugs<br>*Consider use of growth factors to avoid delay with subsequent cycles.*	Reduce irinotecan one dose level.<br>*Consider use of growth factors to avoid delay with subsequent cycles.*<br>Hold until ≥ 1500/mm^3.<br>For other drugs:<br>1 wk – maintain dose or ↓ one dose level at discretion of treating oncologist;<br>≥2 wks – ↓ one dose level
Grade 4	Reduce irinotecan one dose level. Maintain dose level of other drugs<br>*Consider use of growth factors to avoid delay with subsequent cycles.*	Reduce irinotecan two dose levels; reduce doses of other drugs by 1 dose level.<br>*Consider use of growth factors to avoid delay with subsequent cycles.*<br>Hold until ≥ 1500/mm^3.
**<u>Platelet count decreased</u>:**<br>Grade 2	Reduce irinotecan one dose level. Maintain dose level of other drugs	*Hold until ≥ 75,000/mm^3.*<br>If recovery takes: 1-3 wks – maintain dose or ↓ one dose level at discretion of treating oncologist
Grade 3	Reduce irinotecan one dose level. Maintain dose level of other drugs	*Hold until ≥ 75,000/mm^3.*<br>Reduce irinotecan one dose level. For other drugs ↓ one dose level
Grade 4	Reduce irinotecan one dose level. ↓ one dose level of other drugs	Reduce irinotecan two dose levels; reduce doses of other drugs by 1 dose level.<br>*Hold until ≥ 75,000/mm^3.*
**<u>GI</u>:**<br>**Diarrhea** *(despite optimal antidiarrheal management)*<br>Grade 2	↓ irinotecan one dose level	↓ 5-FU *and* irinotecan one dose level
Grade 3	↓ 5-FU, oxaliplatin, *and* irinotecan one dose level	↓ 5-FU, oxaliplatin, *and* irinotecan one dose level

Grade 4	↓ 5-FU *and* oxaliplatin one dose level, irinotecan two dose levels	Discontinue
**Mucositis oral**<br>Grade 2	Maintain dose	↓ only 5-FU one dose level
Grade 3	↓ only 5-FU one dose level	↓ 5-FU two dose levels *and*<br>↓ irinotecan *and* oxaliplatin one dose level
Grade 4	↓ 5-FU two dose levels *and*<br>↓ irinotecan *and* oxaliplatin one dose level	Discontinue

Table 8.2.3.2. Treatment management for mFOLFIRINOX *(continued)*

CTCAE v5.0<br>Adverse Event/Grade	Modifications for AEs that occurred during a cycle but RESOLVE PRIOR TO THE NEXT TREATMENT CYCLE<br>(See footnote **b**)	Modifications for AEs that REQUIRE A DELAY IN ADMINISTRATION OF THE TREATMENT CYCLE<br>(See footnote **c**)
**Vomiting** *(despite optimal antiemetics)*<br>Grade 2	Maintain dose	↓ one dose level
Grades 3, 4	↓ one dose level	Discontinue
**Investigations (hepatic):**<br>Bilirubin, AST, alk phos<br>Grade 2	Maintain dose	*Hold until bilirubin returns to the* ***baseline grade*** *and AST and alk phos have returned to ≤ grade 1, then:*<br>↓ oxaliplatin *and* irinotecan one dose level
Grade 3	↓ one dose level	*Hold until bilirubin returns to the* ***baseline grade*** *and AST and alk phos have returned to ≤ grade 1, then:*<br>↓ two dose levels
Grade 4	Discontinue	Discontinue
**Febrile neutropenia:**<br>Grade 3	*Consider use of growth factors to avoid delay with subsequent cycles.*<br>↓ one dose level	*Consider use of growth factors to avoid delay with subsequent cycles.*<br>↓ one dose level
Grade 4	↓ one dose level	Discontinue
**Infection:**		

Grade 2	Maintain dose	Maintain dose
Grade 3	↓ one dose level	↓ one dose level
Grade 4	↓ one dose level	Discontinue
**Other clinically significant AEs:**[e]<br>Grade 2	Maintain dose	↓ one dose level
Grade 3	↓ one dose level	↓ one dose level
Grade 4	↓ one dose level	Discontinue

a Chemotherapy should not proceed with ≥ grade 3 anemia. Transfusion is acceptable for improving the hemoglobin value to allow therapy to continue without delay. The patient should be assessed to rule out other causes of anemia. *Use of erythropoiesis-stimulating agents is discouraged.*

b Resolved means that all clinically significant AEs are ≤ grade 1 (except bilirubin, which must be ≤ the baseline grade) on Day 1 of the next scheduled cycle (i.e., treatment can be given without delay).

c Hold and check weekly. ***With exception of bilirubin, resume treatment when toxicity is ≤ grade 1.*** If toxicity has not resolved after 4 weeks of delay, discontinue mFOLFIRINOX.

d If multiple episodes of grade 2 diarrhea occur during the cycle but do not delay the subsequent cycle, irinotecan may be decreased one dose level at the discretion of the investigator.

e Determination of "clinically significant" AEs is at the discretion of the investigator.

Table 8.2.3.2. Treatment management for mFOLFIRINOX (*continued*)

**Nervous System Disorders**		
**Paresthesias/Dysesthesias/ Neuropathy (Peripheral motor; Peripheral sensory)**	**1-7 day duration**<br>*(intermittent or continuous)*	**> 7 day duration**<br>*(intermittent or continuous)*
Grade 1	Maintain dose	Maintain dose
Grade 2	Maintain dose[a]	Decrease ***oxaliplatin*** one dose level[b]
Grade 3	**First episode**:<br>Decrease ***oxaliplatin*** one dose level[a]<br>**Second episode**:<br>Discontinue ***oxaliplatin***	Discontinue ***oxaliplatin***
Grade 4	Discontinue	
**Respiratory, thoracic and mediastinal disorders**		
**Laryngopharyngeal dysesthesia**	**1-7 day duration**<br>*(intermittent or continuous)*	**> 7 day duration**<br>*(intermittent or continuous)*

Grade 1<br>Grade 2	Maintain dose and increase infusion time of oxaliplatin to 6 hours	Maintain dose and increase infusion time of oxaliplatin to 6 hours
Grade 3	Increase duration of infusion to 6 hours	Discontinue oxaliplatin
Grade 4	Discontinue oxaliplatin	Discontinue oxaliplatin
**Respiratory, thoracic and mediastinal disorders**		
**Dyspnea** ≥ grade 2<br>**Hypoxia** ≥ grade 2<br>**Pneumonitis/pulmonary infiltrates** ≥ grade 2<br>**Pulmonary fibrosis** ≥ grade 2<br>**Cough** ≥ grade 3	Hold **all therapy** until interstitial lung disease is ruled out.<br>• If non-infectious interstitial lung disease is confirmed, oxaliplatin must be discontinued.<br>• If non-infectious interstitial disease is ruled out and infection (if any) has resolved, patients with persistent Grade 2 dyspnea or hypoxia can resume treatment at the discretion of the investigator.	
**a** Hold until improved to ***less than moderate symptom intensity*** on the next treatment day.<br>**b** ***Hold oxaliplatin for persistent moderate intensity grade 2 neuropathy***. When improved to ***less than moderate intensity***, resume treatment with dose modification for oxaliplatin. If ***moderate intensity Grade 2 toxicity*** persists after 4 weeks of delay, discontinue oxaliplatin. Continue 5-FU + LV and irinotecan while oxaliplatin is held.		

8.2.4 Treatment Management of Patients on Chemoradiation*

For management of capecitabine or 5-FU related toxicities, please see Table 8.2.2.2 (Section 8.2.2).

Table 8.2.4.1: Dose levels for capecitabine during chemoradiation

	Dose Level 0<br>Starting Dose (mg/m2/d)	Dose Level -1<br>(mg/m2/d)	Dose Level -2<br>(mg/m2/d)	Dose Level -3<br>(mg/m2/d)
Capecitabine	1650 per day in two divided doses on days of RT	1240 per day in two divided doses on days of RT	930 per day in two divided doses on days of RT	Discontinue

Table 8.2.4.2: Dose levels for 5-FU during chemoradiation

	Dose Level 0<br>Starting Dose (mg/m2)[a]	Dose Level -1<br>(mg/m2)[a]	Dose Level -2<br>(mg/m2)[a]	Dose Level -3<br>(mg/m2)
Infusional 5-FU	225 mg/m2 iv continuous infusion	170 mg/m2 iv continuous infusion	125 mg/m2 iv continuous infusion	Discontinue
a: continuous infusion Monday-Friday on radiation days or 7 days per week based on preference of the site				

***NOTE: If in the opinion of the investigator, a toxicity is considered to be due solely to the radiation, then the radiation can be held at the investigator's discretion. The rationale for treatment break and the duration of the**

break needs to be documented. Investigators are encouraged to reach out to Dr. William A. Hall and/or Dr. Paul B. Romesser to discuss concerns with radiation toxicity resulting in a treatment break.

8.2.5 Dose Modifications for Obese Patients

There is no clearly documented adverse impact of treatment of obese patients when dosing is performed according to actual body weight. Therefore, all dosing is to be determined solely by actual weight without any modification unless explicitly described in the protocol. This will eliminate the risk of calculation error and the possible introduction of variability in dose administration. Failure to use actual body weight in the calculation of drug dosages will be considered a major protocol deviation. Physicians who are uncomfortable with calculating doses based on actual body weight should recognize that doing otherwise would be a protocol violation. Physicians may consult the published guidelines of the American Society of Clinical Oncology Appropriate Chemotherapy Dosing for Obese Adult Patients with Cancer: American Society of Clinical Oncology Clinical Practice Guideline. J Clin Oncol 30(13): 1553-1561, 2012.

9.0 ADVERSE EVENTS

The prompt reporting of adverse events is the responsibility of each investigator engaged in clinical research, as required by Federal Regulations. Adverse events must be described and graded using the terminology and grading categories defined in the NCI's Common Terminology Criteria for Adverse Events (CTCAE), Version 5.0. The CTCAE is available at ctep.cancer.gov/protocolDevelopment/electronic_applications/ctc.htm. Attribution to protocol treatment for each adverse event must be determined by the investigator and reported on the required forms. Please refer the NCI Guidelines: Adverse Event Reporting Requirements for further details on AE reporting procedures.

9.1 Routine Adverse Event Reporting

Adverse event data collection and reporting, which are required as part of every clinical trial are done to ensure the safety of patients enrolled in the studies as well as those who will enroll in future studies using similar agents. Adverse events are reported in a routine manner at scheduled times according to the study calendar in Section 5.0. For this trial, the "Adverse Events" Form is used for routine AE reporting in Rave.

9.1.1 Solicited adverse events

The following adverse events are considered "expected" and their presence/absence should be solicited, and severity graded, at baseline and for each cycle of treatment.

CTCAE v5.0 Term	CTCAE v5.0 System Organ Class (SOC)
Nausea	Gastrointestinal disorders
Vomiting	Gastrointestinal disorders
Peripheral sensory neuropathy	Nervous system disorders
Diarrhea	Gastrointestinal disorders
Constipation	Gastrointestinal disorders
Mucositis oral	Gastrointestinal disorders

Fatigue	General disorders and administration site conditions
Pain	General disorders and administration site conditions
Anorexia	Metabolism and nutrition disorders
Rectal mucositis	Gastrointestinal disorders
Anal mucositis	Gastrointestinal disorders
Laryngopharyngeal dysesthesia	Respiratory, thoracic, and mediastinal disorders

9.2 CTCAE Routine Reporting Requirements

In addition to the solicited adverse events listed in Section 9.1, the following table outlines the combinations of time points, grades and attributions of AEs that require routine reporting to the Alliance Statistics and Data Center. Questions about routine reporting should be directed to the Data Manager.

Combinations of CTCAE Grade & Attribution Required for Routine AE Data Submission on Case Report Forms (CRFs)

Attribution	Grade 1	Grade 2	Grade 3	Grade 4	Grade 5
Unrelated			a	a	a
Unlikely			a	a	a
Possible		a	a, b	a, b	a, b
Probable		a	a, b	a, b	a, b
Definite		a	a, b	a, b	a, b

a) **Adverse Events CRF -** Applies to AEs occurring between registration and within 30 days of the patient's last treatment date.

b) **Adverse Events: Late CRF -** Applies to AEs occurring greater than 30 days after the patient's last treatment date, or as part of the Clinical Follow-up Phase or Survival Follow-up Phase.

9.3 Expedited Adverse Event Reporting (CTEP-AERS)

Investigators are required by Federal Regulations to report serious adverse events as defined in the table below. The descriptions and grading scales found in the NCI Common Terminology Criteria for Adverse Events (CTCAE) version 5 will be utilized for AE reporting. The CTCAE is identified and located on the CTEP website at: ctep.cancer.gov/protocolDevelopment/electronic_applications/ctc.htm. All appropriate treatment areas should have access to a copy of the CTCAE. All reactions determined to be "reportable" in an expedited manner must be reported using the Cancer Therapy Evaluation Program Adverse Event Reporting System (CTEP-AERS).

For further information on the NCI requirements for SAE reporting, please refer to the 'NCI Guidelines for Investigators: Adverse Event Reporting Requirements' document published by the NCI.

Note: All deaths on study require both routine and expedited reporting regardless of causality. Attribution to treatment or other cause should be provided.

9.3.1 Expedited reporting requirements for adverse events that occur within 30 days of the last administration of the agent/intervention

FDA REPORTING REQUIREMENTS FOR SERIOUS ADVERSE EVENTS (21 CFR Part 312)

NOTE: Investigators **MUST** immediately report to the sponsor (NCI) **ANY** Serious Adverse Events, whether or not they are considered related to the investigational agent(s)/intervention (21 CFR 312.64)

An adverse event is considered serious if it results in **ANY** of the following outcomes:

1) Death
2) A life-threatening adverse event
3) An adverse event that results in inpatient hospitalization or prolongation of existing hospitalization for ≥ 24 hours
4) A persistent or significant incapacity or substantial disruption of the ability to conduct normal life functions
5) A congenital anomaly/birth defect.
6) Important Medical Events (IME) that may not result in death, be life threatening, or require hospitalization may be considered serious when, based upon medical judgment, they may jeopardize the patient or subject and may require medical or surgical intervention to prevent one of the outcomes listed in this definition. (FDA, 21 CFR 312.32; ICH E2A and ICH E6).

ALL SERIOUS adverse events that meet the above criteria **MUST** be immediately reported to the NCI via electronic submission within the timeframes detailed in the table below.

Hospitalization	Grade 1 Timeframes	Grade 2 Timeframes	Grade 3 Timeframes	Grade 4 & 5 Timeframes
Resulting in Hospitalization ≥ 24 hrs	10 Calendar Days			24-Hour 5 Calendar Days
Not resulting in Hospitalization ≥ 24 hrs	Not required		10 Calendar Days	

NOTE: Protocol specific exceptions to expedited reporting of serious adverse events are found in the Specific Protocol Exceptions to Expedited Reporting (SPEER) portion of the CAEPR

Expedited AE reporting timelines are defined as:

- "24-Hour; 5 Calendar Days" - The AE must initially be submitted electronically within 24 hours of learning of the AE, followed by a complete expedited report within 5 calendar days of the initial 24-hour report.
- "10 Calendar Days" - A complete expedited report on the AE must be submitted electronically within 10 calendar days of learning of the AE.

[1]Serious adverse events that occur more than 30 days after the last administration of investigational agent/intervention and have an attribution of possible, probable, or definite require reporting as follows:

Expedited 24-hour notification followed by complete report within 5 calendar days for:

- All Grade 4, and Grade 5 AEs

Expedited 10 calendar day reports for:

- Grade 2 adverse events resulting in hospitalization or prolongation of hospitalization
- Grade 3 adverse events

[2]For studies using PET or SPECT IND agents, the AE reporting period is limited to 10 radioactive half-lives, rounded UP to the nearest whole day, after the agent/intervention was last administered. Footnote "1" above applies after this reporting period.

Effective Date: May 5, 2011

9.3.2 Expedited AE reporting timelines defined

- "24 hours; 5 calendar days" – The investigator must initially report the AE via CTEP-AERS ≤ 24 hours of learning of the event followed by a complete CTEP-AERS report ≤ 5 calendar days of the initial 24-hour report.
- "10 calendar days" - A complete CTEP-AERS report on the AE must be submitted ≤ 10 calendar days of the investigator learning of the event.

Any medical event equivalent to CTCAE grade 3, 4, or 5 that precipitates hospitalization (or prolongation of existing hospitalization) must be reported regardless of attribution and designation as expected or unexpected with the exception of any events identified as protocol-specific expedited adverse event reporting exclusions (see below).

Any event that results in persistent or significant disabilities/incapacities, congenital anomalies, or birth defects must be reported via CTEP-AERS if the event occurs following treatment with an agent under an IND.

Use the NCI protocol number and the protocol-specific patient ID provided during trial registration on all reports.

9.3.3 Additional Instructions or Exclusions to CTEP-AERS Expedited Reporting Requirements

All adverse events reported via CTEP-AERS (i.e., serious adverse events) should also be forwarded to your local IRB.

Treatment expected adverse events include those listed in Section 10.0 and in the package insert.

CTEP-AERS reports should be submitted electronically.

Exclusions

≤ grade 4 hematosuppression and hospitalization resulting from such do not require CTEP-AERS, but should be submitted as part of study results.

Grade 1-3 nausea or vomiting and hospitalization resulting from such do not require AERS reporting, but should be reported via routine AE reporting

Grade 3 nausea or vomiting does not require AERS reporting, but should be reported via routine AE reporting.

Death

Death due to progressive disease should be reported as Grade 5 "Disease progression" in the system organ class (SOC) "General disorders and administration site conditions." Evidence that the death was a manifestation of underlying disease (e.g., radiological changes suggesting tumor growth or progression: clinical deterioration associated with a disease process) should be submitted.

Any death occurring within 30 days of the last dose, regardless of attribution to the investigational agent/intervention requires expedited reporting within 24 hours.

Any death occurring greater than 30 days after the last dose of the investigational agent/intervention requires expedited reporting within 24 hours only if it is possibly, probably, or definitely related to the investigational agent/intervention.

Pregnancy loss and neonatal death

Pregnancy loss is defined in CTCAE as "Death in utero." Any Pregnancy loss should be reported expeditiously, as Grade 4 "Pregnancy loss" under the Pregnancy, puerperium and perinatal conditions SOC. A Pregnancy loss should NOT be reported as a Grade 5 event under the Pregnancy, puerperium and perinatal conditions SOC, as currently CTEP-AERS recognizes this event as a patient death.

A neonatal death should be reported expeditiously as Grade 4, "Death neonatal" under the General disorders and administration SOC.

New Malignancies

All new malignancies must be reported via CTEP-AERS whether or not they are thought to be related to either previous or current treatment. All new malignancies should be reported, i.e. solid tumors (including non-melanoma skin malignancies), hematologic malignancies, myelodysplastic syndrome/acute myelogenous leukemia, and in situ tumors.

Whenever possible, the CTEP-AERS reports for new malignancies should include tumor pathology, history or prior tumors, prior treatment/current treatment including duration, any associated risk factors or evidence regarding how long the new malignancy may have been present, when and how the new malignancy was detected, molecular characterization or cytogenetics of the original tumor (if available) and of any new tumor, and new malignancy treatment and outcome, if available.

Secondary Malignancy

A secondary malignancy is a cancer caused by treatment for a previous malignancy (e.g., treatment with investigational agent/intervention, radiation or chemotherapy). A secondary malignancy is not considered a metastasis of the initial neoplasm.

CTEP requires all secondary malignancies that occur following treatment with an agent under an NCI IND/IDE be reported via CTEP-AERS. Three options are available to describe the event:

- Leukemia secondary to oncology chemotherapy (e.g., acute myelocytic leukemia [AML])
- Myelodysplastic syndrome (MDS)
- Treatment-related secondary malignancy

Any malignancy possibly related to cancer treatment (including AML/MDS) should also be reported via Rave.

Second Malignancy

A second malignancy is one unrelated to the treatment of a prior malignancy (and is NOT a metastasis from the initial malignancy). Second malignancies require ONLY routine reporting unless otherwise specified.

10.0 DRUG INFORMATION

10.1 General Considerations:

The total administered dose of chemotherapy may be rounded up or down within a range of 10% of the actual calculated dose.

It is not necessary to change the doses of the study drugs due to changes in weight unless the calculated dose changes by ≥10%.

All study agents are to be administered at the registering institution.

If the Group credited for enrollment is a non-Alliance Group, then other requirements from the credited Group may apply.

10.2 Oxaliplatin (NSC #266046)

NOTE: Please refer to package insert for additional information.

Agent ordering and agent accountability

Institutional pharmacy shall obtain supplies from normal commercial supply chain or wholesaler.

Investigator Brochure Availability

Consult the package insert for the most current and complete information.

Formulation

Commercially available as:

- Solution for Injection: 50 mg/10 mL (10 mL), 100 mg/20 mL (20 mL), and 200 mg/40 mL (40 mL)
- Lyophilized Powder for Injection: 50 mg and 100 mg

Storage

Store intact vials in original outer carton at room temperature and; do not freeze.

Stability

According to the manufacturer, solutions diluted for infusion are stable up to 6 hours at room temperature or up to 24 hours under refrigeration. Oxaliplatin solution diluted with D5W to a final concentration of 0.7 mg/mL (polyolefin container) has been shown to retain >90% of its original concentration for up to 30 days when stored at room temperature or refrigerated; artificial light did not affect the concentration (Andre, 2007). As this study did not examine sterility, refrigeration would be preferred to limit microbial growth. Infusion solutions do not require protection from light.

Preparation

Dilution with D5W (250 or 500 mL) is required prior to administration. Do not prepare using a chloride-containing solution (e.g. NaCl). Refer to package insert for complete preparation and dispensing instructions.

Administration

Refer to the treatment section for specific administration instructions. Administer as IV infusion over 2-6 hours. Flush infusion line with D5W prior to administration of any concomitant medication. Patients should receive an antiemetic premedication regimen. Cold temperature may exacerbate acute neuropathy. Avoid mucositis prophylaxis with ice chips during oxaliplatin infusion.

Drug Interactions

Concurrent use of oxaliplatin and QT prolonging drugs may result in increased risk of QT-interval prolongation; use caution during coadministration. Please consult an up-to-date drug reference for additional drug interaction information.

Pharmacokinetics

Distribution: Vd: 440 L

Protein binding: >90% primarily albumin and gamma globulin (irreversible binding to platinum)

Metabolism: Nonenzymatic (rapid and extensive), forms active and inactive derivatives

Half-life: 392 hours

Excretion: Primarily urine (~54%); feces (~2%)

Adverse Events

Consult the package insert for the most current and complete information. Percentages reported with monotherapy.

Common known potential toxicities, > 10%:

Central nervous system: Fatigue, fever, pain, headache, insomnia

Gastrointestinal: Nausea, diarrhea, vomiting, abdominal pain, constipation, anorexia, stomatitis

Hematologic: Anemia, thrombocytopenia, leukopenia

Hepatic: Liver enzymes increased

Neuromuscular & skeletal: Back pain, peripheral neuropathy (may be dose limiting). The most commonly observed oxaliplatin toxicity is acute and cumulative neurotoxicity, observed in patients treated at doses above 100 mg/m2/cycle. This neurotoxicity has included paresthesias and dysesthesias of the hands, feet, and perioral region as well as unusual laryngopharyngeal dysesthesias characterized by a loss of sensation of breathing without any objective evidence of respiratory distress (hypoxia, laryngospasm, or bronchospasm). Oxaliplatin neurotoxicity appears to be exacerbated by exposure to cold. Patients on this study will be counseled to avoid cold drinks and exposure to cold water or air. Should a patient develop laryngopharyngeal dysesthesia, their oxygen saturation should be evaluated via a pulse oximeter; if normal, an anxiolytic agent should be given and the patient observed in the clinic until the episode has resolved. Because this syndrome may be associated with the rapidity of oxaliplatin infusion, subsequent doses of oxaliplatin should be administered as a 6-hour infusion (instead of the normal 2-hour infusion).

Acute and cumulative neurotoxicities are dose limiting for oxaliplatin. The acute neurotoxicity is characterized by paresthesias and dysesthesias that may be triggered or exacerbated by exposure to cold. These symptoms occur within hours of exposure and are usually reversible over the following hours or days. Cumulative doses of oxaliplatin above 680 mg/m2 may produce functional coordination; impairment is caused by sensory rather than motor changes.

The likelihood of experiencing neurotoxicity is directly related to the total cumulative dose of oxaliplatin administered. The relative risk of developing neurotoxicity was 10%, 50%, and 75% in patients who received total cumulative oxaliplatin doses of 780 mg/m2, 1,170 mg/m2, and 1,560 mg/m2, respectively. Both acute and cumulative neurotoxicities due to oxaliplatin have lessened in 82% of patients within 4 to 6 months, and have completely disappeared by 6 to 8 months in 41% of patients. In addition, the likelihood that neurologic symptoms will regress has been shown to correlate inversely with cumulative dose.

Respiratory: Dyspnea, cough

Less common known potential toxicities, 1% - 10%:

Cardiovascular: Edema, chest pain, peripheral edema, flushing, thromboembolism

Central nervous system: Dizziness

Dermatologic: Rash, alopecia, hand-foot syndrome

Endocrine & metabolic: Dehydration, hypokalemia

Gastrointestinal: Dyspepsia, taste perversion, flatulence, mucositis, gastroesophageal reflux, dysphagia

Genitourinary: Dysuria

Hematologic: Neutropenia

Local: Injection site reaction

Neuromuscular & skeletal: Rigors, arthralgia

Ocular: Abnormal lacrimation

Renal: Serum creatinine increased

Respiratory: URI, rhinitis, epistaxis, pharyngitis, pharyngolaryngeal dysesthesia

Miscellaneous: Allergic reactions, hypersensitivity (includes urticaria, pruritus, facial flushing, shortness of breath, bronchospasm, diaphoresis, hypotension, syncope, hiccup

Rare known potential toxicities, <1% (Limited to important or life-threatening):

Gastrointestinal: Life threatening enteric sepsis secondary to neutropenia and diarrhea.

Hepatic: Veno-occlusive disease of the liver is a rare serious adverse event that has occurred in association with administration of oxaliplatin and fluorouracil.

Otic: Clinical ototoxicity occurs in less than 1% of patients following oxaliplatin administration, and sever ototoxicity has not been reported

Nursing Guidelines

- GI toxicity similar to cisplatin occurs with doses above 30 mg/m^2. It can be almost constant and frequently severe, but not always dose-limiting. Monitor for nausea and vomiting and treat accordingly.
- Dose-limiting side effect can be paresthesias of hands, fingers, toes, pharynx, and occasionally cramps which develops with a dose-related frequency (>90 mg/m^2). Duration of symptoms tend to be brief (less than a week) with the first course, but longer with subsequent courses. Phase I patients have reported exacerbation of paresthesias by touching cold surfaces or exposure to cold. Advise patient of these possibilities and instruct patient to report these symptoms to the health care team. Also advise patient to refrain from operating dangerous machinery that requires fine sensory-motor coordination, if symptoms appear.
- These sensory neuropathies developed after subsequent courses with increasing intensity (Grade 3 toxicity after the fourth course) and with increasing duration. In 63% of the patients tested in phase I at high doses (135-200 mg/m^2), neuropathies became long-term with slow reversal over several months. Disabling walking and handwriting difficulties, as well as mouth and throat dysesthesias and laryngospasms were seen. Instruct patient to report any swallowing difficulties or gait changes.
- Oxaliplatin is incompatible with NS. Flush lines with D5W prior to and following oxaliplatin infusion.

- Low back pain is a common side effect, perhaps a form of hypersensitivity reaction. Instruct patient in good body mechanics, advise light massage, heat, etc.
- Laryngopharyngeal dysesthesia (LPD) occurs in about 15% of patients and is acute, sporadic, and self-limited. It usually occurs within hours of infusion, is induced or exacerbated by exposure to cold, and presents with dyspnea and dysphagia. The incidence and severity appear to be reduced by prolonging infusion time. Instruct patient to avoid ice and cold drinks the day of infusion. If ≥Grade 2 laryngopharyngeal dysesthesia occurs during the administration of oxaliplatin, do the following:
  - Stop oxaliplatin infusion
  - Administer benzodiazepine and give patient reassurance
  - Test oxygen saturation via a pulse oximeter
  - At the discretion of the investigator, the infusion can be restarted at 1/3 the original rate of infusion.
  - Rapid resolution is typical within minutes to a few hours. Can recur with retreatment.

**Comparison of the Symptoms and Treatment of Laryngopharyngeal Dysesthesias and Platinum Hypersensitivity Reactions**		
**Clinical Symptoms**	**Laryngopharyngeal Dysesthesias**	**Platinum Hypersensitivity**
dyspnea	present	present
bronchospasm	absent	present
laryngospasm	absent	present
anxiety	present	present
O_2 saturation	normal	decreased
difficulty swallowing	present (loss of sensation)	absent
pruritus	absent	present
urticaria/rash	absent	present
cold-induced symptoms	yes	no
BP	normal or increased	normal or decreased
**Treatment**	anxiolytics, observation in a controlled clinical setting until symptoms abate or at the physicians' discretion	oxygen, steroids, epinephrine, bronchodilators; fluids and vasopressors, if appropriate

- Alopecia is rare with oxaliplatin alone, but is seen with 5-FU-oxaliplatin combination. Advise patient.

- Mild-moderate diarrhea has been seen -- usually of short duration. Treat accordingly. See Section 8.1 for ancillary treatment.
- Respiratory problems (i.e., pulmonary fibrosis, cough, dyspnea, rales, pulmonary infiltrates, hypoxia, air hunger and tachypnea) have been observed in patients administered oxaliplatin. In rare cases, death has occurred due to pulmonary fibrosis. Please monitor and instruct the patient to report any respiratory difficulties and hold oxaliplatin until interstitial lung disease is ruled out for cases of Grade ≥3. If patient is experiencing shortness of breath, a chest x-ray and assessment of oxygenation via either finger oximetry or arterial blood gas evaluation are required to confirm the absence or presence of pulmonary infiltrates and/or hypoxia (treat accordingly: no intervention, steroids, diuretics, oxygen, or assisted ventilation).
- Veno-occlusive disease (VOD) is a rare but serious complication that has been reported in patients receiving oxaliplatin in combination with 5-FU. This condition can lead to hepatomegaly, splenomegaly, portal hypertension and/or esophageal varices. Instruct patients to report any jaundice, ascites, or hematemesis to the MD immediately as these could be a sign of VOD or other serious condition.
- Acute vein irritation can occur with infusion. Apply heat to arm of infusion if you are using a peripheral line. However, extravasation of drug can cause severe pain, redness, soreness, and exfoliation of the skin in the affected area with loss of affected vein for a long period. If a patient has a problem with pain or sclerosis when chemotherapy is given by a peripheral line, then placement of a central line should be considered.
- Hemolytic Uremic Syndrome (HUS) may result in kidney damage. Oxaliplatin is to be discontinued in cases where hematocrit is <25%, thrombocytopenia <100,000, and creatinine ≥1.6 mg/dL.
- Patients may experience sleep disturbances, specifically insomnia. Encourage good sleep hygiene, and instruct patient to report any problems with sleep to the MD, to assess for the potential use of sleeping aids.
- Cold-induced transient visual abnormalities can be experienced by patients while receiving oxaliplatin, although the relationship to oxaliplatin has not been completely determined. Instruct patient to report any problems with vision to the MD.
- Extrapyramidal side effects and/or involuntary limb movement has been seen with oxaliplatin administration. Patients may also experience restlessness. Instruct patient to report any of these side effects to the MD.

10.3 5-Fluorouracil (NSC# 19893)

NOTE: Please refer to package insert for additional information.

Agent ordering and agent accountability

Institutional pharmacy shall obtain supplies from normal commercial supply chain or wholesaler.

Investigator Brochure Availability

Consult the package insert for the most current and complete information.

Formulation

Commercially available as Intravenous Solution: 500 mg/10 mL (10 mL), 1 g/20 mL (20 mL), 2.5 g/50 mL (50 mL), and 5 g/100 mL (100 mL)

Storage

Store intact vials at room temperature and protect from light.

Stability

A slight discoloration may occur with storage but usually does not denote decomposition. Solutions in 50 – 1000 mL 0.9% NaCl or D5W or undiluted solutions in syringes are stable for 72 hours at room temperature.

Preparation

Dilute in 50 – 1000 mL of 0.9% NaCl or D5W. If exposed to cold, a precipitate may form; gentle heating to 60ºC will dissolve the precipitate without impairing the potency.

Administration

Fluorouracil may be given IV push, IV infusion. Refer to the treatment section for specific administration instructions. Avoid extravasation, may be an irritant.

Drug Interactions

Fluorouracil may increase effects of warfarin. Avoid ethanol (due to GI irritation). Avoid black cohosh.

Pharmacokinetics

Distribution: Vd ~ 22% of total body water; penetrates extracellular fluid, CSF, and third space fluids (e.g. pleural effusions and ascitic fluid)

Metabolism: Hepatic (90%); via a dehydrogenase enzyme; Fluorouracil must be metabolized to be active.

Half-life elimination: Biphasic: Initial: 6-20 minutes; two metabolites, FdUMP and FUTP, have prolonged half-lives depending on the type of tissue.

Excretion: Lung (large amounts as CO2); urine (5% as unchanged drug) in 6 hours.

Adverse Events

Consult the package insert for the most current and complete information.

Common known potential toxicities, > 10%:

Dermatologic: Dermatitis, pruritic maculopapular rash, alopecia.

Gastrointestinal (route and schedule dependent): Heartburn, nausea, vomiting, anorexia, stomatitis, esophagitis, anorexia, diarrhea. GI toxicity (anorexia, nausea, and vomiting) is generally more severe with continuous-infusion schedules.

Emetic potential: <1000 mg: Moderately low (10% to 30%) ≥ 1000 mg: Moderate (30% to 60%)

Hematologic: Leukopenia; Myelosuppressive (tends to be more pronounced in patients receiving bolus dosing of FU). Decreased white blood cell count with increased risk of infection; decreased platelet count with increased risk of bleeding.

Local: Irritant chemotherapy.

Less common known potential toxicities, 1% - 10%:

Dermatologic: Dry skin

Gastrointestinal: GI ulceration

Rare known potential toxicities, <1% (Limited to important or life-threatening):

Cardiac enzyme abnormalities, chest pain, coagulopathy, dyspnea, ECG changes similar to ischemic changes, hepatotoxicity; hyperpigmentation of nail beds, face, hands, and veins used in infusion; hypotension, palmar-plantar syndrome (hand-foot syndrome), photosensitization. Cerebellar ataxia, headache, somnolence, ataxia are seen primarily in intracarotid arterial infusions for head and neck tumors.

Nursing Guidelines

- Monitor complete blood count and platelet count. Instruct patient to report signs and symptoms of infection, unusual bruising or bleeding to the physician.
- Administer antiemetics as indicated.
- Diarrhea may be dose–limiting; encourage fluids and treat symptomatically.
- Assess for stomatitis; oral care measures as indicated. May try vitamin E oil dabbed on sore, six times daily. Cryotherapy recommended with IV push administration.
- Monitor for neurologic symptoms (headache, ataxia).
- Inform patient of potential alopecia.
- Those patients on continuous infusion may need instruction regarding central intravenous catheters and portable intravenous or IA infusion devices.
- 5FU–induced conjunctivitis is a common problem. Advise patient to report any eye soreness or redness to the healthcare team.
- Photosensitivity may occur. Instruct patients to wear sun block when outdoors.

10.4 Leucovorin calcium (NSC# 3590)

NOTE: Please refer to package insert for additional information.

Agent ordering and agent accountability

Institutional pharmacy shall obtain supplies from normal commercial supply chain or wholesaler.

Investigator Brochure Availability

Consult the package insert for the most current and complete information.

Formulation

Commercially available as:

• Solution for Injection: 100 mg/10mL (10mL, 30 mL)

• Lyophilized Powder for Injection: 50 mg, 100 mg, 200 mg, 350 mg, and 500 mg

Storage

Solution for Injection: Prior to dilution, store vials under refrigeration at 2°C to 8°C (36°F to 46°F). Protect from light.

Powder for Injection: Store at room temperature of 25°C (77°F). Protect from light

Stability

Solutions reconstituted with bacteriostatic water for injection U.S.P. must be used within 7 days. Solutions reconstituted with SWFI must be used immediately. Parenteral admixture is stable for

24 hours stored at room temperature (25°C) and for 4 days when stored under refrigeration (4°C).

Preparation

Solution for Injection: Dilute in D5W or NS for infusion.

Powder for Injection: Reconstitute with SWFI or BWFI; dilute with D5W or NS for infusion. When doses >10 mg/m2 are required, reconstitute using sterile water for injection, not a solution containing benzyl alcohol.

Administration

Combination therapy with Fluorouracil: Fluorouracil is usually given after, or at the midpoint, of the leucovorin infusion. Leucovorin is usually administered by IV bolus injection or short (10-120 minutes) IV infusion. Other administration schedules have been used; refer to the treatment section for specific administration instructions.

Drug Interactions

Fluorouracil (Systemic): Leucovorin Calcium-Levoleucovorin may enhance the adverse/toxic effect of Fluorouracil (Systemic). This effect is associated with the ability of leucovorin or levoleucovorin to enhance the anticancer effects of fluorouracil. Risk C: Monitor therapy

Fosphenytoin: Leucovorin Calcium-Levoleucovorin may decrease the serum concentration of Fosphenytoin. Risk C: Monitor therapy

Phenobarbital: Leucovorin Calcium-Levoleucovorin may decrease the serum concentration of Phenobarbital. Risk C: Monitor therapy

Phenytoin: Leucovorin Calcium-Levoleucovorin may decrease the serum concentration of Phenytoin. Risk C: Monitor therapy

Primidone: Leucovorin Calcium-Levoleucovorin may decrease the serum concentration of Primidone. Additionally, leucovorin/levoleucovorin may decrease concentrations of active metabolites of primidone (e.g., phenobarbital). Risk C: Monitor therapy

Raltitrexed: Leucovorin Calcium-Levoleucovorin may diminish the therapeutic effect of Raltitrexed. Risk X: Avoid combination

Trimethoprim: Leucovorin Calcium-Levoleucovorin may diminish the therapeutic effect of Trimethoprim. Management: Avoid concurrent use of leucovorin or levoleucovorin with trimethoprim (plus sulfamethoxazole) for Pneumocystis jiroveci pneumonia. If trimethoprim is used for another indication, monitor closely for reduced efficacy. Risk X: Avoid combination.

Pharmacokinetics

Metabolism: Intestinal mucosa and hepatically to 5-methyl-tetrahydrofolate (5MTHF; active)

Half-life elimination: ~ 4-8 hours

Time to peak: IV: Total folates: 10 minutes; 5MTHF: ~ 1 hour

Excretion: Urine (primarily); feces

Adverse Events

Consult the package insert for the most current and complete information.

Dermatologic: Rash, pruritus, erythema, urticaria

Hematologic: Thrombocytosis

Respiratory: Wheezing

Miscellaneous: Allergic reactions, anaphylactoid reactions

Nursing Guidelines

- Headache may occur. Advise patient that analgesics such as Tylenol may help. Instruct patient to report any headache that is unrelieved.
- Observe for sensitization reaction (rash, hives, pruritis, facial flushing, and wheezing).
- May potentiate the toxic effects of fluropyrimidine (5-FU) therapy, resulting in increased hematologic and gastrointestinal (diarrhea, stomatitis) adverse effects. Monitor closely.
- May cause mild nausea or upset stomach. Administer antiemetics if necessary and evaluate for their effectiveness.

10.5 Capecitabine (NSC #712807)

NOTE: Please refer to package insert for additional information.

Agent ordering and agent accountability

Institutional pharmacy shall obtain supplies from normal commercial supply chain or wholesaler.

Investigator Brochure Availability

Consult the package insert for the most current and complete information.

Formulation

Oral tablets provided as 150 mg and 500 mg

Storage and Stability

Store at 25°C (77°F); excursions are permitted between 15°C and 30°C (59°F and 86°F). Keep bottle tightly closed.

Administration

Usually administered in 2 divided doses (in the morning and evening). Doses should be administered with water within 30 minutes after a meal. Swallow tablets whole. Avoid cutting or crushing tablets. Refer to the treatment section for specific administration instructions.

Drug Interactions

•Anticoagulants: Monitor anticoagulant response (INR or prothrombin time) frequently in order to adjust the anticoagulant dose as needed.

• Allopurinol: Avoid the use of allopurinol during treatment with XELODA.

• Food reduced both the rate and extent of absorption of capecitabine.

•The use of Sorivudine or its analogue, Birivudine, is contraindicated for this study due to a possible, even fatal, drug reaction. Assess patient's drug use. Impress on patients the importance of avoiding these drugs while on study.

•Monitor patient closely who are taking concomitant phenytoin therapy. There have been reports of increased levels of phenytoin in patients who are also taking capecitabine. These patients may require more frequent monitoring of their phenytoin levels and dose adjustments as necessary.

•Cimetidine may alter the clearance of capecitabine and cause toxic levels. Cimetidine should be avoided while taking capecitabine.

Pharmacokinetics

Following oral administration of 1255 mg/m2 BID to cancer patients, capecitabine reached peak blood levels in about 1.5 hours (Tmax) with peak 5-FU levels occurring slightly later, at 2 hours.

Food reduced both the rate and extent of absorption of capecitabine with mean Cmax and AUC0-∞ decreased by 60% and 35%, respectively. The Cmax and AUC0-∞ of 5-FU were also reduced by food by 43% and 21%, respectively. Food delayed Tmax of both parent and 5-FU by 1.5 hours

Adverse Events

Consult the package insert for the most current and complete information.

Most common adverse reactions (≥30%) were diarrhea, hand-and-foot syndrome, nausea, vomiting, abdominal pain, fatigue/weakness, and hyperbilirubinemia. Other adverse reactions, including serious adverse reactions, have been reported.

Nursing Guidelines

- Instruct patients to take the tablets within 30 minutes of a meal (breakfast and dinner). Tablets should be swallowed with 6-8 oz. of water.
- Instruct patient to avoid taking a missed dose, to never double up on a dose, and to notify the health care team if a dose has been missed.
- Diarrhea can be severe and dose-limiting. Instruct patient to contact the health care team immediately if they experience >4 BMs/day and/or nocturnal diarrhea above baseline. Monitor carefully for dehydration and need for fluid and electrolyte replacement. Standard antidiarrheal treatment, e.g., loperamide is recommended.
- Nausea and vomiting can be severe and dose-limiting. Instruct patient to report nausea and vomiting to the health care team if they experience >2 episodes of emesis in a 24-hour period. Initiate symptomatic treatment.
- Hand and Foot Syndrome is common and dose-limiting (redness, swelling, pain, numbness, tingling, blistering, and moist desquamation). Instruct patient to notify health care team immediately if symptoms appear. Chemotherapy may have to be discontinued until symptoms subside with future dose reduction initiated. The syndrome may recur with a rechallenge.
    - Advise patient to apply cool compress for comfort.
    - Advise patient to avoid harsh soaps and to use alcohol-free emollients.
    - Administer analgesics as prescribed.
    - Administer systemic steroids and pyridoxine as prescribed.
- Treat stomatitis symptomatically -- may try dabbing vitamin E oil on lesions. Do not swallow oil. Advise frequent and careful oral hygiene.
- Assess for warfarin use. Patients taking coumadin-derivative anticoagulants concomitantly with capecitabine should be monitored regularly for alterations in their coagulation parameters (PT or INR).

- Renal impairment: Check creatinine values weekly and calculate creatinine clearance weekly for signs of renal impairment (if this is part of test schedule!!). Follow dose modifications.
- The use of Sorivudine or its analogue, Birivudine, is contraindicated for this study due to a possible, even fatal, drug reaction. Assess patient's drug use. Impress on patients the importance of avoiding these drugs while on study.
- Cardiotoxicity (including MI, angina, dysrhythmias, and cardiac arrest) has been seen with capecitabine. Observe patients closely for signs of cardiac dysfunction. Instruct patient to report any chest pain or palpitations to the health care team immediately or seek emergency medical attention.
- Monitor patient closely who are taking concomitant phenytoin therapy. There have been reports of increased levels of phenytoin in patients who are also taking capecitabine. These patients may require more frequent monitoring of their phenytoin levels and dose adjustments as necessary.
- Cimetidine may alter the clearance of capecitabine and cause toxic levels. Cimetidine should be avoided while taking capecitabine.

10.6 Irinotecan (NSC# 616348)

NOTE: Please refer to package insert for additional information.

Agent ordering and agent accountability

Institutional pharmacy shall obtain supplies from normal commercial supply chain or wholesaler.

Investigator Brochure Availability

Consult the package insert for the most current and complete information.

Formulation

Commercially available as: injection 20 mg/mL (2 mL, 5 mL, 15 mL) [contains sorbitol 45 mg/mL; do not use in patients with hereditary fructose intolerance].

Storage

Store intact vials at room temperature and protect from light.

Stability

Prepare the infusion solution immediately prior to use and commence infusion as soon as possible after preparation. If visible particulates are present in the infusion solution discard. If it is not possible to use the infusion solution immediately, the infusion solution may be stored for up to 24 hours at 2 °C to 8 °C or discarded.

Preparation

Doses should be diluted in 250-500 mL D5W or 0.9% NaCl to a final concentration of 0.12-2.8 mg/mL. Refer to package insert for complete preparation and dispensing instructions.

Administration

Administer by IV infusion, usually over 90 minutes. Refer to the treatment section for specific administration instructions.

Drug Interactions

Cytochrome P450 Effect: Substrate (major) of CYP2B6, 3A4

Increased Effect/Toxicity: CYP2B6 and CYP3A4 inhibitors may increase the levels/effects of irinotecan. Bevacizumab may increase the adverse effects of irinotecan (e.g. diarrhea, neutropenia). Ketoconazole increases the levels/effects of irinotecan and active metabolite; discontinue ketoconazole 1 week prior to irinotecan therapy; concurrent use is contraindicated.

Decreased Effect: CYP2B6 and CYP3A4 inducers may decrease the levels/effects of irinotecan.

Ethanol/Nutrition/Herb Interactions Herb/Nutraceutical: St. John's Wort decreases therapeutic effect of irinotecan; discontinue ≥ weeks prior to irinotecan therapy; concurrent use is contraindicated.

Pharmacokinetics

Distribution: Vd: 33-150 L/m2

Protein binding, plasma: Predominantly albumin; Parent drug: 30% to 68%, SN-38 (active metabolite): ~95%

Metabolism: Primarily hepatic to SN-38 (active metabolite) by carboxylesterase enzymes; SN-38 undergoes conjugation by UDP- glucuronosyl transferase 1A1 (UGT1A1) to form a glucuronide metabolite. Conversion of irinotecan to SN-38 is decreased and glucuronidation of SN-38 is increased in patients who smoke cigarettes, resulting in lower levels of the metabolite and overall decreased systemic exposure. SN-38 is increased by UGT1A1*28 polymorphism (10% of North Americans are homozygous for UGT1A1*28 allele). Patients homozygous for the UGT1A1*28 allele are at increased risk of neutropenia; initial one-level dose reduction should be considered for both single-agent and combination regimens. The lactones of both Irinotecan and SN-38 undergo hydrolysis to inactive hydroxyl acid forms.

Half-life elimination: SN-38: Mean terminal: 10-20 hours

Time to peak: SN-38: Following 90-minute infusion: ~1 hour

Excretion: Within 24 hours: urine: Irinotecan (11% to 20%), metabolites (SN-38 < 1%, SN-38 glucuronide, 3%)

Adverse Events

Consult the package insert for the most current and complete information.

Common known potential toxicities, > 10%:

Cardiovascular: Vasodilation

Central nervous system: Cholinergic toxicity (includes rhinitis, increased salivation, miosis, lacrimation, diaphoresis, flushing and intestinal hyperperistalsis); fever, pain, dizziness, insomnia, headache, chills

Dermatologic: Alopecia, rash

Endocrine & metabolic: Dehydration

Gastrointestinal: Late onset diarrhea, early onset diarrhea, nausea, abdominal pain, vomiting, cramps, anorexia, constipation, mucositis, weight loss, flatulence, stomatitis

Hematologic: Anemia, leukopenia, thrombocytopenia, neutropenia

Hepatic: Bilirubin increased, alkaline phosphatase increased

Neuromuscular & skeletal: Weakness, back pain

Respiratory: Dyspnea, cough, rhinitis

Miscellaneous: Diaphoresis, infection

Less common known potential toxicities, 1% - 10%:

Cardiovascular: Edema, hypotension, thromboembolic events

Central nervous system: Somnolence, confusion

Gastrointestinal: Abdominal fullness, dyspepsia

Hematologic: Neutropenic fever, hemorrhage, neutropenic infection

Hepatic: AST increased, ascites and/or jaundice

Respiratory: Pneumonia

Rare known potential toxicities, <1% (Limited to important or life-threatening):

ALT increased, amylase increased, anaphylactoid reaction, anaphylaxis, angina, arterial thrombosis, bleeding, Bradycardia, cardiac arrest, cerebral infarct, cerebrovascular accident, circulatory failure, colitis, deep thrombophlebitis, dysrhythmia, embolus, gastrointestinal bleeding, gastrointestinal obstruction, hepatomegaly, hiccups, hyperglycemia, hypersensitivity, hyponatremia, ileus, interstitial lung disease, intestinal perforation, ischemic colitis, lipase increased, lymphocytopenia, megacolon, MI, muscle cramps, myocardial ischemia, pancreatitis, paresthesia, peripheral vascular disorder, pulmonary embolus, pulmonary toxicity (dyspnea, fever, reticulonodular infiltrates on chest x-ray), renal failure (acute), renal impairment, syncope, thrombophlebitis, thrombosis, typhlitis, ulceration, ulcerative colitis, vertigo

Nursing Guidelines

- Cholinergic symptoms of lacrimation, nasal congestion, diaphoresis, flushing, ABD cramping, and diarrhea can occur at the beginning, during, or immediately after the irinotecan infusion. It is suggested that the patient remain in the treatment area for a minimum of one hour following the completion of the **very first** irinotecan infusion.
- Patient education is extremely important. Impress on the patient the importance of compliance with treatment of diarrhea management. Stress the need for prompt recognition and early intervention. Motivate the patient to report any complications immediately. The cholera-like syndrome can be unresponsive to conventional antidiarrheals and can result in severe dehydration.
- Ondanestron and diphenhydramine should provide good relief from the nausea/vomiting/cramping. Avoid prochlorperazine on the day of treatment due to its association with akathisia (motor restlessness). Prochlorperazine may be taken between treatments.
- Advise avoidance of excess caffeine, a GI stimulant. Avoid magnesium-based antacids such as Mylanta, Maalox, Rolaids, MOM, Mag-Ox 400, and Tylenol with antacid.
- The pulmonary toxicity seen is usually manifested by dyspnea beginning 42-175 days after treatment and occurs at a cumulative dose ranging from 400-1000 mg/m (median 750). Instruct patient to report any cough or SOB.
- Patients are at risk for developing eosinophilia and will improve on steroid therapy.
- Hepatic enzyme elevations have been transient and did not require intervention.

- Monitor CBC closely. Leukopenia occurs primarily as neutropenia but can be severe and dose limiting. The simultaneous occurrence of grade 4 diarrhea and grade 4 neutropenia is rare but may render the patient more susceptible to polymicrobial sepsis and potentially death.
- Advise patients of probable hair loss.

11.0 MEASUREMENT OF EFFECT

11.1 Radiological Tumor Evaluation

Standard and DW-MRI sequences will be obtained in 1.5T or 3T units by using a phased-array body coil. All imaging studies will be interpreted by expert radiology staff at the patient primary treatment center for patient eligibility, clinical staging, and tumor response, according to standard clinical criteria (see **Table 11.2.1** below). Central radiology review will not be required. Baseline images should be submitted to IROC within 30 business days of the exam with report. For restaging evaluation exams, images should be submitted within 30 business days of the exams with reports. Submitted reports should indicate N staging. N staging of N0 or N+ are accepted. Discs will be submitted de-identified and in the DICOM format. All patient identifiers must be removed and the unique case number included. As some patients who achieve a clinical complete response develop a local regrowth during surveillance, we have provided MRI features associated with local re-growth. These features are listed below in **Table 11.2.3**.

11.2 Clinical Tumor Evaluation prior to TME

Endoscopic exam: The length of the tumor is defined as the difference between the distance of the proximal and distal margins in relation to the anal verge. This is best determined by flexible sigmoidoscopy. Determination of clinical complete response, near complete response and incomplete response are determined as noted in Table 11.2.1 below.

For patients who undergo TME after TNT, the clinical tumor response evaluations occur prior to determination of need for TME (residual tumor/incomplete response) and will be performed by endoscopy, MRI and digital rectal exam. For patients who elect to undergo a WW approach, the clinical tumor evaluations will be performed by endoscopy, MRI, and digital rectal exam during the post-TNT follow-up and up to 5 years after randomization or up to salvage TME, whichever occurs first. Patients will be evaluated as per the schedule of tests noted in Section 5.0.

<u>Guidelines for Evaluation of Clinically Evaluable Disease</u>

Measurement Methods:

- All measurements should be recorded in metric notation (i.e., decimal fractions of centimeters) using a ruler or calipers.
- The same method of assessment must be used to characterize each identified and reported lesion at baseline and at the follow up evaluation.
- Flexible sigmoidoscopy with direct visualization is the accepted clinical evaluation along with digital rectal exam. Capturing the images in the electronic medical record is highly encouraged.
- Virtual colonoscopic assessments are not acceptable.

Table 11.2.1. Clinical and Radiologic Assessment:

Please see CTSU website for relevant forms for MRI and clinical/endoscopic response.

Please see CTSU website for endoscopic and MRI assessment of response video by Drs. Smith and Horvat. Please contact Drs. Horvat and/or Smith with questions about this section (see face page for contact information). [Alliance A022104 (JANUS) Trial Webinar - October 2023 (youtube.com)]

**MSK Regression Schema**[#]			
	**Clinical Complete Response**	**Near Complete Response**	**Incomplete / No Response**
**Endoscopy**	• Flat, white scar<br>• Telangiectasia<br>• No ulcer<br>• No nodularity	• Irregular mucosa<br>• Superficial ulceration<br>• Mild persisting erythema of the scar	• Visible tumor
**Digital Rectal Exam**	• Normal^	• Smooth induration or minor mucosal irregularity^	• Palpable tumor nodules^
**MRI-T2WI**	• Normal appearing rectal wall<br>OR<br>• Only fibrosis (dark T2 signal) and no intermediate signal intensity at the site of tumor[*]<br>AND<br>• No suspicious lymph nodes (Table Y)	• Predominantly fibrosis at the site of tumor[*] with punctate areas of T2 intermediate signal<br>AND/OR<br>• No suspicious or borderline enlarged lymph nodes (Table Y)	• Predominantly residual tumor with T2 intermediate signal and no or minimal fibrosis at the site of tumor[*]<br>AND/OR<br>• Suspicious lymph nodes (Table Y)<br>AND/OR<br>• Mucin at the site of tumor[***]<br>•
**MRI-DW**	• No restricted diffusion[**] at the site of tumor[*]	• Punctate areas of restricted diffusion[**] at the site of tumor[*]	• Restricted diffusion[**] at the site of tumor[*]

[*] Site of tumor: rectal wall, extramural vascular invasion and/or tumor deposit.

[**] Restricted diffusion: high signal on DWI high b-value (minimum b800) and low signal on ADC map.

T2 dark through (low signal on both DWI and ADC map) and *T2 shine thought* (high signal on both DWI and ADC map) effects are not considered restricted diffusion.

[***] MRI is unable to differentiate cellular from acellular mucin.

Clinicians can also refer to the following website for examples of cCR, nCR, and iCR: https://nomtrial.mskcc.org/Home/Index

^ Note not all tumors can be palpated (e.g., 10-11 cm from the anal verge) and thus the endoscopic features will be paramount and take precedent for decision-making in these mid-rectal tumors along with the MRI features (questions in this regard can be addressed by Dr. Smith (email above) or Dr. Horvat (email above) or both).

Table 11.2.2. Radiological criteria of suspicious lymph nodes on restaging MRI rectum.

**Anatomic Location**	**Restaging MRI rectum**
**TME** (mesorectal, superior rectal)	> 5 mm
**Internal iliac**	> 4 mm
**Obturator**	> 6 mm
**M1** (inguinal, external iliac, common iliac, retroperitoneal)	> 10 mm
**Mucin within the lymph node**	Suspicious regardless the size or location*

* MRI is unable to differentiate cellular from acellular mucin.

Table 11.2.3 Radiologic Features of Re-Growth

	**Signs of Re-Growth**
**MRI-T2W**	New Intermediate T2 Signal, No T2 Scar<br>AND/OR<br>New Lymph Nodes<br>AND/OR<br>Increased Size of Lymph Nodes
**MRI-DW**	New Signal on B800-B1000

11.3 Pathological Tumor Response at TME

For all patients who undergo surgery, all operative and pathology reports for the TME resection must be submitted. Reports must contain information about microscopic involvement of surgical resection margins.

11.3.1 Definition of Surgical Margin Status and Overall Completeness of TME Resection

Margin Type:

Proximal Margins	The proximal surgical margin refers to the most cephalad portion of the specimen (closest to patient's head).
Distal Margins	The distal margin refers to the most caudad portion (closest to anal canal).
Radial Margins	The radial margin, synonymously termed circumferential margin refers to the outer circumference of the rectal specimen.

Margin Positivity:

Positive	A surgical margin is POSITIVE if the pathologist notes tumor within ≤ 1mm of any edge of the primary tumor specimen.
Close	A surgical margin is CLOSE if the pathologist notes tumor > 1 but ≤ 3mm of any edge of the primary tumor specimen.
Negative	A surgical margin is NEGATIVE if the pathologist notes that there is NO tumor within 3mm of any edge of the primary tumor specimen.

Overall Completeness of TME Resection:

This categorization schema should focus on the pelvic resection of the TME specimen. If distant tumor is appreciated intraoperatively (for example, a liver metastasis), the pelvic resection may still be considered R0.

R0 resection	All gross disease has been removed, and microscopic examination reveals all surgical margins free of tumor. This must include the proximal, distal and radial margin.<br>Tumor > 1mm from the tumor resection margins is considered R_0. In some cases, intraoperatively, a surgeon may biopsy a liver nodule or retroperitoneal node. Resection will still be considered curative if pathologic examination reveals positive lymph nodes as long as the nodes were completely resected, unless there is also evidence of positive margins.
R1 resection	There is evidence of tumor manifest at 1 or more surgical resection margins based on microscopic pathologic assessment of the tumor specimen but there is no macroscopic evidence of tumor at any resection margin nor is there macroscopic evidence of residual tumor based on the surgeon's operative report.
R2 resection	The surgical pathologist identifies any macroscopic evidence of tumor at any of the surgical resection margins or there is macroscopic evidence of residual tumor based on the surgeon's operative report.
No resection	Removal of the primary tumor was not performed.

11.3.2 Degree of Pathological Treatment Response

<u>Pathological Complete Response (pCR):</u>

A pCR must include no gross or microscopic tumor identified anywhere within the surgical specimen. This must include: No evidence of malignant cells in the primary tumor specimen **AND** No lymph nodes that contain tumor.

<u>Pathological Response other than a Complete Response:</u>

The definition of a non-pCR will include any surgical specimen that has any evidence of residual tumor manifest in the primary or regional lymph nodes.

For patients who do not meet criteria for a pCR, the extent of response to pre-operative therapy will be graded using the Tumor Regression Grade (TRG) schema. This schema evaluates the degree to which the primary rectal tumor specimen has responded to neoadjuvant treatment.

<u>Tumor Regression Coding Based on AJCC 8th Edition</u>

Tumor Regression Grade (TRG)	Response Categorization	Description
TRG-0	Complete	No viable cancer cells
TRG-1	Moderate	Single cells or rare, small groups of cancer cells
TRG-2	Minimal	Residual cancer showing evident tumor regression, but more than single cells or rare small groups of cancer cells
TRG-3	Poor	Minimal or no tumor kill; extensive residual cancer and no evident tumor regression

11.4 Disease Evaluation after TME

Disease evaluation for all patients after completion of surgery. Patients will be evaluated as per the schedule of tests noted in Section 5.0. At the time of evaluation, patients will be classified in the following manner:

- No evidence of disease (NED)
- Recurrence of disease (REC): The appearance of rectal carcinoma in the primary site, nodal basin or distant organ sites during follow-up will be classified as recurrence cancer. Recurrence will be classified as local or distant.

**Local** recurrence	Any tumor located in the pelvis or the perineal scar **after TME** has been completed
**Distant** recurrence	Any tumor outside the pelvis and the perineal scar; can occur prior to or after TME

Patients may have local recurrence, distant recurrence, both or neither. Imaging should be used to document the extent of local and distant disease recurrence. Pathological confirmation of suspected distant metastasis should be performed based on clinical judgment and is not mandated.

Although pathologic confirmation of recurrence is not mandated, local and distant recurrences will be categorized based on supporting evidence. Recurrences will be categorized based on whether they are evident from:

**Pathology:**	Use this category if there is any pathology or cytology demonstrating recurrent rectal cancer.
**Serial Imaging:**	Use this category is for recurrences that have not been confirmed by pathology but where serial imaging studies (serial is defined as similar scans performed more than 30 days apart) indicate tumor.
**Clinical Evaluation:**	Use this category for recurrences that have not been confirmed by pathology or serial imaging studies but are manifest on physical exam or compelling imaging studies (absent serial images).

- Second primary colorectal cancer

Second primary cancers are those that arise anywhere in the colon. Tumors involving the surgical anastomosis or arising from the pelvis are to be categorized as recurrent disease.

12.0 END OF TREATMENT/INTERVENTION

12.1 Duration of Protocol Treatment

During neoadjuvant treatment, protocol treatment (intervention) is to continue until completion of LCRT and 8 cycles of consolidation chemotherapy, distant disease progression, local/regional disease progression which precludes surgery, or unacceptable adverse events. After neoadjuvant treatment, TME will be performed when it is appropriate (incomplete response to assigned TNT regimen (e.g. residual tumor remains, no evolution to complete response). Please see the study calendar (Section 5) and the treatment section (Section 7) for treatment and follow-up time periods.

12.2 Criteria for Discontinuation of Protocol Treatment/Intervention

In the absence of treatment delays due to adverse event(s), treatment may continue until one of the following criteria applies:

- Disease progression during neoadjuvant treatment which precludes surgery (e.g., tumor perforation that prohibits completion of treatment or treatment as designated per Arm or detection of distant metastases)
- Intercurrent illness that prevents further administration of treatment
- Unacceptable adverse event(s)
- Patient decides to withdraw from the study
- General or specific changes in the patient's condition render the patient unacceptable for further treatment in the judgment of the investigator
- Clinical progression
- Patient non-compliance
- Pregnancy (if applicable)
  - All women of child bearing potential should be instructed to contact the investigator immediately if they suspect they might be pregnant (e.g., missed or late menstrual period) at any time during study participation.
  - The investigator must immediately notify CTEP in the event of a confirmed pregnancy in a patient participating in the study.
- Termination of the study by sponsor

The reason(s) for protocol therapy discontinuation, the reason(s) for study removal, and the corresponding dates must be documented in the Case Report Form (CRF).

12.3 Follow-up

12.3.1 Duration of Follow- up (Please see study calendar in section 5.0 for complete list of follow-up tests and procedures)

	**MRI rectum**	**Endoscopy and digital rectal exam**	**CT chest, abdomen, pelvis**	**CEA**
All patients who completed pre-operative protocol treatment	Pre-treatment, post-TNT re-staging	Pre-treatment, post-TNT re-staging	Pre-treatment, Post-TNT re-staging, annually from 5 years post-registration	Pre-treatment, every 3-6 months post-TNT in Years 1-2, every 6 months post-TNT in Years 3-5
All patients who are off pre-operative protocol treatment early due to Progression of disease-	As per treatment team	As per treatment team	As per treatment team	As per treatment team
All patients who are off pre-operative protocol treatment early due to other reasons (patient refusal, clinician decision to withdraw/other)	As per treatment team	As per treatment team	As per treatment team	As per treatment team
Patients in WW group	After post-TNT re-staging: Q6 months in Years 1-2, Q12 months in Years 3-5 (As clinically indicated years 4-5)	Post-TNT re-staging: Q3 months in Years 1-2, Q6 months in Years 3-5	Pre-treatment, Post-TNT re-staging, annually from 5 years post-registration	Pre-treatment, every 3-6 months post-TNT in Years 1-2, every 6 months post-TNT in Years 3-5

12.4 Extraordinary Medical Circumstances

If, at any time the constraints of this protocol are detrimental to the patient's health and/or the patient no longer wishes to continue protocol therapy, protocol therapy shall be discontinued. In this event:

- Document the reason(s) for discontinuation of therapy on data forms.
- Follow the patient for protocol endpoints as required by the Study Calendar.

12.5 Managing ineligible patients and registered patients who never receive protocol intervention

Definition of ineligible patient

A study participant who is registered to the trial but does not meet all of the eligibility criteria is deemed to be ineligible.

Follow-up for ineligible patients who discontinue protocol treatment

For patients who are deemed ineligible after registering to the trial, who start treatment, but then discontinue study treatment, the same data submission requirements are to be followed as for those patients who are eligible and who discontinue study treatment.

Follow-up for patients who are registered, but who never start study treatment

For all study participants who are registered to the trial but who never receive study intervention (regardless of eligibility), the follow-up requirements are specified below.

Randomized phase II and phase III: Baseline, off treatment, and post-treatment follow up (i.e., relapse, progression, and survival) data submission required. See the Data Submission Schedule accompanying the All Forms Packet.

13.0 STATISTICAL CONSIDERATIONS

13.1 Study Endpoints

13.1.1 Primary Endpoints:

- Phase II: The primary endpoint is cCR rate. The cCR rate is defined as the number of patients who achieved cCR at the end of TNT divided by number of patients included in the analysis population (see Section 13.4.1). This endpoint will be assessed within 8-12 weeks after completion of TNT. If there is a cCR, then the patient will be counted in the numerator. If there is a near-complete response (nCR) then a re-evaluation within 4-8 weeks will be performed. If the tumor has evolved to a cCR, then the patient will be counted in the numerator. Otherwise, the patient will be deemed as NOT achieving cCR status.
- **Phase III:** Disease-free survival (DFS): is defined as the time from date of randomization to the date of first occurrence of the following events:
    - Death due to all causes
    - Tumor that recurs locally after an R0 TME
    - Tumor that regrows after an initial apparent clinical and radiological CR and cannot be surgically removed with an R0 TME*
    - M1 disease diagnosed at any point after the initiation of treatment

* Regrowth for patients who are on watch and wait approach (regardless of treatment assignment) that can be surgically removed with an R0 TME will NOT be a DFS event

13.1.2 Secondary Endpoints:

- Organ-preservation-time (OPT): is defined as time from the date of randomization to the date of the first occurrence of the following events:
    - o TME performed or attempted
    - o Tumor that regrows after an initial apparent clinical and radiological CR
    - o Death due to all causes
- Time to distant metastasis (TDM): is defined as time from the date of randomization to the date of first documented distant metastasis.
- Overall survival (OS): The OS is defined as time from the date of randomization to the date of death due to all causes.
- Adverse Events (AE) Rate: is defined as the proportion of patients experienced at least one Grade 3, Grade 4, or Grade 5 of each type of AE.

13.2 Sample Size

We anticipate enrolling a maximum of 760 patients (380 per arm). We anticipate an accrual rate of 180 patients per year (15 per month) based on similarly accruing studies of Alliance N1048 trial, NRG-GI002, and the OPRA trial. The total accrual period of this study is estimated to be 4.2 years, including start-up time.

For phase II portion, per study design, total of 296 evaluable patients (148 per arm) will be needed to evaluate the primary endpoint. Up to an additional 16 patients (5% inflation) might be accrued to account for cancelation after randomization and major violations. Thus, the total targeted accrual for phase II portion will be up to 312 patients.

13.3 Power Justification

The design of this trial is a randomized, seamless phase II/III trial. After a patient is registered, they will be assigned to one of the two treatment arms in a 1:1 ratio utilizing a dynamic allocation algorithm based on the methods by Pocock and Simon [46]..

The patients included in the phase II endpoints analyses will be included in the phase III endpoints analyses. The accrual will not be halted while waiting for phase II endpoints to be matured and analyzed, unless we experience unexpected rapid accrual and/or unacceptably high treatment toxicity, or other unforeseen situations. If the criteria for continuation have been satisfied at conclusion of phase II portion, to protect the phase III comparison, the actual results of the phase II portion will not be released beyond the Alliance DSMB.

13.3.1 Phase II Portion:

The phase II portion of this trial is designed to primarily compare cCR rate in patients with locally advanced rectal cancer treated with LCRT, mFOLFIRINOX, followed by a selective WW approach (Arm A) versus those who are treated with LCRT, mFOLFOX6, followed by a selective WW approach (Arm B). The Phase II portion of this trial implements a group sequential design with a single interim analysis for futility evaluation, adopting Rho family (Rho=2) beta spending function for controlling the overall type II error rate.

The OPRA trial reported 52.4% (87 out of 166 randomized to consolidation arm) of patients who achieved a sustained cCR and preserved the rectum. For the proposed trial, we assume a cCR rate of 50% in the control arm (Arm B). A total number of 296 evaluable patients will provide 90.5% power to detect an **17% increase** in cCR rate (67% in the experimental arm [Arm A]) at a one-sided type I error rate of 0.048. With the decision rules stated below in Section 6.4, this design provides the following design operation characteristics:

If the true cCR rate is …	H1<br>Experimental arm: 67%<br>Control arm: 50%	H0<br>Experimental arm: 50%<br>Control arm: 50%
Then the probability of stopping at the interim analysis due to futility is …	0.024	0.560
Then the probability of declaring that cCR rate of the experimental arm is superior to control arm is …	0.900	0.050

13.3.2 Phase III Portion:

The phase III portion of this trial is designed to primarily compare disease-free survival (DFS) in patients with locally advanced rectal cancer treated with LCRT, mFOLFIRINOX, followed by a selective WW approach (Arm A) versus those who are treated with LCRT, mFOLFOX6, followed by a selective WW approach (Arm B). Phase III portion implements a group sequential design with one futility interim analysis based on a non-binding beta spending function (Rho family with Rho=3.2), which will be performed when 50% of DFS events have been observed (143 events). The OPRA trial reported a three-year DFS rate of 76% (95% CI, 69-83%) for patients who received chemoradiotherapy followed by consolidation chemotherapy (CRT-CNCT)[47]. A total number of 285 DFS events will provide 85% power to detect an effect size of hazard ratio (HR) = 0.70 (3-year DFS rate of 82.5% in the experimental arm A) at a one-sided type I error rate of 0.025. With further assumptions of an accrual rate of 180 patients per year and a minimum of four years of follow-up, a maximum of 760 patients (380 in each arm) are required to enroll, unless the study team makes a decision of early termination due to monitoring rules specified in section of 13.4.2 or 13.5.1.

With the decision rules stated below in Section 13.4.2, this design provides the following design operation characteristics for interim analyses:

Analyses time point	If the true 3-year DFS rate is …	H1<br>Experimental arm: 82.5%<br>Control arm: 76%	H0<br>Experimental arm: 76%<br>Control arm: 76%
interim analysis (futility only) at 50% of events observed	Then the probability of stopping at the interim analysis due to futility is …	0.016	0.500

13.4 Statistical Analysis Plan

13.4.1 Analysis Populations:

- Intent-to-Treat (ITT) Population: ITT population includes all patients who are properly randomized, regardless of the actual treatment received. The treatment grouping will be according to the original assignment at randomization.
- Modified Intent-to-Treat (mITT) Population: mITT population includes all patients who are properly randomized, completed LCRT and started at least one dose of protocol defined chemotherapy treatment. The treatment grouping will be according to the original assignment at randomization.
- Per-Protocol (PP) Population: PP population includes all patients who are deemed eligible after randomization, properly randomized, and received at least three cycles of chemotherapy after LCRT. The treatment grouping will be according to the actual treatment received during the first cycle of the chemotherapy.
- Safety Population: Safety population includes all patient who received any dose of treatment defined by protocol.

13.4.2 Primary Endpoint:

Phase II Portion:

Hypothesis testing analyses will be performed on mITT population. Sensitivity analyses will be performed on PP population.

- *Interim Analysis Decision Rule*: An interim analysis will be performed when 50% (74 in each arm) of patients are randomized and cCR status available. Difference of proportions test[33], [34] will be conducted to compare cCR rate in the experimental arm to cCR rate in the control arm. The estimated one-sided p-value will be compared to the following stopping boundaries:

Testing:	One-sided P-value	Difference in proportions	Decisions
Futility	> 0.44	< 0.012	Reject H1: concluding that the chance of cCR rate of the experimental arm being superior to the control arm is too low to continue the study. The accrual will be terminated if it does not reach target enrollment.

  If futility boundary is not crossed, then the trial will continue accrual and patient follow-up per protocol. The trial will not be halted while performing the interim analysis. However, if the accrual is especially rapid, we may temporarily suspend accrual to avoid missing important acute toxicity patterns and assigning patients to potentially ineffective treatment (futility of cCR rate endpoint in experimental arm).

- *Final Analysis Decision Rule*: If the trial continues after the interim analysis, the final cCR rate analysis will be performed when target accrual is reached and the cCR status are available on all treated patients. Difference of proportions test will be conducted to compare cCR rate in the experimental arm to cCR rate in the control arm. If the one-sided p-value of the comparison is < 0.05 (difference in proportion > 9.3%), then we will conclude the cCR rate in the experimental arm is superior to the control arm.

Phase III Portion:

The Phase III hypothesis testing analyses will be performed on ITT population. Sensitivity analyses will be performed on mITT and PP population. At interim and final analyses, stratified Cox model will be conducted to compare DFS in the experimental arm to DFS in the control arm with stratification factors as stratum, based on all data collected at the analysis time point.

- *Interim Analysis Decision Rule:* The interim analysis will be conducted when 50% of targeted events (about 143) which is estimated around 4.5 to 4.9 years after the first patient is randomized (about 3.6 to 8.4 months after all patients are enrolled). If the observed HR (stratified by stratification factors) at interim analysis is > 1.00, we will conclude that the chance of DFS in experimental arm being superior to that in control arm is too low. The results will be presented to Alliance DSMB and NCI for potential public release.
- *Final Analysis Decision Rule*: The final DFS analysis will be performed when at least 285 DFS events are observed combining the two arms. Stratified Cox model will be conducted to compare DFS in the experimental arm A to DFS in the control arm B. If the one-sided p-value of the comparison is < 0.025, then we will conclude the DFS in the experimental arm is superior to the control arm.

13.4.3 Secondary Endpoints:

The analyses of secondary endpoints will be conducted on mITT and PP population whenever it is applicable and plausible. Safety-related analyses will be performed on the safety population.

The distributions of time-to-event endpoints will be estimated, in each arm, using the method of Kaplan-Meier and compared by a stratified Cox regression model. The binary endpoints will be estimated, in each arm, and compared by chi-squared test or Fisher's exact test. Dose intensity and number of cycles will be summarized by mean, standard deviation, median, Q1, Q3, and range, in each arm, and compared by Wilcoxon Rank Sum test. The maximum grade for each type of adverse events that are possibly, probably, or definitely related to study treatments will be recorded for each patient. The frequency tables will be reviewed to determine the patterns. The overall adverse event rates for grade 3 or higher adverse events will be compared between two treatment groups using Chi-square test (or Fisher's exact test if the data in the contingency table is sparse).

13.5 Monitoring

13.5.1 Safety Monitoring

Neoadjuvant treatment completion monitoring:

To maintain meaningful outcome comparisons between two arms, the completion of neoadjuvant treatment will be monitored. The early off treatment (EOT) rate will be defined as the number of patients who receive fewer than 8 cycles (e.g., 0 to 7 cycles) of neoadjuvant chemotherapy, due to disease progression, death, toxicity, treating physician discretion, and other disease related reasons, after LCRT divided by the number of patients who complete LCRT and start at least one dose of chemotherapy. Patients who withdraw from protocol treatment due to reasons that are not related to disease will be excluded from this analysis.. OPRA trial reported 7% (8/116) of patients who received < 8 cycles of FOLFOX after LCRT in consolidation arm.

We will compare EOT rates between two arms (experimental vs. control arms) when 88 (44 per arm), 174 (87 per arm) and 262 (131 per arm) evaluable patients complete

neoadjuvant chemotherapy or are off neoadjuvant chemotherapy early. Lan-DeMets OF spending function will be used to control multiple comparisons. If the difference in EOT rate (experimental arm minus control arm) is greater than 22.0%, 10.4% and 6.7% (corresponding p-value: < 0.001, 0.016, 0.045) at three time points, respectively, the treatment, toxicity and off treatment reasons data will be thoroughly reviewed and potential protocol modifications, including suggesting halting accrual, will be formulated under the consultations with CTEP. The total sample size of 262 patients will provide 80% power to detect a difference in EOT rate of 10% at a one-sided alpha of 0.05 significance level.

R0 resection for patients on WW strategy monitoring:

We will carefully monitor the R0 resection rate among patients who proceed to a WW strategy after TNT and later require TME during follow-up. Patients enrolled on two arms will be pooled for this monitoring. OPRA reported among 120 (out of 166 enrolled) patients in the CRT-CNCT arm who initially entered in the WW strategy, only 33 patients developed tumor regrowth. Four patients had local excision rather than TME and none required further treatment. Another four patients in the CRT-CNCT group recommended for TME declined any additional treatment. Three patients were not be able to undergo TME due to progressive disease. Therefore, only 22 patients (~13 to 15 % of enrolled patients) underwent TME on WW protocol. We estimate approximately 46 patients pooling two arms will have TME on WW following cCR deemed after TNT.

We consider a true R0 rate of 94% or higher (alternative hypothesis) to be adequate and 83% or lower (null hypothesis) to be inadequate. The following monitoring rules will provide 85.1% power to test the hypothesis whether the R0 resection rate in this small population is adequate, at a one-sided alpha of 0.086, with 56.6% likelihood of rejection at the first stage of 23 patients:

- When the first 23 patients who undergo TME on WW protocol had R0 resection data available, if 19 or fewer patients have R0 resection, OR
- when the first 46 who undergo TME on WW protocol had R0 resection data available, if 41 or fewer patients have R0 resection

then the treatment, toxicity and disease evaluation data will be thoroughly reviewed and potential protocol modifications, including suggesting halting accrual, will be formulated under the consultations with CTEP.

Tumor regrowth for patients on WW strategy monitoring:

We will carefully monitor the incidence of tumor regrowth among patients who proceed to a WW strategy after completion of pre-operative TNT. The OPRA trial reported a regrowth rate of 22% (95% CI, 14-29%, unpublished data) for patients randomized to CRT-CNCT who underwent the WW strategy, at one year of follow-up after completion of pre-operative TNT.

Rule # 1: Comparing 1-year tumor regrowth rates between two arms.

The analysis population of this monitoring rule is defined as patients who are included in the phase II portion final analysis AND achieve cCR status and choose WW strategy after completion of pre-operative TNT. The one-year regrowth rate will be defined as number of patients who experience tumor regrowth (regardless of whether it can be salvaged by TME) within one year after the last dose of pre-operative TNT divided by the total number of patients in the analysis population. The one-year regrowth rate will be estimated within each arm separately, when all patients in this population are followed for at least one year after last dose of chemotherapy. Based on Phase II design assumptions, the sample sizes

will be approximately 93 (=156*0.593, critical value to reject phase II null hypothesis) and 78 (=156*0.5, phase II control arm assumption) patients in Arm A and B, respectively.

DFS comparison between two arms will be noted to be concerning if the one-year regrowth rate in triplet TNT arm (Arm A) is much higher than that in the doublet TNT arm (Arm B). Thus, if:

- The difference in one-year regrowth rates (i.e., triplets arm minus doublets arm) is > 0.114, then the further accrual will be halted after discussion with CTEP.

This monitoring rule will provide 89% power to test the hypothesis that one-year regrowth rate in the triplet TNT arm (Arm A) is 42% versus that in the doublet TNT arm (Arm B) is 22%, i.e., 20% higher than that in doublet arm, at a one-sided alpha of 0.05.

Rule # 2: Comparing overall 1-year tumor regrowth rate (pooling two arms) to historical rate.

The goal of this rule is to guard against an excessive regrowth rate that may be seen across the various NCTN sites. The International W & W Database [48] reported a 15.5% 1-year regrowth rate. However, the patients included in this retrospective database were not treated with TNT and cCR was determined by various and heterogeneous methods. It should be noted that these patients are taken from highly selected institutional case series, there is no denominator, non-uniform definitions of response, relatively short clinical follow-up, inconsistent surveillance protocols and variable patient selection criteria and neoadjuvant treatment regimens. The 1-year regrowth rate from this population is very likely a underestimation and may not be compatible with the setting of a modern clinical trial. Therefore, relying on prospectively collected data with uniform selection criteria and surveillance strategies similar to our trial, we can take into account the OPRA data. The OPRA data does have the limitation of therapy provided at primarily tertiary centers, but does provide the advantage of a prospective cohort of patients treated with TNT and subsequently followed in a manner identical to what we have described for the Janus Rectal Cancer Trial. Therefore, we consider a 1-year rate of local regrowth of 22% observed from OPRA trial as the "upper limit" of safety benchmark, i.e., it is considered not safe for patients if the 1-year rate of local regrowth in JANUS trial is larger than this benchmark.

The same population (i.e., assuming total of 171 NOM patients) and definition of 1-year tumor regrowth rate in Rule #1 will be applied to Rule #2. We consider a true 1-year regrowth rate of 32% or higher (alternative hypothesis) to be unacceptable and 22% or lower to be acceptable. If the observed 1-year regrowth rate is > 28.2% (observed 6.2% increase), then the further accrual will be halted after discussion with CTEP. This monitoring rule will provide 85.6% power to detect if the true 1-year regrowth rate is unacceptable, i.e. 10% higher than historical rate of 22%, at a one-sided alpha of 0.025.

Patient safety monitoring:

The safety monitoring rule specified below is based on OPRA data and the knowledge available at study development. We note that the patient safety monitoring rule may be adjusted in the event of either 1) the study re-opening to accrual (if paused) or 2) at any time during the conduct of the trial and in consideration of newly acquired information regarding the adverse event profile of the treatment(s) under investigation.

- If at any time in either arm we observe at least one of the following scenarios, then discussions among the Study Team, Alliance DSMB, and CTEP are required to assess whether modifications to the study are needed:

- During TNT treatment, in each arm separately, if 6 or more patients in the first 30 treated patients (or > 20% of all patients after 30 are accrued) experience a grade 4 or higher non-hematologic adverse event deemed at least possibly related to study treatment (i.e. an adverse event with attribute specified as "possible," "probable," or "definite").

The Study Chair(s) and the Study Statistician will review the study monthly to identify accrual, adverse event/safety trends, and any emerging concerns.

13.5.2 Data and Safety Monitoring Board

This study will be monitored by the Alliance Data and Safety Monitoring Board (DSMB), an NCI-approved functioning body. Reports containing efficacy, adverse event, and administrative information will be provided to the DSMB every six months as per NCI guidelines

13.5.3 Data Mapping Utility (DMU)

This study has been assigned Demography monitoring.

Required submission of patient demographic data for this study will be submitted automatically via OPEN.

Note: Serious adverse events must be submitted via CTEP-AERS per protocol guidelines.

13.6 Inclusion of Women and Minorities

This study will be available to all eligible patients, regardless of race, gender, or ethnic origin. There is no information currently available regarding differential effects of this regimen in subsets defined by race, gender, or ethnicity, and there is no reason to expect such differences to exist. Therefore, although the planned analysis will, as always, look for differences in treatment effect based on racial and gender groupings, the sample size is not increased in order to provide additional power for subset analyses. The geographical region served by the Alliance, has a population which includes approximately 18% minorities. Based on prior Alliance studies involving similar disease sites, we expect about 21% of patients will be classified as minorities by race and about 45% of patients will be women. Expected sizes of racial by gender subsets for patients registered randomized to this study are shown in the following table.

DOMESTIC PLANNED ENROLLMENT REPORT					
Racial Categories	Ethnic Categories				Total
	Not Hispanic or Latino		Hispanic or Latino		
	Female	Male	Female	Male	
American Indian/ Alaska Native	5	10	0	0	15
Asian	14	17	2	3	36
Native Hawaiian or Other Pacific Islander	5	6	0	1	12
Black or African American	36	46	6	7	95
White	239	290	34	36	599
More Than One Race	1	1	1	0	3
**Total**	300	370	43	47	760

14.0 CORRELATIVE AND COMPANION STUDIES

14.1 Correlative Science

Testing of banked samples will not occur until an amendment to this treatment protocol (or separate correlative science protocol) is reviewed and approved in accordance with National Clinical Trials Network (NCTN) policies. All laboratory correlates are classified as exploratory, and the specimens requested for submission in Section 6.2 will be collected for banking-only at this time. These future studies may include the studies described below.

14.1.1 Background

14.1.1.1 Genomic analyses to profile rectal cancer treated with neoadjuvant chemoradiation and chemotherapy

There are currently no histopathologic or molecular biomarkers of response to TNT. Thus, there is an unmet need to identify and validate biomarkers that can be studied in future trials to personalize treatment approaches to facilitate organ preservation and maximize likelihood of cure. To assess this tumor mutation, copy number alteration, and gene expression profiling analyses may be performed on pretreatment tumor biopsies of patients treated in both study arms. Emerging technologies may allow us to perform temporospatial single cell sequencing. As such tissue blocks are strongly encouraged to maximize our ability to carry out these analyses.

14.1.1.2 Circulating tumor DNA to monitor tumor response to TNT in rectal cancer patients treated in both protocol arms

Circulating tumor DNA in Rectal Cancer may be prognostic

Circulating tumor DNA (ctDNA) has recently emerged as a promising non-invasive biomarker of disease status in cancer patients. The majority of patients with mCRC (80-98%) have detectable ctDNA levels at time of diagnosis.[2-5] Presence of ctDNA after surgical resection in stages I to III CRC has identified patients with a high risk of relapse.[6-8] While studies evaluating ctDNA in patients with rectal cancer undergoing total neoadjuvant therapy are limited, there appears to be a signal of clinical utility [38, 39,]. Multiple groups have reported worse recurrence free survival in patients with detectable ctDNA post chemoradiation [38, 40-43].

14.1.2 Objectives

The recent NCI Colon and Rectal-Anal Task Forces whitepaper outlined areas of investigation for future neoadjuvant clinical trials in rectal cancer should include: (1) ctDNA at time of diagnosis, (2) changes in ctDNA throughout neoadjuvant therapy to assess markers of response, (3) ctDNA post-definitive treatment and (4) utility of ctDNA as a marker of minimal residual disease during surveillance. Future exploratory studies may address the following objectives based on specimen availability:

1) Are tumor informed assays feasible in NCTN rectal cancer trials?
2) What percent of patients will have detectable ctDNA at baseline?
3) What percent of patients will enter molecular remission post…
    a. Induction chemoradiation?
    b. Induction chemoradiation and consolidative chemotherapy?
4) Does molecular complete response associate with clinical complete response?
5) Do patients with molecular residual disease post-TNT have worse progression free survival?

6) Does the presence of molecular residual disease in patients with a complete clinical response who opt for WW associate with…
    a. Higher rates of recurrence?
    b. Higher rates of local regrowth?
    c. Higher rates of distant metastases in those patients with either local recurrence after TME or in those who have local regrowth?

14.1.3 Methods

As this is a rapidly evolving field with new ctDNA assays in development, the study team has determined our preference to encourage prospective tissue and blood banking to allow us to evaluate and select the most appropriate assay based on the sample availability utilizing best in class technology at that time.

15.0 MONITORING PLAN

Standard Alliance monitoring procedures will be used for this study.

16.0 REFERENCES

[1] J. Garcia-Aguilar *et al.*, “Preliminary results of the organ preservation of rectal adenocarcinoma (OPRA) trial.,” *J. Clin. Oncol.*, vol. 38, no. 15_suppl, pp. 4008–4008, May 2020.

[2] C. Cremolini *et al.*, “Individual Patient Data Meta-Analysis of FOLFOXIRI Plus Bevacizumab Versus Doublets Plus Bevacizumab as Initial Therapy of Unresectable Metastatic Colorectal Cancer.,” *J. Clin. Oncol.*, p. JCO2001225, Aug. 2020.

[3] J. B. Bachet *et al.*, “FOLFIRINOX as induction treatment in rectal cancer patients with synchronous metastases: Results of the FFCD 1102 phase II trial.,” *Eur. J. Cancer*, vol. 104, pp. 108–116, 2018.

[4] E. Fokas *et al.*, “Randomized Phase II Trial of Chemoradiotherapy Plus Induction or Consolidation Chemotherapy as Total Neoadjuvant Therapy for Locally Advanced Rectal Cancer: CAO/ARO/AIO-12,” *J. Clin. Oncol.*, p. JCO.19.00308, May 2019.

[5] R. D. James *et al.*, “Mitomycin or cisplatin chemoradiation with or without maintenance chemotherapy for treatment of squamous-cell carcinoma of the anus (ACT II): a randomised, phase 3, open-label, 2×2 factorial trial,” *Lancet Oncol.*, vol. 14, no. 6, pp. 516–524, May 2013.

[6] J. Garcia-Aguilar *et al.*, “Effect of adding mFOLFOX6 after neoadjuvant chemoradiation in locally advanced rectal cancer: a multicentre, phase 2 trial.,” *Lancet. Oncol.*, vol. 16, no. 8, pp. 957–66, Aug. 2015.

[7] A. Falcone *et al.*, “Phase III trial of infusional fluorouracil, leucovorin, oxaliplatin, and irinotecan (FOLFOXIRI) compared with infusional fluorouracil, leucovorin, and irinotecan (FOLFIRI) as first-line treatment for metastatic colorectal cancer: the Gruppo Oncologico Nord Ovest.,” *J. Clin. Oncol.*, vol. 25, no. 13, pp. 1670–6, May 2007.

[8] F. Loupakis *et al.*, “Initial therapy with FOLFOXIRI and bevacizumab for metastatic colorectal cancer.,” *N. Engl. J. Med.*, vol. 371, no. 17, pp. 1609–18, Oct. 2014.

[9] C. Cremolini *et al.*, “FOLFOXIRI plus bevacizumab versus FOLFIRI plus bevacizumab as first-line treatment of patients with metastatic colorectal cancer: updated overall survival and molecular subgroup analyses of the open-label, phase 3 TRIBE study.,” *Lancet. Oncol.*, vol. 16, no. 13, pp. 1306–15, Oct. 2015.

[10] T. Gruenberger *et al.*, “Bevacizumab plus mFOLFOX-6 or FOLFOXIRI in patients with initially unresectable liver metastases from colorectal cancer: the OLIVIA multinational randomised phase II trial.,” *Ann. Oncol. Off. J. Eur. Soc. Med. Oncol.*, vol. 26, no. 4, pp. 702–708, Apr. 2015.

[11] H. Schmoll *et al.*, “FOLFOX/bevacizumab +/- irinotecan in advanced colorectal cancer (CHARTA): Long term outcome,” *Ann. Oncol.*, vol. 29, p. v108, Jun. 2018.

[12] H. I. Hurwitz *et al.*, “Phase II Randomized Trial of Sequential or Concurrent FOLFOXIRI-Bevacizumab Versus FOLFOX-Bevacizumab for Metastatic Colorectal Cancer (STEAM).,” *Oncologist*, vol. 24, no. 7, pp. 921–932, 2019.

[13] J. Sastre *et al.*, “Randomized phase III study comparing FOLFOX + bevacizumab versus folfoxiri + bevacizumab (BEV) as 1st line treatment in patients with metastatic colorectal cancer (mCRC) with ≥3 baseline circulating tumor cells (bCTCs).,” *J. Clin. Oncol.*, vol. 37, no. 15_suppl, pp. 3507–3507, May 2019.

[14] E. Van Cutsem *et al.*, “ESMO consensus guidelines for the management of patients with metastatic colorectal cancer.,” *Ann. Oncol. Off. J. Eur. Soc. Med. Oncol.*, vol. 27, no. 8, pp. 1386–422, 2016.

[15] A. B. Benson *et al.*, “Colon Cancer, Version 2.2021, NCCN Clinical Practice Guidelines in Oncology.,” *J. Natl. Compr. Canc. Netw.*, vol. 19, no. 3, pp. 329–359, 2021.

[16] T. Yoshino *et al.*, "Pan-Asian adapted ESMO consensus guidelines for the management of patients with metastatic colorectal cancer: a JSMO-ESMO initiative endorsed by CSCO, KACO, MOS, SSO and TOS.," *Ann. Oncol. Off. J. Eur. Soc. Med. Oncol.*, vol. 29, no. 1, pp. 44–70, 2018.

[17] P. Rouanet *et al.*, "Tailored Treatment Strategy for Locally Advanced Rectal Carcinoma Based on the Tumor Response to Induction Chemotherapy: Preliminary Results of the French Phase II Multicenter GRECCAR4 Trial.," *Dis. Colon Rectum*, vol. 60, no. 7, pp. 653–663, Jul. 2017.

[18] T. Conroy *et al.*, "Neoadjuvant chemotherapy with FOLFIRINOX and preoperative chemoradiotherapy for patients with locally advanced rectal cancer (UNICANCER-PRODIGE 23): a multicentre, randomised, open-label, phase 3 trial.," *Lancet. Oncol.*, vol. 22, no. 5, pp. 702–715, 2021.

[19] R. Sauer *et al.*, "Preoperative versus postoperative chemoradiotherapy for rectal cancer.," *N. Engl. J. Med.*, vol. 351, no. 17, pp. 1731–40, Oct. 2004.

[20] R. Glynne-Jones, M. Harrison, and R. Hughes, "Challenges in the neoadjuvant treatment of rectal cancer: balancing the risk of recurrence and quality of life.," *Cancer Radiother.*, vol. 17, no. 7, pp. 675–85, Nov. 2013.

[21] M. Maas *et al.*, "Long-term outcome in patients with a pathological complete response after chemoradiation for rectal cancer: a pooled analysis of individual patient data.," *Lancet. Oncol.*, vol. 11, no. 9, pp. 835–44, Sep. 2010.

[22] A. Dewdney and D. Cunningham, "Toward the non-surgical management of locally advanced rectal cancer.," *Curr. Oncol. Rep.*, vol. 14, no. 3, pp. 267–76, Jun. 2012.

[23] M. H. Martens *et al.*, "Long-term Outcome of an Organ Preservation Program After Neoadjuvant Treatment for Rectal Cancer.," *J. Natl. Cancer Inst.*, vol. 108, no. 12, Dec. 2016.

[24] M. J. M. van der Valk *et al.*, "Long-term outcomes of clinical complete responders after neoadjuvant treatment for rectal cancer in the International Watch & Wait Database (IWWD): an international multicentre registry study," *Lancet*, vol. 391, no. 10139, pp. 2537–2545, Jun. 2018.

[25] J. J. Smith *et al.*, "Assessment of a Watch-and-Wait Strategy for Rectal Cancer in Patients With a Complete Response After Neoadjuvant Therapy," *JAMA Oncol.*, p. e185896, Jan. 2019.

[26] J. J. Smith *et al.*, "Organ Preservation in Rectal Adenocarcinoma: a phase II randomized controlled trial evaluating 3-year disease-free survival in patients with locally advanced rectal cancer treated with chemoradiation plus induction or consolidation chemotherapy, and total," *BMC Cancer*, vol. 15, no. 1, p. 767, Jan. 2015.

[27] A. Cercek *et al.*, "Adoption of Total Neoadjuvant Therapy for Locally Advanced Rectal Cancer," *JAMA Oncol.*, Mar. 2018.

[28] A. J. Franke, H. Parekh, J. S. Starr, S. A. Tan, A. Iqbal, and T. J. George, "Total Neoadjuvant Therapy: A Shifting Paradigm in Locally Advanced Rectal Cancer Management," *Clin. Colorectal Cancer*, vol. 17, no. 1, pp. 1–12, Mar. 2018.

[29] T. Conroy *et al.*, "Total neoadjuvant therapy with mFOLFIRINOX versus preoperative chemoradiation in patients with locally advanced rectal cancer: Final results of PRODIGE 23 phase III trial, a UNICANCER GI trial.," *J. Clin. Oncol.*, vol. 38, no. 15_suppl, pp. 4007–4007, May 2020.

[30] G. Hospers *et al.*, "Short-course radiotherapy followed by chemotherapy before TME in locally advanced rectal cancer: The randomized RAPIDO trial.," *J. Clin. Oncol.*, vol. 38, no. 15_suppl, pp. 4006–4006, May 2020.

[31] R. R. Bahadoer *et al.*, "Short-course radiotherapy followed by chemotherapy before total mesorectal excision (TME) versus preoperative chemoradiotherapy, TME, and optional adjuvant chemotherapy

in locally advanced rectal cancer (RAPIDO): a randomised, open-label, phase 3 trial," *Lancet Oncol.*, vol. 0, no. 0.

[32] A. Habr-Gama *et al.*, "Operative versus nonoperative treatment for stage 0 distal rectal cancer following chemoradiation therapy: long-term results.," *Ann. Surg.*, vol. 240, no. 4, pp. 711–7; discussion 717-8, Oct. 2004.

[33] C. Jennison and B. W. Turnbull, *Group Sequential Methods with Applications to Clinical Trials*. CRC Press, 1999.

[34] D. J. Sheskin, *Handbook of Parametric and Nonparametric Statistical Procedures*, 3rd ed. Boca Raton, FL.: Chapman & Hall/CRC Press, 2004.

[35] L. K. Temple *et al.*, "The Development of a Validated Instrument to Evaluate Bowel Function After Sphincter-Preserving Surgery for Rectal Cancer," *Dis. Colon Rectum*, vol. 48, no. 7, pp. 1353–1365, Jul. 2005.

[36] K. J. Emmertsen and S. Laurberg, "Low anterior resection syndrome score: development and validation of a symptom-based scoring system for bowel dysfunction after low anterior resection for rectal cancer.," *Ann. Surg.*, vol. 255, no. 5, pp. 922–8, May 2012.

[37] M. M. Russell *et al.*, "Comparative effectiveness of sphincter-sparing surgery versus abdominoperineal resection in rectal cancer: patient-reported outcomes in National Surgical Adjuvant Breast and Bowel Project randomized trial R-04.," *Ann. Surg.*, vol. 261, no. 1, pp. 144–8, Jan. 2015.

[38] J. Tie *et al.*, "Serial circulating tumour DNA analysis during multimodality treatment of locally advanced rectal cancer: a prospective biomarker study.," *Gut*, vol. 68, no. 4, pp. 663-671, Apr. 2019.

[39] S. Khakoo *et al.*, "MRI Tumor Regression Grade and Circulating Tumor DNA as Complementary Tools to Assess Response and Guide Therapy Adaptation in Rectal Cancer.," *Clin Cancer Res.*, vol. 26 no.1, pp. 183-192, Jan. 2020.

[40] Y. Wang *et al.*, "Utility of ctDNA in predicting response to neoadjuvant chemoradiotherapy and prognosis assessment in locally advanced rectal cancer: A prospective cohort study.," *PLoS Med.*, vol. 18 no.8, Aug. 2021.

[41] A. Boysen *et al.*, "Cell-free DNA and preoperative chemoradiotherapy for rectal cancer: a systematic review.," *Clin Transl Oncol.*, vol. 21 no. 7, Jul. 2019.

[42] D. Massihnia, *et al.*, "Liquid biopsy for rectal cancer: A systematic review.," *Cancer Treat Rev.*, doi: 10.1016/j.ctrv.2019.101893, Sep. 2019.

[43] M. Morais, *et al.*, "ctDNA on liquid biopsy for predicting response and prognosis in locally advanced rectal cancer: A systematic review.," *Eur J Surg Oncol.*, vol. 48, no.1, Jan. 2022.

[44] Daprà, V., Airoldi, M., Bartolini, M., Fazio, R., Mondello, G., Tronconi, M. C., ... & Santoro, A. (2023). Total Neoadjuvant Treatment for Locally Advanced Rectal Cancer Patients: Where Do We Stand?. *International Journal of Molecular Sciences*, *24*(15), 12159.

[45] Verheij, F. S., Omer, D. M., Williams, H., Lin, S. T., Qin, L. X., Buckley, J. T., ... & Garcia-Aguilar, J. (2023). Long-Term Results of Organ Preservation in Patients With Rectal Adenocarcinoma Treated With Total Neoadjuvant Therapy: The Randomized Phase II OPRA Trial. *Journal of Clinical Oncology*, JCO-23.

[46] Pocock, S. J., & Simon, R. (1975). Sequential treatment assignment with balancing for prognostic factors in the controlled clinical trial. *Biometrics*, 103-115.

[47] Garcia-Aguilar, J., Patil, S., Gollub, M. J., Kim, J. K., Yuval, J. B., Thompson, H. M., ... & Saltz, L. B. (2022). Organ preservation in patients with rectal adenocarcinoma treated with total neoadjuvant therapy. *Journal of Clinical Oncology*, *40*(23), 2546-2556.

[48] van der Valk MJM, Hilling DE, Bastiaannet E, Meershoek-Klein Kranenbarg E, Beets GL, Figueiredo NL, Habr-Gama A, Perez RO, Renehan AG, van de Velde CJH; IWWD Consortium. Long-term outcomes of clinical complete responders after neoadjuvant treatment for rectal cancer in the International Watch & Wait Database (IWWD): an international multicentre registry study. Lancet. 2018 Jun 23;391(10139):2537-2545. doi: 10.1016/S0140-6736(18)31078-X. PMID: 29976470.

APPENDIX I: RECOMMENDED CLINICAL MANAGEMENT OF DIARRHEA

Pharmacologic diarrhea management

- For patients with persistent grade 1 diarrhea on loperamide, diphenoxylate hydrochloride and atropine sulfate (Lomotil®) 1 tablet every 6 to 8 hours may be added.
- For ≥ grade 2 diarrhea despite intensive antidiarrheal therapy, consider adding octreotide (short acting) 150 micrograms subcutaneous injection as needed up to three times per day; or after the initial dose of short acting octreotide, if well tolerated, a single dose of octreotide LAR 20 mg IM.
- For grade 3 or grade 4 diarrhea with complicating features (dehydration, fever, and/or grade 3-4 neutropenia)
  - Administer loperamide: initial dose of 4 mg (2 tablets/capsules) with the first bout of diarrhea followed by 2 mg (1 tablet/capsule) every 4 hours or after every unformed stool (maximum 16 mg a day) and continue loperamide at this frequency until diarrhea free for 12 hours. Then titrate the amount of loperamide used to keep diarrhea controlled (< 4 stools/day).
  - Administer octreotide (100-150 µg SC BID or [25–50 µg/hr IV if dehydration is severe, with dose escalation up to 500 µg SC TID).
  - Use IV therapy as appropriate.
  - Stool cultures should be done to exclude infectious causes of grade 3 or 4 diarrhea or diarrhea of any grade with complicating features (dehydration, fever, and/or grade 3 or 4 neutropenia) per the Investigator's discretion.
  - Consider prophylactic antibiotics as needed (e.g., fluoroquinolones) especially if diarrhea is persistent beyond 24 hours or there is fever or grade 3-4 neutropenia.
- Patients should be monitored for constipation and prophylaxis adjusted accordingly. Do not discontinue antidiarrheals completely; doses may be adjusted.
- For the second and subsequent cycles, the dose of loperamide should be titrated to keep diarrhea controlled to < 4 stools a day.

Dietary management

Instruct patients to:

- Stop all lactose-containing products (milk, yogurt, cheese, etc.).
- Drink 8-10 large glasses (64-80 ounces) of clear liquids per day.
- Eat frequent small meals.
- Maintain a low fat diet enriched with rice, bananas, applesauce, and/or toast.

APPENDIX II: LEVOLEUCOVORIN DRUG DOSE AND ADMINISTRATION INSTRUCTIONS

Levoleucovorin can be substituted for racemic leucovorin (leucovorin) throughout this protocol, per institutional practice or as needed for drug availability.

Dose:

Levoleucovorin **200 mg/m^2**

(Note: The levoleucovorin dose is one-half the dose of leucovorin.)

Reconstitute as described in the manufacturer's full prescribing information. Do not round the dose.

Administration (per institutional practice with guidance below):

Further dilute the reconstituted levoleucovorin dose with 250 mL D5W.

Using separate infusion bags and separate lines utilizing Y-connector tubing, administer levoleucovorin IV over 2 hours concurrently with oxaliplatin (mFOLFOX6). If oxaliplatin is held, administer levoleucovorin over 2 hours (preferred); however, administration time for all chemotherapy in this protocol per institutional practice is permitted.

Due to poor absorption at doses greater than 50 mg, **the use of oral leucovorin is not permitted**.

The decision and rationale for administering levoleucovorin must be documented in the patient's medical record.

Changing the patient's treatment in any way other than as stated above will be considered non-protocol therapy and result in a protocol violation

APPENDIX III PATIENT MEDICATION DIARIES

CAPECITABINE PATIENT DIARY (CONSOLIDATION CHEMOTHERAPY)

Page 1 of 1

Protocol: JANUS Rectal Cancer Trial (A022104)
Study Medication: Capecitabine

Cycle #__________

Prescribed dose: capecitabine ____________mg twice each day on days 1 to 14 of each 21 day cycle

- Please record information daily.
- Take capecitabine in the morning and evening (within 30 minutes after eating breakfast and dinner).
- If a dose is missed for any reason, skip the dose and take the next dose at the normal time. Do not double up doses.
- Please remember to bring this diary (all pages) If a dose is missed for any reason, skip the dose and take the next dose at the normal time. Do not double up doses.

Day	Date	Time taken		Number of capecitabine tablets taken (morning)		Number of capecitabine tablets taken (evening)		Notes<br>Include any side effects that you are having (especially loose stools and any medications that you took for the side effects.
		Morning	Evening	150mg	500mg	150mg	500mg	
1								
2								
3								
4								
5								
6								
7								
8								
9								
10								
11								

**12**								
**13**								
**14**								
**15**								
**16**								
**17**								
**18**								
**19**								
**20**								
**21**								

Patient's name: ________________________________ Date: _____________________

Physician's office will complete this section

Total number of capecitabine tablets taken this cycle: 150 mg _____500 mg _______

Research Staff Signature/Date: ____

CAPECITABINE PATIENT DIARY (CHEMORADIATION)

Page 1 of 1

Protocol: JANUS Rectal Cancer Trial (A022104)
Study Medication: Capecitabine

Cycle #__________

Prescribed dose: capecitabine ____________mg twice each day on days of radiation

- Please record information daily.
- Use a new page for each week of treatment/cycle.
- Take capecitabine in the morning and evening (within 30 minutes after eating breakfast and dinner).
- If a dose is missed for any reason, skip the dose and take the next dose at the normal time. Do not double up doses.
- Please remember to bring this diary (all pages) to each visit with your study team or as directed (at the end of the chemoradiation)

Day	Date	Time taken		Number of capecitabine tablets taken (morning)		Number of capecitabine tablets taken (evening)		Notes: Include any side effects that you are having (especially loose stools and any medications that you took for the side effects.
		Morning	Evening	150mg	500mg	150mg	500mg	
1								
2								
3								
4								
5								
6**								
7**								

Patient's name: _______________________________ Date: ____________________

Physician's office will complete this section

Total number of capecitabine tablets taken this cycle: 150 mg _____500 mg _______

Research Staff Signature/Date: ____

** Day 6 and 7 should be left blank unless told otherwise by your treating physician
